# Explainable Physicochemical Determinants of Protein–Ligand Binding via Non-Covalent Interactions

**DOI:** 10.64898/2026.03.03.707476

**Published:** 2026-03-05

**Authors:** Zhaohan Meng, Zhen Bai, Ke Yuan, Jaime H. Cheah, Wei Jiang, Adam Skepner, Kevin J. Leahy, Iadh Ounis, William M. Oldham, Zaiqiao Meng, Hao Xu, Joseph Loscalzo

**Affiliations:** 1 School of Computing Science, University of Glasgow; 2 School of Life Science and Technology, Institute of Science Tokyo; 3 School of Cancer Sciences, University of Glasgow; 4 Cancer Research UK Scotland Institute; 5 Center for the Development of Therapeutics, The Broad Institute of MIT and Harvard; 6 Department of Medicine, Brigham and Women’s Hospital, Harvard Medical School, Harvard University; 7 Department of Medicine, The Warren Alpert Medical School of Brown University; 8 The Broad Institute of MIT and Harvard; 9 Language Technology Lab, University of Cambridge

## Abstract

Protein–ligand binding (PLB) underlies a broad range of biological and chemical processes, including enzymatic catalysis, metabolic regulation, molecular recognition, and therapeutic modulation. These processes are governed by structured constellations of non-covalent interactions—such as hydrogen bonding, electrostatic interactions, hydrophobic contacts, and aromatic stacking—that determine binding affinity and functional specificity. However, many AI-based models prioritize predictive performance, with limited emphasis on mechanistic interpretation of the underlying interactions. As a result, existing approaches often function as black-box predictors limiting their utility for mechanistic reasoning, functional modulation, and generalization to novel protein–ligand pairs. Here, we introduce **ExplainBind**, an interaction-aware sequence-based framework that jointly predicts protein–ligand binding likelihood and learns residue-level interaction patterns grounded in physical non-covalent interactions. To enable explicit supervision, we construct **InteractBind**, a curated database of protein–ligand complexes from which residue-atom interaction maps are systematically extracted. ExplainBind aligns token-level cross-attention with these interaction maps, enabling physically grounded and mechanistically interpretable predictions without requiring explicit three-dimensional inputs at inference time. Across extensive in-distribution and out-of-distribution evaluations, ExplainBind consistently outperforms existing sequence-based baselines and demonstrates improved robustness to protein and ligand distribution shifts. Quantitative analyses and structural case studies show that the learned interaction maps accurately localize binding pockets and recover known interaction motifs. We further validate the framework by effectively ranking highly potent inhibitors for angiotensin-converting enzyme (ACE) and discovering both inhibitors and activators of the metabolic enzyme L-2-hydroxyglutarate dehydrogenase (L2HGDH), illustrating how ExplainBind supports functional modulation beyond binary binding prediction. Together, these results establish ExplainBind as a scalable and interpretable paradigm for protein–ligand binding prediction across drug discovery, enzyme engineering, and broader molecular design applications.

## 1 Introduction

The machinery of life is orchestrated through intricate networks of molecular interactions [1, 2]. Enzymes accelerate chemical reactions by stabilizing transition states, signaling proteins transmit information through transient contacts, and therapeutics achieve efficacy by engaging precise binding sites [3]. Underlying these processes is a constellation of noncovalent forces: hydrogen bonds [4], salt bridges [5], van der Waals contacts [6], hydrophobic contacts [7], π–π stacking [8], and cation–π interactions [9] that collectively dictate affinity, specificity, and kinetics. Acting in concert, these interactions stabilize enzymatic catalysis, organize macromolecular assemblies, and confer selectivity to drugs [10]. Crucially, they are exquisitely sensitive to select structural determinants: the loss of a single hydrogen bond, the reorientation of an aromatic ring, or the disruption of a hydrophobic patch can shift a system from active to inert, or from selective to promiscuous. Non-covalent interactions are, therefore, not incidental features, but the fundamental determinants of molecular recognition across biology and medicine [11, 12].

Molecular interaction modeling has long relied on three-dimensional structural representations, where atomic geometries explicitly encode non-covalent forces. Classical physics-based approaches, including molecular docking [13, 14] and molecular dynamics simulations [15], directly model interaction energies from atomic coordinates to rationalize binding and specificity. More recently, geometric deep learning has enabled data-driven modeling of protein–protein interactions (PPI), exemplified by MaSIF [16–18], which learns molecular surface interaction fingerprints at protein–protein interfaces. Related ideas have since been adapted to protein–ligand systems, giving rise to distance-aware geometric models for docking [19–25]. Although distance-based geometric information serves as an implicit proxy for molecular interactions in these approaches, underlying non-covalent interaction types are not directly given. Consistent with this limitation, recent adversarial analyses of representative protein–ligand co-folding models, including AF3 [26], RoseTTAFold All-Atom [27], Chai-1 [28] and Boltz-1 [29], demonstrate that high structural accuracy does not guarantee physically meaningful interactions, revealing systematic violations of steric and electrostatic principles despite low root-mean-square deviation (RMSD) predictions [30]. Notably, this gap is not confined to structure-based models [31, 32], but also arises in sequence-based models [33–37], where molecular interactions are not explicitly represented.

In the absence of explicit physically grounded interaction modeling, the field has converged on protein–ligand binding (PLB) prediction as a scalable surrogate objective, using binding outcomes as a functional summary of complex and unobserved molecular interaction processes [38, 39]. Sequence-based PLB models typically employ cross-attention or related interaction fusion modules to infer latent residue–atom correspondences directly from protein sequences and ligand representations [40–46]. Related sequence-driven binding-site inference methods [47–49] similarly identify interaction-relevant residues from sequence representations, employing convolutional or attention-based architectures. While effective for prediction, these interaction representations are typically optimized solely for end-task performance and lack explicit supervision grounded in physical interactions. As a result, they often function as black boxes, offering limited mechanistic interpretability and weak correspondence to the non-covalent forces that govern binding. To date, PLB prediction has not been coupled with explicit supervision of interaction learning using physically grounded non-covalent interaction maps.

**Figure 1:**
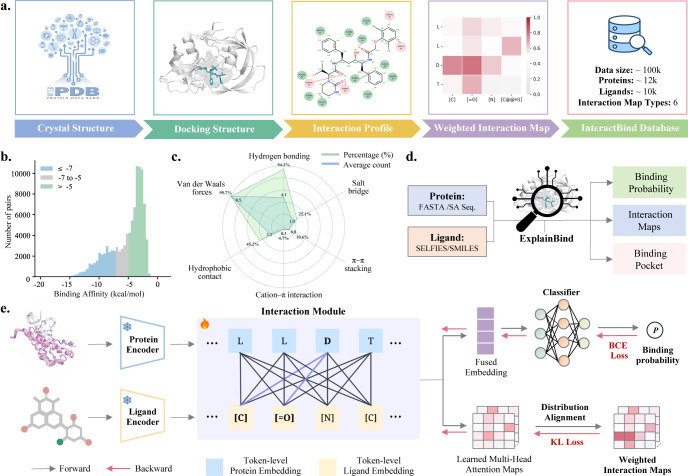
Interaction-aware database construction and ExplainBind framework. **a.** InteractBind construction. PDB-derived complexes and randomly paired protein–ligand pairs are docked and filtered by docking score to retain high-confidence positives and negatives. Non-covalent interactions are identified using rule-based geometric criteria and encoded as distance-weighted, token-level interaction maps aggregated across interaction types. **b.** Distribution of binding energy for all protein–ligand pairs derived from docking. **c.** Relative frequencies and average counts of six interaction types. **d.** Inference pipeline of ExplainBind. Users provide protein and molecule sequences to predict binding probabilities and generate interaction diagrams of the binding pocket. **e.** Training strategy of ExplainBind. A BCE loss supervises binary binding prediction, and a KL divergence loss aligns learned attention with ground-truth interaction energy maps.

To address this challenge, we introduce ExplainBind, a novel explainable protein–ligand binding prediction framework that learns token-level interactions by supervising cross-attention with continuous non-covalent interaction maps (Fig. 1). By explicitly grounding attention in non-covalent interactions, ExplainBind aligns learned interaction representations with underlying physical interaction patterns, jointly optimizing predictive accuracy and mechanistic interpretability. This interaction-level supervision moves beyond black-box attention mechanisms, enabling predictions that are both accurate and physically interpretable. Integrating the scalability of sequence-based representations with physically grounded interaction supervision, ExplainBind enables accurate and interpretable protein–ligand binding prediction at proteome and chemical-library scales where high-resolution structural information is sparse. Under in-distribution (ID) and out-of-distribution (OOD) settings, ExplainBind achieves state-of-the-art predictive performance and maintains strong robustness. Moreover, its learned attention maps enable reliable localization of protein binding pockets, facilitating mechanistic interpretation and effective prioritization of high-activity ligands. In the case of angiotensin-converting enzyme (ACE), the model produces calibrated probability estimates that align with (retrospective) activity measurements, enabling systematic prioritization of high-affinity ligands. As a prospective application, we identify both inhibitors and activators of the metabolic enzyme L-2-hydroxyglutarate dehydrogenase (L2HGDH), demonstrating how ExplainBind translates predictive signals into actionable insights for drug discovery.

## 2 Results

### 2.1 Overview of ExplainBind

**ExplainBind** is an end-to-end protein–ligand binding framework that jointly predicts binding probability while explicitly modeling the physicochemical interactions underlying molecular recognition. The framework combines frozen foundation encoders for proteins and ligands with an interaction module that performs token-level cross-attention between amino acid residues and ligand atoms, and is directly supervised using interaction-type–specific maps derived from curated protein–ligand complexes. Accordingly, ExplainBind is jointly optimized with two complementary objectives: a Binary Cross-Entropy (BCE) loss [50] on the predicted binding probability and a Kullback–Leibler divergence (KL) loss [51] aligning the learned attention maps with ground-truth interaction maps (Fig. 1c). This interaction-aware supervision aligns learned representations with concrete non-covalent interactions—such as hydrogen bonds, salt bridges, van der Waals contacts, hydrophobic contacts, π–π stacking and cation–π interactions—thereby grounding predictions in biologically meaningful residue–ligand relationships. This design allows ExplainBind to achieve strong predictive performance while producing inspectable interaction maps.

To train ExplainBind, we curate the **InteractBind** database from the Protein Data Bank (PDB) [52] (Fig. 1a). Instead of labeling all resolved complexes as positives and randomly paired protein–ligand pairs as negatives, we stratify pairs by docking score (kcal/mol), retain only high-confidence negatives (≥ −5) and positives (≤ −7) for supervision, and exclude intermediate-affinity cases to reduce label ambiguity (Fig. 1b). Non-covalent interactions are identified using rule-based criteria grounded in geometric and chemical constraints, with interaction-specific distance cutoffs and optional angular filters applied to ensure validity (Supplementary Table S1). Interaction strength is modeled by a distance-dependent piecewise linear decay function that maps interatomic or geometric distances within the cutoff window to continuous values in [10^−6^, 1]. These strengths define an m×n interaction map for each interaction type, where m and n denote the numbers of ligand tokens and protein tokens, respectively, and each entry represents the interaction strength between a ligand token and a protein residue. Interaction maps from all types are then aggregated to produce a single combined map, yielding an integrated, token-level representation of protein–ligand interaction patterns.

In the following analyses, we evaluate the framework under both **ID** and **OOD** settings, demonstrating that explicit interaction supervision improves generalization to unseen proteins and ligands while yielding attention patterns consistent with established biochemical principles. For the OOD setting, we construct similarity-controlled evaluation scenarios along two complementary axes. Ligand chemical similarity is quantified using Tanimoto similarity over extended connectivity fingerprints (ECFP) [53], yielding mean train–test similarities of 8%, 35%, 40%, and 59%, with protein similarity fixed at 33% across all splits. Protein similarity is quantified using global sequence alignment based on the Needleman–Wunsch algorithm [54], yielding peak train–test similarities of 25%, 28%, 31%, and 33%. Combining these regimes results in eight OOD datasets spanning progressively increasing degrees of chemical and sequence novelty. For evaluation, we employ five-fold cross-validation, and performance is reported as the mean ± standard deviation across the five-fold scale. Model performance is primarily assessed using the area under the receiver operating characteristic curve (AUROC), the area under the precision–recall curve (AUPRC), and accuracy (Acc) [55]. In addition, we report the F1 score, sensitivity, specificity, and Matthews correlation coefficient (MCC) [44]. Comprehensive details of the datasets, baseline models, and hyperparameters are provided in Supplementary sections S.1.1, S.1.4 and S.1.5.

### 2.2 Evaluation of ExplainBind under in-distribution setting

ExplainBind outperforms eight representative baseline methods under the ID split setting, including classical machine-learning models—Support Vector Machine [56] and Random Forest [57]—as well as state-of-the-art deep learning approaches for protein–ligand binding prediction: DeepConv-DTI [58], GraphDTA [59], MolTrans [60], DrugBAN [61], LANTERN [62], and GraphBAN [63], as shown in Table 1. ExplainBind consistently achieves the strongest performance in all evaluation metrics, with AUROC and AUPRC reaching 0.993 and 0.954, respectively, representing absolute improvements of 1.1 and 1.0 points over the best competing baseline, along with higher F1 and MCC scores and a more balanced sensitivity–specificity profile. These results indicate robust decision boundaries under the ID regime and reflect ExplainBind’s interaction-aware training strategy in which token-level interaction is explicitly supervised using residue-level interaction annotations spanning multiple physicochemical interaction types. By aligning learned representations with underlying binding mechanisms rather than dataset-specific correlations, ExplainBind establishes a strong ID performance baseline and motivates its evaluation under controlled distribution shifts in subsequent OOD analyses, demonstrating superior robustness under distribution shift.

### 2.3 Evaluation of ExplainBind under out-of-distribution setting

**Figure 2:**
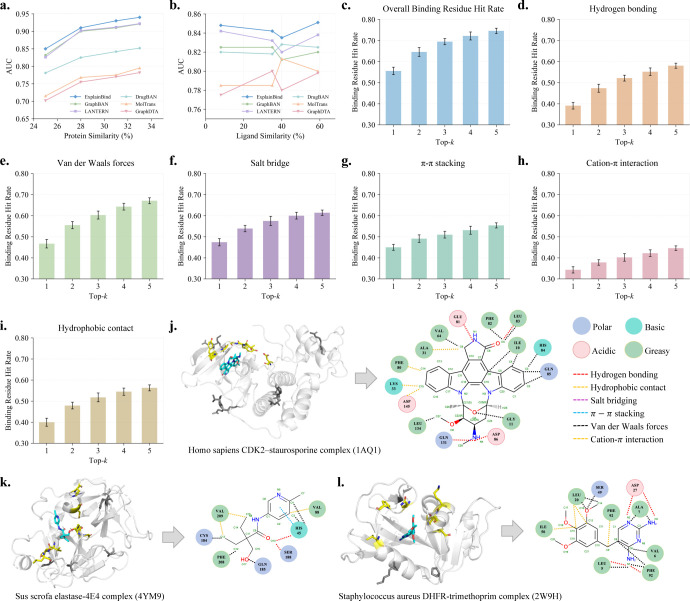
Out-of-distribution performance and interaction-level interpretability of ExplainBind. **a.** OOD performance under protein sequence similarity–controlled splits, with AUC increasing consistently as sequence similarity increases. **b.** OOD performance under ligand similarity–controlled splits, showing stable AUC across decreasing chemical similarity thresholds. **c–i**. Top-*k* binding-pocket hit-rate accuracy for all interactions combined and for individual interaction types. **j–l**. Case studies of representative protein–ligand complexes (PDB: 1AQ1, 4YM9, and 2W9H). Interaction maps derived from crystal structures are compared with model predictions. The top 20 predicted residues recover all experimentally observed contacts. Ligands are shown in cyan, the top 1 predicted residue is highlighted in yellow and resides within the experimental binding pocket, whereas lower-ranked residues among the top 20 that fall outside the pocket are shown in black.

To assess model robustness more rigorously, we further investigate protein-ligand interaction prediction under the OOD setting, where the training and test sets contain non-overlapping proteins. This setting more closely reflects practical discovery pipelines, where candidate targets may share limited similarity with previously studied proteins. Under OOD evaluation, predictive performance decreases markedly compared with ID settings, highlighting the difficulty of generalizing to previously unseen protein–ligand pairs as shown in Fig. 2 a, b. Notably, however, performance degradation remains relatively moderate across methods, even at low sequence similarity levels. Despite this increased challenge, ExplainBind consistently achieves the strongest results across all OOD datasets and evaluation metrics (Details are provided in Supplementary section S11), demonstrating superior robustness under distribution shift.

Further analysis reveals that protein sequence similarity is the primary determinant of OOD generalization. While model performance remains largely stable across a broad range of ligand similarity thresholds, a clear and monotonic improvement is observed as protein sequence similarity increases. This behavior is consistent across all evaluated methods, with ExplainBind maintaining a systematic advantage at every similarity level. Compared with related tasks such as protein–protein interaction prediction [64], where performance often degrades sharply under comparable sequence divergence, the relatively mild degradation observed here highlights the stability of PLB prediction under protein-controlled OOD settings. These observations identify protein sequence divergence as the dominant bottleneck for generalization in PLB prediction and underscore the importance of protein-controlled OOD benchmarks for realistic evaluation.

### 2.4 Binding residue hit rate

Binding residue localization is evaluated to quantify whether predicted interaction maps highlight functionally relevant protein residues. For each protein–ligand pair, ExplainBind produces head-specific interaction attention maps over ligand–protein token pairs. Residue localization performance is assessed using a Top-*k* hit-based metric, termed the *Binding Residue Hit Rate* (BRHR), which records whether at least one protein residue among the Top-*k* highest-scoring interactions corresponds to a ground-truth interacting residue (formal definition in Supplementary section S.1.3). Using this protocol, Fig. 2c shows that the overall BRHR increases monotonically with *k*, rising from 55.6% at Top-1 to 74.6% at Top-5. This trend indicates that as more high-confidence interaction evidence is considered, true binding residues are increasingly implicated in the model’s strongest predictions, demonstrating robust residue-level localization under a strictly exact-matching criterion.

We further stratify BRHR by interaction type to attribute this overall trend to specific physical interaction cues (Fig. 2d–i). Van der Waals forces and salt bridges achieve the highest accuracy across most Top-*k* thresholds, likely reflecting their prevalence and well-defined geometric characteristics in protein–ligand complexes (Fig. 1c). Hydrogen bonding and hydrophobic contacts also show strong and consistent improvements as *k* increases. In contrast, less frequent interaction types, such as π–π stacking and cation–π interactions, exhibit lower absolute BRHR under exact matching, highlighting the increased difficulty of precisely localizing these uncommon interaction residues. Numerical results for all interaction types are provided in Supplementary Table S4.

When the matching criterion is relaxed to allow a small positional tolerance (window = 1; Supplementary Table S5), BRHR increases consistently across all *k* values and interaction categories, with absolute gains of approximately 6–9 percentage points. This relaxed setting corresponds to binding pocket localization, where functionally relevant sites typically comprise short contiguous residue segments rather than single exact positions. The observed improvement therefore suggests that the predicted interaction attention maps are spatially coherent and well-suited for practical binding pocket identification.

### 2.5 Case studies of binding pocket discovery

A further strength of ExplainBind lies in its capacity to provide residue-level explanations that are quantitatively verifiable against ground-truth interaction maps. By decomposing the interaction module’s attention maps into type-specific components (hydrogen bonds, hydrophobic interactions, π–π stacking, cation–π interactions, salt bridges, and van der Waals interactions), the model visualizes how individual residues contribute to each interaction type. For each complex, we highlight the top 20 residues receiving the highest attention weights, corresponding to those fragments annotated as experimentally confirmed contacts. We analyze three co-crystallized protein–ligand complexes from different organisms: *Homo sapiens* (CDK2–staurosporine; PDB: 1AQ1) [65], *Sus scrofa* (porcine pancreatic elastase–4E4; PDB: 4YM9) [66], and *Staphylococcus aureus* (DHFR–trimethoprim; PDB: 2W9H) [67]. For each co-crystallized complex, the top 20 residues identified by ExplainBind covered all interaction sites observed in the crystal structures, indicating complete recall of true contacts, while the top 1 model-predicted residues highlight the binding pocket. A detailed analysis is presented below:

- **Homo sapiens CDK2–staurosporine** (Fig. 2j). The model correctly highlighted three interaction types—hydrogen bonds, hydrophobic interactions, and van der Waals interactions—that collectively anchor staurosporine within the CDK2 binding cleft. Key hydrogen bond donors or acceptors included GLU81, LEU83, ASP86, and GLN131, engaging the ligand’s indolocarbazole backbone in close agreement with crystallographic data. Hydrophobic interactions were concentrated around ALA31, LYS33, PHE80, and ASP145, forming a compact apolar cage that stabilizes the planar ring system. In addition, ILE10, GLY11, VAL64, PHE82, LEU83, HIS84, GLN85, and LEU134 contributed to van der Waals interactions along the aromatic scaffold, further reinforcing ligand binding within the pocket.
- **Sus scrofa elastase–4E4** (Fig. 2k). The ground-truth interaction map comprises four interaction types—hydrogen bonds, hydrophobic interactions, π–π stacking, and van der Waals interactions—all of which were accurately recovered by ExplainBind. The model identified hydrogen bonds between the inhibitor’s carbonyl and amide groups and residues GLN185, SER188, and HIS45 within the catalytic triad region. Hydrophobic contacts clustered around VAL88, VAL209, and PHE208 provide the principal non-polar anchoring surface. Notably, HIS45 was also enriched in π–π stacking, highlighting dual aromatic–electrostatic stabilization within the substrate-recognition pocket. In addition, HIS45, CYS184, and GLN185 contributed to van der Waals interactions, further reinforcing local packing between the ligand and the catalytic groove.
- **Staphylococcus aureus DHFR–trimethoprim** (Fig. 2l). The model successfully reproduced the hydrogen bond network between the ligand’s amino and methoxy groups and the enzyme’s active-site residues LEU5, ASP27, and PHE92. Hydrophobic interactions were correctly localized to ILE50, LEU20, and PHE92, which surround the *p*-aminobenzyl ring of trimethoprim and stabilize its orientation within the pocket. Additionally, LEU5, VAL6, ALA7, LEU20, SER49, and PHE92 were predicted to contribute to van der Waals interactions, collectively forming a mixed polar–apolar microenvironment characteristic of the DHFR binding site.

### 2.6 Prioritization of high-activity ligands

A key application of predictive binding models is the prioritization of active ligands from large candidate pools. To evaluate ExplainBind under this prioritization setting, we performed retrospective enrichment analysis using angiotensin-converting enzyme (ACE), a well-characterized therapeutic target with extensive publicly available bioactivity data, and prospective prioritization followed by experimental validation on the previously unseen metabolic enzyme L-2-hydroxyglutarate dehydrogenase (L2HGDH). Additional background on these two targets is provided in Supplementary section S.1.6. Notably, neither ACE nor L2HGDH was included in the InteractBind training set, and neither has closely related homologs in the training data, enabling evaluation of ExplainBind on unseen targets. These analyses assess whether predicted binding probabilities enable reliable prioritization of functional ligands in both retrospective and prospective settings.

#### Retrospective prioritization on ACE.

**Figure 3:**
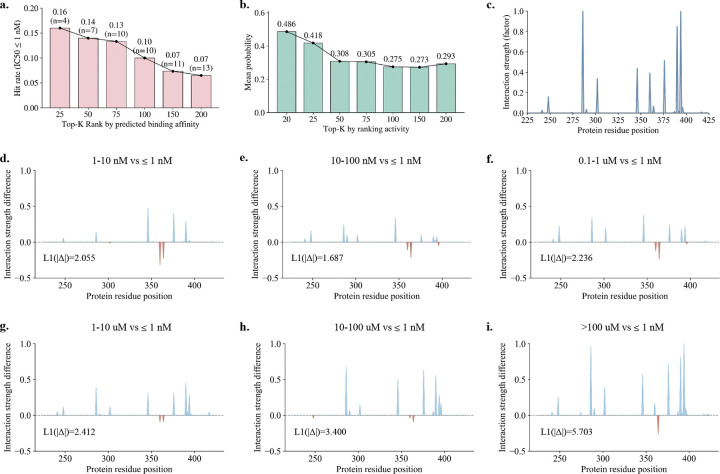
Affinity-stratified binding performance and residue-level interaction profiles for ACE. **a**, Hit rate for highly potent compounds (IC_50_ ≤ 1 nM) exhibits a smooth, monotonic decay as a function of Top-*K* rank based on predicted binding probability (*K* ≤ 200), indicating consistent prioritisation of true binders among top-ranked candidates (numbers denote sample counts per bin). **b,** Mean predicted binding probability decreases with increasing Top-K rank when compounds are ranked by experimental activity, reflecting calibrated model confidence across the ranking spectrum. **c,** Residue-level interaction map for ACE predicted by the proposed model, highlighting putative binding residues along the protein sequence. **d–i,** Differential residue-level interaction strengths (Δ) between affinity strata and the IC_50_ ≤ 1 nM reference group. Positive and negative values indicate strengthened or weakened interactions relative to the ≤ 1 nM group. The $\ell_{1}$ norm of Δ quantifies the overall deviation in interaction patterns as binding affinity decreases.

ACE inhibition is a well-established therapeutic strategy for the treatment of hypertension and heart failure [68, 69]. We, therefore, examined whether ExplainBind can enrich highly potent ACE inhibitors when compounds are ranked by predicted binding probability. As shown in Fig. 3a, the hit rate for ultra-potent ligands (IC_50_ ≤ 1 nM) exhibits a smooth and monotonic decay as the ranking cutoff Top-*K* increases from 25 to 200, indicating strong early enrichment followed by gradual inclusion of weaker binders. The Top-25 ranked compounds achieve a hit rate of 0.16, demonstrating that highly potent ligands are preferentially concentrated among top-ranked candidates. Importantly, this decay behavior is continuous rather than abrupt, suggesting that ExplainBind preserves a stable ranking structure across a broad candidate range. Consistent with this observation, when compounds are ranked by experimental activity, the mean predicted binding probability decreases smoothly with increasing Top-*K* (Fig. 3b), dropping from 0.486 at Top-20 to approximately 0.293 at Top-200. This trend indicates that predicted binding probabilities are aligned with experimental potency across the ranking spectrum, supporting their use for prioritization.

Beyond compound-level ranking, ExplainBind provides residue-level interaction profiles that reveal affinity-dependent interaction patterns. The predicted interaction map for ACE (Fig. 3c) highlights a sparse subset of residues with strong interaction contributions. To quantify how interaction patterns change with affinity, we compared residue-level interaction strengths across progressively weaker affinity bins relative to the IC_50_ ≤ 1 nM reference group (Fig. 3d–i). For compounds with nanomolar potency, interaction differences remain localized and modest, reflected by relatively low $\ell_{1}$ norms of the differential residue-level interaction strengths Δ (1.687–2.236). As affinity decreases into the micromolar regime, interaction differences become increasingly pronounced and spatially distributed, with the $\ell_{1}$ norm increasing to 3.400 for 10–100 *μ*M and reaching 5.703 for compounds with IC_50_ > 100 *μ*M. These progressive deviations indicate systematic changes in residue-level interaction patterns associated with reduced binding potency. Together, these results demonstrate that ExplainBind enables prioritization of highly potent ACE ligands while capturing affinity-dependent interaction signatures at the residue level.

#### Prospective prioritization and experimental validation on L2HGDH.

**Figure 4:**
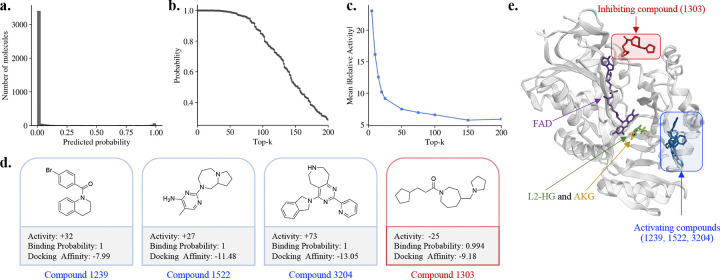
Experimental validation. **a.** Distribution of predicted binding probabilities across the internal compound pool (3,750 compounds), broadly consistent with the experimentally observed activity spectrum. **b.** The binding probability ranking exhibits a quick decay from 1 to 0.3 for the top 200 compounds. **c.** The mean |Relative Activity| within the top 200 compounds indicates a significant enrichment of active compounds among the highly ranked candidates. **d.** The highly active ligands within the Top-50 compounds, illustrating scaffold diversity among both experimentally validated inhibitors and activators. **e.** Docking analysis of representative ligands in the L2HGDH binding site (PDB: 8W78, chain A). Docked poses are superimposed on the protein backbone (schematic representation with semi-transparent surface). The substrate L-2-hydroxyglutarate (L2-HG, green) and product α-ketoglutarate (AKG, yellow) are shown for reference. Activators (compounds 1239, 1522, and 3204) are shown in blue, the inhibitor (compound 1303) in red, and the flavin adenine dinucleotide (FAD) cofactor in purple.

In contrast to ACE, where inhibition is the primary therapeutic objective, modulation of L2HGDH activity in either direction may be biologically relevant [70–74]. We, therefore, evaluated whether ExplainBind can prioritize compounds that functionally modulate L2HGDH activity, including both inhibitors and activators. ExplainBind was applied to an internal pool of 3,750 compounds to predict binding probability and guide experimental selection. As shown in Fig. 4a, the majority of compounds are assigned low predicted probabilities (below 0.2), indicating that strong binders are rare within the screened chemical space. As shown in Fig. 4b, predicted binding probabilities decrease monotonically with rank, spanning from 1.0 to approximately 0.3 across the Top-200 compounds, motivating their selection for experimental testing. Relative activity was defined as the percent change in enzyme activity in the presence of compound relative to the vehicle control. Additional details on assay conditions and activity calculations are provided in Supplementary section S.1.8. In parallel, Fig. 4c demonstrates strong early enrichment: the Top-5 ranked compounds achieve a mean |Relative Activity| of 22.40, computed as the average activity across the Top-*k* candidates. Mean |Relative Activity| decreases rapidly with increasing rank, falling to 9.65 at the Top-20 limit and reaching a shallow tail of approximately 5–7 by the Top-200, consistent with progressive inclusion of a larger pool of weaker modulators. Together, these trends indicate a coherent transition from high-confidence, high-activity candidates to lower-confidence predictions while preserving ranking consistency.

Experimental measurements confirm strong enrichment of functional modulators among top-ranked compounds. Among the Top-50 ranked compounds, four compounds exhibit high experimental activity (|Relative Activity| ≥ 25), including three activators and one inhibitor (Fig. 4d). These compounds represent chemically diverse scaffolds and are all assigned high predicted binding probabilities, demonstrating that ExplainBind successfully prioritizes functionally active ligands across different mechanisms of action. Docking analysis further supports these findings by revealing distinct binding modes for representative compounds (Fig. 4e). Inhibitory ligands preferentially occupy a pocket proximal to the FAD cofactor, consistent with potential disruption of catalytic redox processes, whereas activating ligands bind at spatially distinct sites, suggesting possible allosteric regulation. These results demonstrate that ExplainBind enables prospective prioritization of functional ligands on an unseen target and that top-ranked predictions are enriched for experimentally validated modulators.

## 3 Discussion

The performance of ExplainBind demonstrates that grounding sequence-based prediction in physical interaction principles yields robust and mechanistically interpretable representations. By supervising token-level interaction learning with continuous non-covalent interaction maps derived from experimentally resolved complexes, ExplainBind moves beyond implicit attention-based heuristics and establishes a direct connection between sequence-derived representations and the physical determinants of binding. This result shows that mechanistic interaction modeling and scalable sequence-based prediction are not mutually exclusive, even in the absence of explicit three-dimensional inputs.

A key insight from our evaluation is that interaction grounding improves generalization under distribution shift. Across systematic (ID) and OOD benchmarks stratified by protein and ligand similarity, ExplainBind maintains competitive performance as sequence and chemical similarity decrease. Notably, protein sequence similarity emerges as the dominant limiting factor for generalization, whereas ligand similarity plays a comparatively smaller role. This observation is consistent with biological intuition: binding interfaces and interaction patterns are primarily encoded in protein sequences, while ligands often manifest greater chemical variability. The robustness of ExplainBind under challenging OOD settings suggests that the model captures transferable interaction principles rather than relying on dataset-specific correlations or shortcut features.

Beyond predictive accuracy, ExplainBind enables direct mechanistic interpretation through its learned interaction maps. These maps reliably localize binding pockets and identify residue-level contributions to binding, allowing binding hypotheses to be examined even when resolved complex structures are unavailable. This capability bridges the gap between prediction and mechanism, extending sequence-based PLB models from ranking tools to hypothesis-generating frameworks. The practical value of this interpretability is illustrated by its ability to rank ligand potency across a broad activity spectrum for the clinically important target ACE, and by our experimental validation on the metabolic enzyme L2HGDH, where ExplainBind successfully discovers both inhibitory and activating ligands with high activity. These results highlight the utility of interaction-grounded models for guiding experimental design and decision-making.

Taken together, our findings suggest a shift in how scalable PLB prediction can be approached. Rather than treating interaction modeling and interpretability as secondary by-products of structural analysis, physically grounded interaction supervision can be directly integrated into sequence-based frameworks. This paradigm combines the scalability required for proteome- and chemical-library–wide screening with the mechanistic transparency traditionally associated with structure-based methods. As experimentally annotated interaction data continue to expand, interaction-grounded sequence models such as ExplainBind provide a promising foundation for robust, explainable, and experimentally actionable protein–ligand binding prediction.

## 4 Methods

### 4.1 Problem Formulation

In a PLB prediction task, the objective is to determine whether a given protein–ligand pair interacts, while additionally predicting residue-level interaction maps for specific physicochemical interaction types. For proteins, the input is sequence 𝒫 composed of residue-level tokens, and for ligands, the input is a molecular sequence 𝒟 representing atom-level structure. Based on these representations, ExplainBind jointly predicts binding likelihood and residue-level interaction maps that localize how individual residues contribute to binding. Formally, ExplainBind learns a mapping

$$f_{\theta }:(𝒫,𝒟)⟶\left(p,\left\{{\overset{ˆ}{A}}^{\left(t\right)}\right\}\right),$$

where p∈[0,1] denotes the predicted binding likelihood of the protein–ligand pair, and ${\overset{ˆ}{A}}^{\left(t\right)}$ denotes the residue-level interaction map corresponding to physicochemical interaction type t. Based on these outputs, ExplainBind jointly predicts binding likelihood and residue-level interaction maps that localize how individual residues contribute to binding.

### 4.2 Model Architecture

#### 4.2.1 Encoders

Capturing rich and biologically grounded representations is essential for exploring fine-grained and explainable protein–ligand interactions. For ligands, SMILES strings are widely used but may contain invalid segments and lose critical structural cues [75]. To overcome these limitations, we adopt SELFIES [75], a robust molecular grammar that guarantees valid molecular graphs and preserves atom-level information [75]. For proteins, we primarily use FASTA sequences, in which each token corresponds to one of the 23 standard amino acid symbols. When reliable structural information is available, ExplainBind can optionally incorporate structure-aware (SA) protein representations derived via *Foldseek* [76], providing explicit local structural context [77].

Foundation models have shown strong capability in encoding contextual and structural information through large-scale pre-training on biological corpora. The framework combines frozen foundation encoders for proteins and ligands, enabling ExplainBind to leverage powerful pretrained representations without updating the backbone models. In our default configuration, we use ESM-2 [78] as the frozen protein encoder and SELFormer [79] as the frozen ligand encoder for SELFIES representations. These encoders produce token-level protein embeddings P and ligand embeddings D that serve as inputs to the interaction module, forming the basis for accurate binding prediction and explainable encoders, including SaProt [77] when SA representations are used, as well as ProteinBERT [80], MoLFormer [81], and ChemBERTa-2 [82], with results summarized in Supplementary S9.

#### 4.2.2 Interaction Module

Unlike prior unsupervised models for PLB prediction [43], ExplainBind explicitly supervises attention with ground-truth maps derived from experimentally resolved protein–ligand complexes. Concretely, we convert residue–level contacts in each complex into per-type interaction maps and use them to directly optimise the attention computed from protein and ligand embeddings. To our knowledge, our proposed framework is the first to use complex-level attention maps to guide token-level fusion of proteins and ligands. Unsupervised attention conflates heterogeneous physical forces and tends to produce diffuse, non-identifiable alignments. By supervising attention with per-type contact maps, we (a) disambiguate interaction mechanisms (e.g., hydrogen bond vs. hydrophobic contact), (b) improve the faithfulness of explanations, and (c) use the same supervision signal to shape the fused token embeddings towards binding-relevant substructures.

Let $D\in R^{m\times h}$ and $P\in R^{n\times h}$ denote the token-level embeddings of the ligand and protein, respectively. Linear projections generate query (Q), key (K), and value (V) matrices for each modality:

$$Q_{d}=DW_{q}^{d},\;K_{d}=DW_{k}^{d},\;V_{d}=DW_{v}^{d},\;Q_{p}=PW_{q}^{p},\;K_{p}=PW_{k}^{p},\;V_{p}=PW_{v}^{p},$$

where $W_{\left(\cdot \right)}^{\left(\cdot \right)}\in R^{h\times h}$ are learnable projections.

The interaction module uses multi-head attention with H=8 heads: six heads correspond to specific interaction types, and two heads capture overall binding patterns. For each head t∈𝒯, bidirectional cross-attention computes the residue–level interaction intensity (where h is the hidden dimension size):

$${\overset{ˆ}{A}}_{pd}^{\left(t\right)}=Softmax\left(\frac{Q_{p}^{\left(t\right)}K_{d}^{(t{)}^{⊤}}}{\sqrt{h_{t}}}\right),\;{\overset{ˆ}{A}}_{dp}^{\left(t\right)}=Softmax\left(\frac{Q_{d}^{\left(t\right)}K_{p}^{(t{)}^{⊤}}}{\sqrt{h_{t}}}\right),$$

where ${\overset{ˆ}{A}}_{pd}^{\left(t\right)}\in R^{n\times m}$ and ${\overset{ˆ}{A}}_{dp}^{\left(t\right)}\in R^{m\times n}$. Self-attention is simultaneously applied to refine intra-sequence contexts:

$$P_{sa}^{\left(t\right)}=Softmax\left(\frac{Q_{p}^{\left(t\right)}K_{p}^{(t{)}^{⊤}}}{\sqrt{h_{t}}}\right)V_{p}^{\left(t\right)},\;D_{sa}^{\left(t\right)}=Softmax\left(\frac{Q_{d}^{\left(t\right)}K_{d}^{(t{)}^{⊤}}}{\sqrt{h_{t}}}\right)V_{d}^{\left(t\right)}.$$

Type-specific fused representations are obtained by combining self- and cross-attention outputs:

$$P^{*(t)}=\frac{1}{2}\left[P_{sa}^{\left(t\right)}+{\overset{ˆ}{A}}_{pd}^{\left(t\right)}V_{d}^{\left(t\right)}\right],\;D^{*(t)}=\frac{1}{2}\left[D_{sa}^{\left(t\right)}+{\overset{ˆ}{A}}_{dp}^{\left(t\right)}V_{p}^{\left(t\right)}\right].$$

Finally, outputs from all heads are concatenated and projected to yield the fused token sequences:

$$P^{*}={Concat}_{t\in 𝒯}\left(P^{*(t)}\right)W_{p}^{o},\;D^{*}={Concat}_{t\in 𝒯}\left(D^{*(t)}\right)W_{d}^{o}.$$

#### 4.2.3 Classifier

Given the fused token embeddings $P^{*}\in R^{n\times d}$ and $D^{*}\in R^{m\times d}$, we form a pair-level representation by mean pooling over tokens and concatenating the pooled vectors:

$${\bar{P}}^{*}=\frac{1}{n}\sum_{j=1}^{n}P_{j}^{*},\;{\bar{D}}^{*}=\frac{1}{m}\sum_{i=1}^{m}D_{i}^{*},\;F=[{\bar{D}}^{*},{\bar{P}}^{*}].$$

The binding probability is predicted by a lightweight MLP head, p=MLP(F).

#### 4.2.4 Training objective

Let y∈{0,1} denote the ground-truth binding label and p the predicted binding probability. The model is optimised using a binary cross-entropy classification loss,

$${ℒ}_{cls}=-\left[y\log p+\left(1-y\right)\log\left(1-p\right)\right].$$

When interaction supervision is available, we further align the predicted cross-attention maps with ground-truth interaction annotations. For each supervised head $t\in {𝒯}_{\text{sup}}$, the predicted attention ${\overset{ˆ}{A}}^{\left(t\right)}$ and the ground-truth interaction map $A^{\left(t\right)}$ are normalised over residue–atom pairs to form discrete distributions, ${\tilde{\overset{ˆ}{A}}}^{\left(t\right)}$ and ${\tilde{A}}^{\left(t\right)}$, respectively. The attention alignment loss is defined as a weighted KL divergence,

$${ℒ}_{\text{att}\;}=\frac{1}{\left|{𝒯}_{\text{sup}}\right|}\sum_{t\in {𝒯}_{\text{sup}\;}}\sum_{i,j}log\left(1+A_{\text{raw},ij}^{\left(t\right)}\right){\tilde{A}}_{ij}^{\left(t\right)}log\frac{{\tilde{A}}_{ij}^{\left(t\right)}}{{\tilde{\overset{ˆ}{A}}}_{ij}^{\left(t\right)}},$$

where $A_{\text{raw}}^{\left(t\right)}$ denotes the unnormalised interaction intensity used to construct $A^{\left(t\right)}$. For training pairs without interaction annotations, only ${ℒ}_{\text{cls}}$ is applied.

The final objective is

$$ℒ=(1-\lambda ){ℒ}_{cls}+\lambda {ℒ}_{att},$$

where λ∈[0,1] controls the contribution of attention supervision. In practice, λ is selected based on validation performance via an ablation study, and we set λ=0.3 for all experiments, which achieves the best overall trade-off between predictive accuracy and attention alignment (see Supplementary Section S.2.7).

## Supplementary Material

Supplement 1

## Figures and Tables

**Figure 5:**
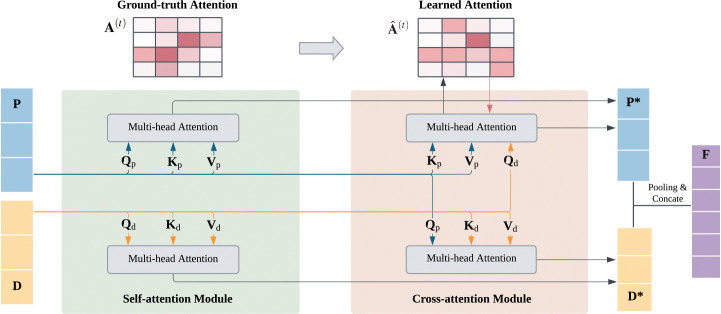
Interaction Module: Token-level embeddings of proteins (**P**) and ligands (**D**) are refined by self-attention and aligned by cross-attention. Multi-head attention yields per-head interaction maps {${\overset{ˆ}{A}}^{\left(t\right)}$}, which can be supervised against ground-truth maps {**A**^(t)^} when available. Fused embeddings P* and D* are mean-pooled and concatenated into **F**.

**Table 1:** Performance of ExplainBind and baselines under the in-distribution (ID) setting.

Model	AUC	AUPR	Accuracy	F1	Sensitivity	Specificity	MCC

SVM	0.939±0.004	0.927±0.005	0.824±0.006	0.820±0.007	0.811±0.009	0.841±0.010	0.701±0.012
RF	0.941±0.006	0.922±0.007	0.879±0.008	0.874±0.009	0.869±0.010	0.889±0.011	0.814±0.013
DeepConv-DTI	0.946±0.003	0.926±0.004	0.883±0.006	0.879±0.007	0.871±0.008	0.886±0.009	0.819±0.010
GraphDTA	0.952±0.002	0.935±0.005	0.889±0.004	0.885±0.014	0.881±0.006	0.891±0.006	0.826±0.007
MolTrans	0.951±0.022	0.937±0.012	0.886±0.004	0.881±0.005	0.874±0.010	0.891±0.007	0.821±0.008
DrugBAN	0.976±0.012	0.943±0.004	0.893±0.004	0.883±0.014	0.881±0.005	0.896±0.006	0.829±0.006
LANTERN	0.982±0.003	0.942±0.003	0.901±0.024	0.879±0.036	0.889±0.023	0.887±0.035	0.839±0.029
GraphBAN	0.978±0.002	0.944±0.003	0.889±0.004	0.885±0.004	0.899±0.005	0.907±0.005	0.841±0.006

**ExplainBind**	**0.993±0.003**	**0.954±0.002**	**0.912±0.011**	**0.905±0.006**	**0.900±0.004**	**0.915±0.004**	**0.857±0.005**

The **best** results are shown in bold, and the second best results are underlined.

## Data Availability

The InteractBind database is derived from publicly available protein–ligand complex structures deposited in the Protein Data Bank (PDB). **InteractBind has been uploaded to the manuscript submission system for peer review**. The source code required to reproduce the results of this study is publicly available at **GitHub**: https://github.com/ZhaohanM/ExplainBind. A user-friendly web interface is accessible via the **Demo UI**: https://huggingface.co/spaces/Zhaohan-Meng/ExplainBind.
