# ExplainBind: Explainable Physicochemical Determinants of Protein–Ligand Binding via Non-Covalent Interactions

**DOI:** 10.64898/2026.03.03.707476

**Published:** 2026-06-09

**Authors:** Zhaohan Meng, Zhen Bai, Ke Yuan, Jiangning Song, Yumeng Zhang, Jaime H. Cheah, Wei Jiang, Adam Skepner, Kevin J. Leahy, Iadh Ounis, William M. Oldham, Zaiqiao Meng, Hao Xu, Joseph Loscalzo

**Affiliations:** 1 School of Computing Science, University of Glasgow; 2 School of Life Science and Technology, Institute of Science Tokyo; 3 School of Cancer Sciences, University of Glasgow; 4 Cancer Research UK Scotland Institute; 5 Monash Biomedicine Discovery Institute and Department of Biochemistry and Molecular Biology, Monash University; 6 Center for the Development of Therapeutics, The Broad Institute of MIT and Harvard; 7 Department of Medicine, Brigham and Women’s Hospital, Harvard Medical School, Harvard University; 8 Department of Medicine, The Warren Alpert Medical School of Brown University; 9 The Broad Institute of MIT and Harvard; 10 Language Technology Lab, University of Cambridge

## Abstract

Protein-ligand binding governs enzymatic catalysis, metabolic homeostasis, and therapeutic modulation. Thus, the accurate prediction of these interactions underpins modern rational drug discovery. However, existing deep-learning frameworks largely operate as black-box predictors that fail to resolve the individual residues mediating binding or decode the fundamental non-covalent forces that drive molecular recognition. To address these limitations, we present **ExplainBind**, an interaction-aware framework that predicts binding likelihood, localizes specific binding residues at single-amino-acid resolution rather than coarse pocket-level regions, and decodes the underlying non-covalent interaction patterns, all zero-shot, without requiring prior three-dimensional structural inputs. To support residue- and interaction-level training and evaluation, we construct **InteractBind**, a protein–ligand benchmark with residue–atom interaction maps. Simultaneously, benchmarking experiments demonstrate that ExplainBind consistently outperforms state-of-the-art baselines across diverse protein and ligand spaces, maintaining high precision when generalized to entirely novel sequences and chemical scaffolds. When applied to two unseen therapeutic targets, ExplainBind successfully ranks potent angiotensin-converting enzyme (ACE) inhibitors and clarifies differences in their potency via affinity-stratified interaction landscapes. Furthermore, we demonstrate the prospective utility of ExplainBind by discovering novel inhibitors and activators of L-2-hydroxyglutarate dehydrogenase (L2HGDH) through wet-lab validation, with mechanistically distinct interaction profiles providing a clear molecular rationale for their divergent functional outcomes. Collectively, these results establish ExplainBind as a powerful, generalizable tool for mechanistically informed, interpretable drug discovery.

## 1 Introduction

The machinery of life is orchestrated through intricate networks of molecular interactions [1, 2]. Enzymes accelerate chemical reactions by stabilizing transition states, signaling proteins transmit information through transient contacts, and therapeutics achieve efficacy by engaging precise binding sites [3]. Underlying these processes is a constellation of non-covalent forces: hydrogen bonds, salt bridges, van der Waals contacts, hydrophobic contacts, π–π stacking, and cation–π interactions that collectively dictate affinity, specificity, and kinetics [4]. Acting in concert, these interactions stabilize enzymatic catalysis, organize macromolecular assemblies, and confer selectivity to drugs [5]. Crucially, they are exquisitely sensitive to select structural determinants: the loss of a single hydrogen bond, the reorientation of an aromatic ring, or the disruption of a hydrophobic patch can shift a system from active to inert, or from selective to promiscuous. Non-covalent interactions are, therefore, not incidental features, but the fundamental determinants of molecular recognition across biology and medicine [6].

Capturing this physical precision has long demanded three-dimensional structures, where atomic geometries directly encode the non-covalent forces governing molecular recognition. Classical physics-based approaches, molecular docking [7] and molecular dynamics simulations [8], model interaction energies directly from atomic coordinates to rationalize binding and specificity. More recently, geometric deep learning has extended this paradigm to data-driven settings: MaSIF [9, 10] learns molecular surface fingerprints at protein–protein interfaces, and related ideas have since given rise to distance-aware geometric models for protein–ligand docking [11–14]. Yet, in these approaches, distance-based geometry serves only as an implicit proxy for molecular interactions, the underlying non-covalent interactions are never directly represented. This limitation runs deeper than it first appears: recent adversarial analyses of representative protein–ligand co-folding models, including AF3 [15], RoseTTAFold All-Atom [16], Chai-1 [17] and Boltz-1 [18], reveal that high structural accuracy does not guarantee physically meaningful interactions — systematic violations of steric and electrostatic principles persist even at low root-mean-square deviation (RMSD), exposing a fundamental discordance between geometric fidelity and chemical realism [19].

This gap is not confined to structure-based models but extends equally to sequence-based approaches [20–24], where the field has converged on protein–ligand binding (PLB) prediction as a scalable surrogate objective [25, 26]. Sequence-based PLB models typically employ cross-attention or related fusion modules and claim to infer latent residue–atom correspondences [27–29], yet, whether these attention patterns reflect true physical interactions has never been systematically evaluated. A fundamental question therefore remains unanswered: do learned attention representations capture the non-covalent forces that actually govern binding, or merely correlate with binding likelihood?

**Figure 1:**
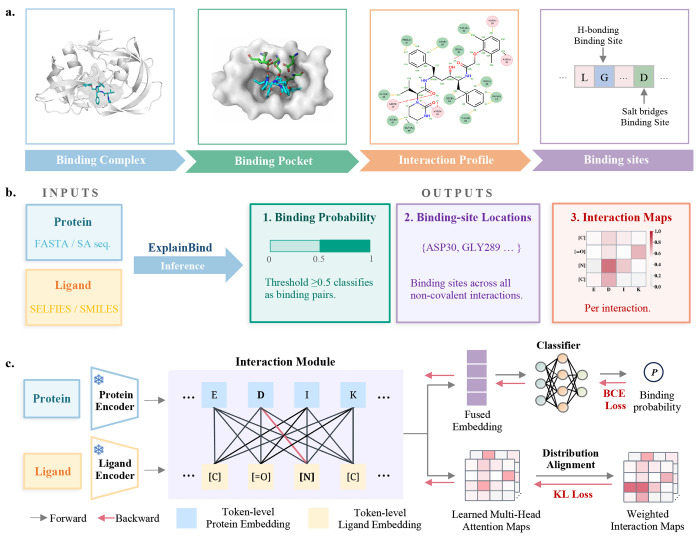
Multi-scale protein–ligand binding representation and ExplainBind framework. **a.** Multi-scale representation of protein–ligand binding: binding complex, binding pocket, interaction profile, and interaction-type–specific binding sites. **b.** During inference, ExplainBind takes a protein sequence and ligand representation as input and predicts binding probability, localizes binding-site residues, and generates interaction maps across six non-covalent interaction types. **c.** During training, frozen protein and ligand encoders produce token-level embeddings fused by an interaction module, with a BCE loss supervising binding prediction and a weighted KL divergence loss grounding multi-head attention maps in ground-truth interaction maps from InteractBind.

To address this challenge, here we present **ExplainBind**, a protein–ligand binding framework that jointly predicts binding likelihood, localizes binding sites at the residue level, and decodes the underlying non-covalent interaction patterns from sequence alone, without requiring three-dimensional structural inputs. Binding can be represented at multiple scales: from the full binding complex and pocket to a residue-level interaction profile and finally to interaction-type–specific binding sites (Fig. 1a). To enable training and evaluation at the residue and interaction-level, we construct **InteractBind**, a curated protein–ligand benchmark derived from experimentally resolved complexes and annotated with residue–atom interaction maps. By explicitly supervising its cross-attention module with physically grounded interaction maps, ExplainBind bridges predictive accuracy and mechanistic interpretability, moving beyond the binary binding classifiers that currently dominate the field (Fig. 1b,c). Evaluated across a controlled similarity spectrum from closely related to deliberately dissimilar protein and ligand sequences, ExplainBind achieves state-of-the-art performance while its learned attention maps reliably predict binding-site localization. Applied retrospectively to angiotensin-converting enzyme (ACE) to enrich potent inhibitors, and prospectively to L-2-hydroxyglutarate dehydrogenase (L2HGDH) to distinguish inhibitors from activators, the framework demonstrates broad utility for mechanistically informed drug discovery and enzyme engineering.

## 2. Results

### 2.1 Overview of ExplainBind

ExplainBind is an end-to-end framework that connects protein–ligand binding prediction with binding-site localization and mechanistic modeling of the physicochemical interactions that mediate molecular recognition. As shown in Fig. 1c, the framework uses frozen foundation encoders with an interaction module to capture token-level interactions between amino acid residues and ligand atoms, supervised using interaction-type–specific maps derived from curated protein–ligand complexes across six non-covalent interactions. Our proposed framework is jointly optimized with two complementary objectives: a Binary Cross-Entropy (BCE) loss [30] on the predicted binding probability and a Kullback–Leibler divergence (KL) loss [31] aligning the learned attention maps with ground-truth non-covalent interaction maps, grounding predictions in biologically meaningful residue–atom relationships.

Underpinning this framework is **InteractBind**, a novel large-scale database that we constructed by systematically mining and annotating protein–ligand complexes from the Protein Data Bank (PDB) [32]. We localize the binding pocket from each complex and then extract non-covalent interaction annotations. Non-covalent interactions are identified using rule-based criteria grounded in geometric and chemical constraints, with interaction-specific distance cutoffs and optional angular filters applied to ensure validity (Supplementary Table S1). Interaction strength is modelled by a distance-dependent piecewise linear decay function that maps interatomic or geometric distances within the cutoff window to continuous values in [10^−6^, 1], yielding an *m* × *n* interaction map per interaction type, where *m* and *n* denote the numbers of ligand units and protein residues, respectively. In addition, The six non-covalent interaction maps are aggregated into an overall interaction map. Finally, residue-level binding-site labels are obtained by projecting the overall interaction map onto the residue dimension.

We evaluate ExplainBind and seven representative baselines (Supplementary S.2.4) under both in-distribution (**ID**) and out-of-distribution (**OOD**) settings. For ID, we use the affinity-aware subset from InteractBind, stratifying pairs according to binding affinity (kcal/mol) and retaining only high-confidence negative samples (≥ −5 kcal/mol) and positive samples (≤ −7 kcal/mol) for supervision, whilst excluding cases of moderate affinity to reduce label ambiguity. For the OOD setting, we construct similarity-controlled evaluation scenarios along two complementary axes. Ligand chemical similarity is quantified using Tanimoto similarity over extended connectivity fingerprints (ECFP) [33], yielding mean train–test similarities of 8%, 35%, 40%, and 59%, with protein similarity fixed at 33% across all splits. Protein similarity is quantified using global sequence alignment based on the Needleman–Wunsch algorithm [34], yielding peak train–test similarities of 25%, 28%, 31%, and 33%. Combining these regimes results in eight OOD datasets spanning progressively increasing degrees of chemical and sequence novelty. For binary binding prediction, model performance is assessed using accuracy (Acc), area under the receiver operating characteristic curve (AUROC), area under the precision–recall curve (AUPRC), and F1 score. For binding-site localization, we report Binding Residue Hit Rate (BRHR) and Recall, whereas for non-covalent interaction prediction, we report Interaction Hit Rate (IHR) and Recall, following the evaluation protocol defined in Supplementary S.2.3.

### 2.2 From binary binding prediction to binding-site localization

We evaluated ExplainBind and seven representative baselines on both binary binding prediction and binding-site localization, asking whether the strong classification performance characteristic of current sequence-based methods translates into accurate identification of the non-covalent contacts that mediate protein–ligand recognition. The answer is an unambiguous no. Across seven representative baselines spanning from cross-attention architectures (MolTrans [35], TransformerCPI [20], CAT-DTI [27], HyperAttentionDTI [21], PerceiverCPI [36]) to bilinear attention mechanisms (DrugBAN [28], GraphBAN [37]), binary binding prediction approaches saturation under in-distribution conditions, with AUROC ranging from 95.1% to 97.8% and accuracy from 84.1% to 89.3% (Table 1). Yet, binding-site localization remains uniformly and severely limited: BRHR@1 ranges from only 10.6% to 16.1% across all baselines, meaning that even the strongest model fails to recover binding residues in its top prediction for more than 83% of protein–ligand pairs.

Critically, high binary classification performance provides no reliable indication of localization ability. PerceiverCPI achieves a strong AUROC of 96.9% but the lowest BRHR@1 of 10.6%, while GraphBAN obtains the strongest baseline AUROC and F1 yet is surpassed by DrugBAN on BRHR@3, BRHR@5, and Recall@5. These crossovers suggest that binding prediction and binding-site localization remain decoupled under binding label-only supervision. By explicitly supervising cross-attention with physically grounded, interaction-type-specific maps, ExplainBind achieves 55.6% BRHR@1, 69.5% BRHR@3, and 74.6% BRHR@5, improving over the strongest baselines by 39.5, 45.6, and 43.2 percentage points, respectively. It also improves Recall@5 and Recall@10 to 7.3% and 8.7%, while achieving the best binary prediction performance across all metrics: 91.2% accuracy, 95.4% AUPRC, 99.3% AUROC, and 90.5% F1. Additional ablation studies analysing the contribution of each component are provided in Supplementary S.3.8.

### 2.3 Generalization under similarity-controlled evaluation

**Figure 2:**
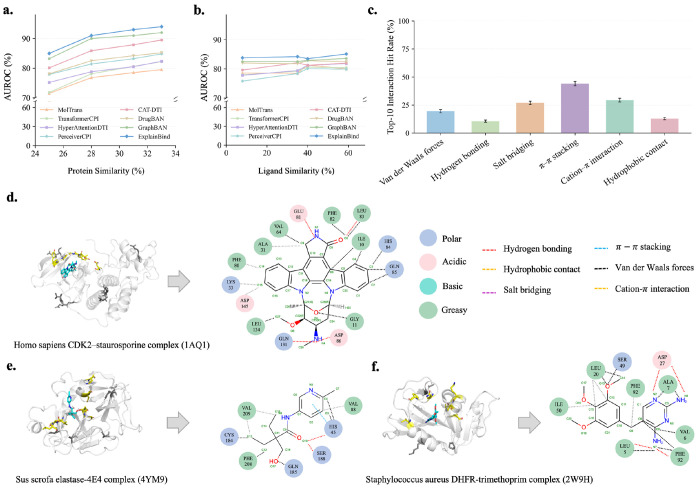
Out-of-distribution binding prediction and non-covalent interaction prediction via ExplainBind. **a.** OOD performance under protein sequence similarity–controlled splits, with AUROC increasing consistently as sequence similarity increases. **b.** OOD performance under ligand similarity–controlled splits, showing stable AUROC across ligand-similarity thresholds. **c.** Interaction-specific non-covalent interaction prediction measured by Top-10 Interaction Hit Rate (IHR@10) across non-covalent interaction types. **d-f.** Case studies of representative protein–ligand complexes (PDB: 1AQ1, 4YM9 and 2W9H). Interaction maps derived from crystal structures are compared with model predictions. The top 20 predicted residues recover all experimentally observed contacts. Ligands are shown in cyan, the top 1 predicted residue is highlighted in yellow and lies within the experimental binding site, whereas lower-ranked residues among the top 20 that fall outside the annotated site are shown in black.

Generalization of sequence-based binding models is further evaluated under carefully controlled out-of-distribution settings based on protein and ligand similarity. In the protein-controlled setting, test proteins are constrained to have limited sequence similarity to training proteins, modeling generalization to unseen target families (Fig. 2a). Across models, performance decreased as the train–test sequence-similarity threshold became more stringent, indicating that protein sequence divergence is a major source of distribution shift. This trend suggests that conventional in-distribution evaluation can overestimate performance when sequence-based binding models are applied to novel protein targets. Despite this challenging setting, ExplainBind achieves the strongest performance, with an AUROC of 85 at the most stringent 25% threshold and 94 at 33%, showing greater robustness to target-side novelty than existing baselines.

For ligand-controlled splits, test ligands are constrained to be chemically dissimilar from the training ligands (Fig. 2b). In contrast to the protein-controlled setting, all models show a narrower performance range across ligand similarity thresholds, suggesting that chemical diversity among ligands produces a comparatively modest distribution shift when protein targets are sufficiently represented. ExplainBind maintains stable performance, with AUROC values ranging from 83 to 85 across ligand similarity thresholds from 8% to 59%, and remains consistently competitive with the baselines. Together, these results show that target-side novelty is the dominant challenge for sequence-based binding prediction, while ExplainBind provides stronger OOD generalization across protein- and ligand-controlled screening scenarios.

### 2.4 Prediction of non-covalent interactions

Beyond binding-site localization, ExplainBind resolves the specific non-covalent forces mediating each residue–atom contact, producing six interaction-type-specific maps for each protein–ligand pair (Fig. 2c). To evaluate its performance, we apply a strict exact-match criterion using the Top-10 Interaction Hit Rate (IHR@10), where at least one of the ten highest-scoring residue–atom pairs must match a true contact among approximately 12,880 candidates (368 protein residues × 35 ligand atoms), of which fewer than three are annotated contacts on average, giving a random success rate of approximately 0.23%. Despite this stringency, ExplainBind recovers ground-truth contacts across all six interaction types. π–π stacking achieves the highest IHR@10 (44.2%), consistent with the distinctive aromatic residue signatures that mediate this interaction. Cation–π interactions and salt bridges also show strong performance, reaching 29.4% and 27.0% , respectively, reflecting the chemically constrained residue preferences associated with charged and aromatic contacts. Van der Waals forces achieve 19.5%, whereas hydrophobic contacts (12.9%) and hydrogen bonding (10.6%) are more challenging, likely because they involve broader residue and atom types and are less defined by simple sequence-level chemical signatures. Detailed interaction-specific IHR@*K* and Recall@*K* results, together with their analysis, are provided in Supplementary S.3.1.

### 2.5 Case studies of non-covalent interaction recognition

We examined three co-crystallized protein–ligand complexes from distinct biological contexts to assess whether ExplainBind’s predicted interaction maps agree with crystallographically resolved contacts: *Homo sapiens* CDK2–staurosporine, *Sus scrofa* elastase–4E4, and *Staphylococcus aureus* DHFR–trimethoprim (Fig. 2d–f). For CDK2–staurosporine, ExplainBind recovered hydrogen-bonding residues including GLU81, LEU83, ASP86, and GLN131, alongside hydrophobic and van der Waals contacts that stabilize the ligand within the kinase binding cleft. For elastase–4E4, the model identified hydrogen bonds near the catalytic triad, hydrophobic contacts around VAL88, VAL209, and PHE208, and a π–π stacking interaction involving HIS45. For DHFR–trimethoprim, ExplainBind captured the mixed polar–apolar active-site environment, including hydrogen bonds at LEU5, ASP27, and PHE92, and hydrophobic contacts at ILE50, LEU20, and PHE92. Across all three complexes, the predicted interaction patterns agreed with crystallographically observed contacts, demonstrating that ExplainBind recovers chemically interpretable recognition features without structural input. Such outputs provide experimentally actionable hypotheses for identifying ligand-recognition residues, prioritizing mutagenesis, and guiding mechanism-aware ligand optimization (Supplementary S.3.2).

### 2.6 Prioritization of high-activity ligands

To evaluate ExplainBind under a ligand prioritization setting, we performed retrospective enrichment analysis on angiotensin-converting enzyme (ACE), a key regulator of blood pressure and cardiovascular homeostasis [38], and wet-lab validation on L-2-hydroxyglutarate dehydrogenase (L2HGDH), a mitochondrial metabolic enzyme for which both activation and inhibition are of potential therapeutic interest [39]. Neither target appears in the InteractBind training set or has a closely related homolog in the training data, providing a stringent evaluation on genuinely unseen proteins.

#### Retrospective prioritization on ACE.

**Figure 3:**
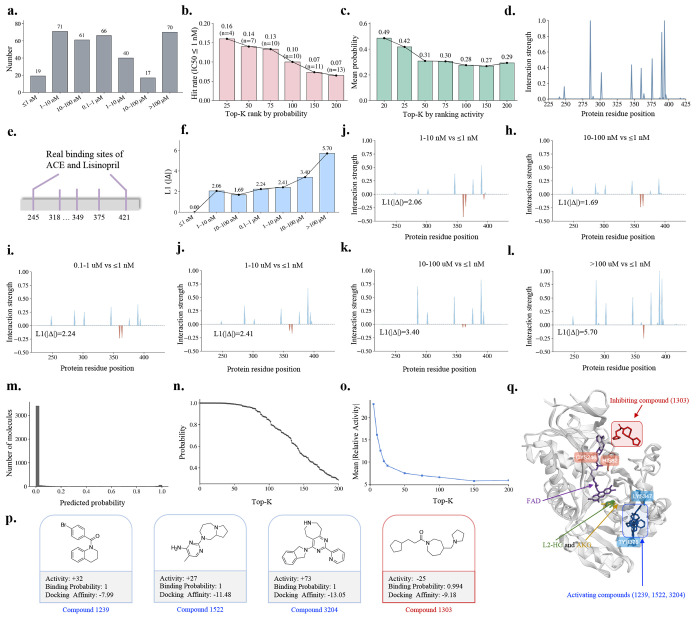
Ligand prioritization for ACE and L2HGDH. **a.** Distribution of ACE ligands across IC_50_ affinity strata. **b.** Enrichment of highly potent compounds (IC_50_ ≤ 1 nM) among the Top-*K* candidates ranked by predicted binding probability. **c.** Mean predicted binding probability across Top-*K* candidates ranked by experimental activity. **d.** Predicted ACE binding-site interaction landscape. **e.** Experimentally resolved ACE–lisinopril binding sites. **f.** Overall shift in predicted interaction patterns, measured by *ℓ*_1_ (|Δ|), between each lower-affinity stratum and the IC_50_ ≤ 1 nM reference group. **g–l.** Position-wise interaction changes (Δ) across affinity strata relative to the IC_50_ ≤ 1 nM group, with positive and negative values denoting enhanced and reduced predicted interactions, respectively. **m.** Distribution of predicted binding probabilities across the internal compound pool (3,750 compounds) with L2HGDH, broadly consistent with the experimentally observed activity spectrum. **n.** The binding probability ranking exhibits a rapid decay from 1 to 0.3 for the top 200 compounds with L2HGDH. **o.** The mean |Relative Activity| within the top 200 compounds indicates a significant enrichment of active compounds among the highly ranked candidates. **p.** The highly active ligands within the Top-50 compounds, illustrating scaffold diversity among both experimentally validated inhibitors and activators. **q.** Mechanistic basis of L2HGDH modulation by representative ligands. Docked poses of activators (compounds 1239, 1522, and 3204; blue) and inhibitor (compound 1303; red) superimposed on the L2HGDH structure (PDB: 8W78, chain A; semi-transparent surface), as determined by global docking. The natural substrate L-2-hydroxyglutarate (L2-HG; green), product *α*-ketoglutarate (AKG; yellow), and FAD cofactor (purple) are shown for reference. ExplainBind-predicted hydrogen-bonding interactions reveal mechanistically distinct binding modes: inhibitor engagement is mediated by Cys246 and His82, residues situated in the vicinity of the FAD-binding region, suggesting that inhibitor occupancy at this site may sterically compress the FAD cofactor deeper into the catalytic pocket, occluding substrate entry and thereby abolishing catalytic activity. In contrast, activator binding is coordinated through Lys347 and Tyr376, which are spatially distinct from the FAD pocket.

As shown in Fig. 3a, ACE ligands span a broad range of IC_50_ affinity strata. When ranked by predicted binding probability, ultra-potent ligands (IC_50_ ≤ 1 nM) are preferentially enriched among the top-ranked candidates, with enrichment decreasing smoothly as the ranking cutoff Top-*K* increases from 25 to 200 (Fig. 3b). The Top-25 ranked compounds achieve a hit rate of 0.16, demonstrating that highly potent ligands are preferentially concentrated among top-ranked candidates. Consistent with this trend, mean predicted binding probability decreases with increasing Top-*K* when compounds are ordered by experimental activity, decreasing from 0.486 at Top-20 to approximately 0.293 at Top-200 (Fig. 3c).

Beyond compound-level ranking, ExplainBind provides interaction profiles that reveal affinity-dependent patterns of molecular recognition. The predicted ACE interaction landscape is broadly consistent with experimentally resolved ACE–lisinopril binding sites [40] (Fig. 3d,e). Comparing interaction patterns across progressively weaker affinity strata relative to the IC_50_ ≤ 1 nM reference group (Fig. 3f–l), interaction differences remain localised and modest at nanomolar potency (*ℓ*_1_ norms: 1.687–2.236), but become increasingly pronounced as affinity decreases into the micromolar regime (*ℓ*_1_ norm: 3.400 for 10–100 μ*M*; 5.703 for IC_50_ > 100 *μ*M), indicating systematic erosion of key interaction patterns with reduced binding potency.

#### Wet-lab validation on L2HGDH.

ExplainBind is applied to an internal pool of 3,750 compounds to predict binding probability and guide experimental selection for this mitochondrial enzyme active in central carbon metabolism [41]. The majority of compounds are assigned low predicted probabilities below 0.2 (Fig. 3m), with the Top-200 spanning from 1.0 to approximately 0.3 (Fig. 3n), motivating their selection for experimental testing, where relative activity was measured as the percent change in enzyme activity relative to vehicle control (Supplementary S.2.7). Among experimentally tested compounds, the Top-5 ranked achieve a mean |Relative Activity| of 22.40, which decreases rapidly to 9.65 at Top-20 and reaches a shallow tail of approximately 5–7 by Top-200 (Fig. 3o), consistent with progressive inclusion of weaker modulators and a coherent transition from high-confidence, high-activity candidates to lower-confidence predictions.

Experimental measurements confirm strong enrichment of functional modulators among top-ranked compounds. Among the Top-50, four compounds exhibit high experimental activity (|Relative Activity| ≥ 25), including three activators and one inhibitor (Fig. 3p), all assigned high predicted binding probabilities. Global docking of these four compounds against L2HGDH (PDB: 8W78, chain A) is performed to characterize their binding poses, and as these ligands are potent hydrogen-bond acceptors, ExplainBind-predicted hydrogen-bonding interactions are further interrogated to elucidate their mechanistically distinct binding modes: inhibitor engagement (compound 1303) is mediated by Cys246 and His82, residues situated in the vicinity of the FAD-binding region, suggesting that inhibitor occupancy at this site sterically compresses the FAD cofactor deeper into the catalytic pocket, occluding substrate entry and thereby abolishing catalytic activity; whereas activator binding (compounds 1239, 1522, and 3204) is coordinated through Lys347 and Tyr376, residues spatially distinct from the FAD pocket, potentially contributing to an allosteric mechanism of activation (Fig. 3q).

## 3 Discussion

Mechanistic insight into molecular recognition has historically been considered inseparable from three-dimensional structural data—an assumption that has long restricted sequence-based approaches to binary binding predictions of whether binding occurs, leaving the spatial (“where”) and mechanistic (“how”) dimensions of interaction entirely unresolved. ExplainBind fundamentally challenges this structural dogmatism by demonstrating that the essential physical determinants of molecular recognition are recoverable from sequence, provided that the underlying deep-learning architecture is conditioned not merely on binding labels but also on the explicit non-covalent interaction topologies that physically mediate the binding event. The indispensable catalyst for this predictive capacity is not explicit atomic geometry, but rigorous biophysical grounding.

Consequently, our most important finding is not simply that ExplainBind predicts binding more accurately, but that it resolves where and how binding occurs with high resolution—a distinction that yields immediate translational utililty. Delineating the exact residues that anchor a ligand and through which non-covalent forces establishes a useful framework rational mutagenesis, enables precise selectivity engineering, and constrains the chemical space of productive modifications in ways that a monolithic binding probability alone cannot. For ACE, this translates into affinity-stratified interaction landscapes that systematically identify which contacts are dispensable as binding potency decays. This provides directly actionable structural insights lead optimization in the complete absence of empirical or modeled 3D structural data. For L2HGDH, a target entirely absent from training, ExplainBind successfully differentiated inhibitors from activators through differential interaction profiles anchored to mechanistically distinct residues: inhibitor engagement was localized in close proximity to the FAD-binding region, strongly implying a mechanism of competitive steric occlusion that blocks substrate entry. Conversely, activator coordination was mapped to spatially segregated residues, pointing toward a positive allosteric modulation mechanism—a resolution fundamentally inaccessible to conventional models trained on binary binding labels alone. That this precise discrimination was achieved prospectively, on a genuinely unseen target, underscores that ExplainBind’s interaction profiles carry functional predictive power that extends far beyond simple binding likelihoods, offering profound implications for context-dependent therapeutic targeting of metabolic enzymes.

Sequence-based protein–ligand models have increasingly adopted cross-attention mechanisms with the implicit expectation that meaningful residue–atom correspondences would spontaneously from binding label supervision alone. Whether the resulting attention patterns reflect the actual non-covalent forces that govern molecular recognition, however, has never been systematically tested. ExplainBind addresses this challenge directly, showing that attention maps grounded in experimentally derived interaction profiles successfully recover contact patterns that mirror crystallographically resolved binding interfaces across chemically and phylogenetically diverse complexes. This finding sharply distinguishes the ExplainBind from end-to-end structure-based co-folding models such as AF3, RoseTTAFold All-Atom, Chai-1 and Boltz-1. While these deep-learning co-folding models generate plausible binding geometries, they frequently yield local coordinates that violate fundamental steric and electrostatic constraints, resulting in atomic clashes or unphysical ligand distortions [19]. This divergence suggests that geometric fidelity and chemical realism are dissociable, and that capturing the latter mandates explicit physicochemical conditioning and supervision. More broadly, any model trained solely on binary binding outcomes will inevitably converge on statistical correlates of binding rather than its causal physical drivers. This critical distinction becomes the limiting bottleneck precisely when translating a model’s utility from retrospective classification tasks to prospective, mechanistically sound hypothesis generation.

The convergence of scalable, sequence-based inference with mechanistic transparency positions ExplainBind as a versatile, broadly applicable tool for contexts where structural data are absent, low-resolution, or entirely unresolvable. These applications range from high-throughput ligand prioritization across understudied protein families to the rational engineering of industrial or therapeutic enzymes with reprogrammed substrate specificities. Looking forward, extending interaction-grounded supervision to multi-target selectivity profiling and integration with generative molecular design frameworks, where interaction-type constraints could guide scaffold optimization toward desired binding modes, represents a natural and immediate direction. As experimentally annotated interaction data continue to expand through structural genomics and screening efforts, frameworks that ground and anchor learned representations in foundational physical interaction principles will become increasingly central to predictive molecular biology.

## 4 Methods

### 4.1 Problem Formulation

We formulate protein–ligand binding (PLB) modeling as a joint learning problem over three complementary tasks: (1) binary binding prediction, (2) binding-site localization, and (3) non-covalent interaction prediction. These tasks share the same protein–ligand input but differ in their prediction objectives.

#### Binary binding prediction.

Given a protein sequence $𝒫=\left(p_{1},\ldots ,p_{n}\right)$ composed of residue-level tokens and a ligand sequence $𝒟=\left(d_{1},\ldots ,d_{m}\right)$ representing atom-level structure, the objective of binary binding prediction is to determine whether the protein–ligand pair forms a binding interaction. Formally, this task can be written as

$$f^{\text{bind}}:(𝒫,𝒟)⟶p,$$

where *p* ∈ [0, 1] is a scalar probability indicating the likelihood that the protein–ligand pair interacts.

#### Binding-site prediction.

In addition to binary binding prediction, PLB modeling requires localization of the protein residues and ligand atoms involved in binding. Given the same protein sequence 𝒫 and ligand sequence 𝒟, the objective is to identify the set of protein residues and ligand atoms that participate in the binding mechanism. Formally, this task is defined as

$$f^{\text{site}}:(𝒫,𝒟)⟶{𝒮}^{\text{site}},$$

where ${𝒮}^{\text{site}}=\left\{\left(d_{i},p_{j}\right)\right\}$ denotes the predicted list of ligand-atom and protein-residue pairs involved in binding. Each pair $\left(d_{i},p_{j}\right)\in {𝒮}^{\text{site}}$ indicates that ligand atom $d_{i}$ and protein residue $p_{j}$ are predicted to participate in the binding.

#### Non-covalent interaction prediction.

Beyond identifying binding-site locations, PLB modeling further requires distinguishing the physicochemical interaction types that mediate protein–ligand recognition. Given the same protein sequence 𝒫 and ligand sequence 𝒟, the objective is to predict a set of interaction-specific residue–atom maps, each corresponding to a particular class of non-covalent interaction. Formally, this task is defined as

$$f^{\text{type}}:(𝒫,𝒟)⟶{\left\{A^{\left(t\right)}\right\}}_{t=1}^{T},$$

where T denotes the number of non-covalent interaction types. Each matrix $A^{\left(t\right)}\in R^{m\times n}$ represents the residue-atom interaction pattern associated with non-covalent interaction type t, and each entry $A_{ij}^{\left(t\right)}$ indicates the predicted likelihood that ligand atom $d_{i}$ and protein residue $p_{j}$ form interaction type t.

Together, these tasks characterize protein–ligand binding at increasing levels of resolution: whether binding occurs, where the binding occurs, and which physicochemical interactions mediate recognition.

### 4.2 Model Architecture

#### 4.2.1 Encoders

Capturing rich and biologically grounded representations is essential for exploring fine-grained and explainable protein–ligand interactions. For ligands, SMILES strings are widely used but may contain invalid segments and lose critical structural cues [42]. To overcome these limitations, we adopt SELFIES [42], a robust molecular grammar that guarantees valid molecular graphs and preserves atom-level information [42]. For proteins, we primarily use FASTA sequences, in which each token corresponds to one of the 23 standard amino acid symbols. When reliable structural information is available, ExplainBind can optionally incorporate structure-aware (SA) protein representations derived via *Foldseek* [43], providing explicit local structural context [44].

Foundation models have shown strong capability in encoding contextual and structural information through large-scale pretraining on biological corpora. The framework combines frozen foundation encoders for proteins and ligands, enabling ExplainBind to leverage powerful pretrained representations without updating the backbone models. In our default configuration, we use ESM-2 [45] as the frozen protein encoder and SELFormer [46] as the frozen ligand encoder for SELFIES representations. These encoders produce token-level protein embeddings **P** and ligand embeddings **D** that serve as inputs to the interaction module, forming the basis for accurate binding prediction and explainable interaction modeling, including SaProt [44] when SA representations are used, as well as ProteinBERT [47], MoLFormer [48], and ChemBERTa-2 [49], with results summarized in Supplementary S9.

#### 4.2.2 Interaction module

Unlike prior unsupervised models for PLB prediction, ExplainBind explicitly supervises attention with ground-truth maps derived from experimentally resolved protein–ligand complexes. Concretely, we convert residue–level contacts in each complex into per-type interaction maps and use them to directly optimize the attention computed from protein and ligand embeddings. To our knowledge, our proposed framework is the first to use complex-level attention maps to guide token-level fusion of proteins and ligands. Unsupervised attention conflates heterogeneous physical forces and tends to produce diffuse, non-identifiable alignments. By supervising attention with per-type contact maps, we (a) disambiguate interaction mechanisms (e.g., hydrogen bond vs. hydrophobic contact), (b) improve the faithfulness of explanations, and (c) use the same supervision signal to shape the fused token embeddings towards binding-relevant substructures.

Let $D\in R^{m\times h}$ and $P\in R^{n\times h}$ denote the token-level embeddings of the ligand and protein, respectively. Linear projections generate query (**Q**), key (**K**), and value (**V**) matrices for each modality:

$$Q_{d}=DW_{q}^{d},\;K_{d}=DW_{k}^{d},\;V_{d}=DW_{v}^{d},\;Q_{p}=PW_{q}^{p},\;K_{p}=PW_{k}^{p},\;V_{p}=PW_{v}^{p},$$

where $W_{\left(\cdot \right)}^{\left(\cdot \right)}\in R^{h\times h}$ are learnable projections.

The interaction module uses multi-head attention with *H* = 8 heads: six heads correspond to non-covalent interactions, and two heads capture overall binding patterns. For each head t∈𝒯, bidirectional cross-attention computes the residue-level interaction intensity (where h is the hidden dimension size):

$${\hat{A}}_{pd}^{\left(t\right)}=Softmax(\frac{Q_{p}^{\left(t\right)}K_{d}^{(t{)}^{⊤}}}{\sqrt{h_{t}}}),\;{\hat{A}}_{dp}^{\left(t\right)}=Softmax(\frac{Q_{d}^{\left(t\right)}K_{p}^{(t{)}^{⊤}}}{\sqrt{h_{t}}}),$$

where ${\hat{A}}_{pd}^{\left(t\right)}\in R^{n\times m}$ and ${\hat{A}}_{dp}^{\left(t\right)}\in R^{m\times n}$. Self-attention is simultaneously applied to refine intra-sequence contexts:

$$P_{sa}^{\left(t\right)}=Softmax(\frac{Q_{p}^{\left(t\right)}K_{p}^{(t{)}^{⊤}}}{\sqrt{h_{t}}})V_{p}^{\left(t\right)},\;D_{sa}^{\left(t\right)}=Softmax(\frac{Q_{d}^{\left(t\right)}K_{d}^{(t{)}^{⊤}}}{\sqrt{h_{t}}})V_{d}^{\left(t\right)}.$$

Type-specific fused representations are obtained by combining self- and cross-attention outputs:

$$P^{*(t)}=\frac{1}{2}\left[P_{sa}^{\left(t\right)}+{\hat{A}}_{pd}^{\left(t\right)}V_{d}^{\left(t\right)}\right],\;D^{*(t)}=\frac{1}{2}\left[D_{sa}^{\left(t\right)}+{\hat{A}}_{dp}^{\left(t\right)}V_{p}^{\left(t\right)}\right].$$

Finally, outputs from all heads are concatenated and projected to yield the fused token sequences:

$$P^{*}={Concat}_{t\in 𝒯}\left(P^{*(t)}\right)W_{p}^{o},\;D^{*}={Concat}_{t\in 𝒯}\left(D^{*(t)}\right)W_{d}^{o}.$$

#### 4.2.3 Classifier

Given the fused token embeddings $P^{*}\in R^{n\times d}$ and $D^{*}\in R^{m\times d}$, we form a pair-level representation by mean pooling over tokens and concatenating the pooled vectors:

$${\bar{P}}^{*}=\frac{1}{n}\sum_{j=1}^{n}P_{j}^{*},\;{\bar{D}}^{*}=\frac{1}{m}\sum_{i=1}^{m}D_{i}^{*},\;F=[{\bar{D}}^{*},{\bar{P}}^{*}].$$

The binding probability is predicted by a lightweight MLP head, p=MLP(F).

#### 4.2.4 Training objective

Let y∈{0,1} denote the ground-truth binding label and *p* the predicted binding probability. The model is optimized using a binary cross-entropy classification loss,

$${ℒ}_{cls}=-[y\;log\;p+(1-y)\;log\;(1-p)].$$

When interaction supervision is available, we further align the predicted cross-attention maps with ground-truth interaction annotations. For each supervised head $t\in {𝒯}_{\text{sup}}$, the predicted attention ${\hat{A}}^{\left(t\right)}$ and the ground-truth interaction map $A^{\left(t\right)}$ are normalised over residue-atom pairs to form discrete distributions, ${\tilde{\hat{A}}}^{\left(t\right)}$ and ${\tilde{A}}^{\left(t\right)}$, respectively. The attention alignment loss is defined as a weighted KL divergence,

$${ℒ}_{\text{att}}=\frac{1}{\left|{𝒯}_{\text{sup}}\right|}\sum_{t\in {𝒯}_{\text{sup}}}\sum_{i,j}log\;(1+A_{\text{raw},ij}^{\left(t\right)}){\tilde{A}}_{ij}^{\left(t\right)}log\;\frac{{\tilde{A}}_{ij}^{\left(t\right)}}{{\tilde{\hat{A}}}_{ij}^{\left(t\right)}},$$

where $A_{\text{raw}}^{\left(t\right)}$ denotes the unnormalised interaction intensity used to construct $A^{\left(t\right)}$. For training pairs without interaction annotations, only ${ℒ}_{\text{cls}}$ is applied.

The final objective is

$$ℒ=(1-\lambda ){ℒ}_{cls}+\lambda {ℒ}_{att},$$

where λ∈[0,1] controls the contribution of attention supervision. In practice, λ is selected based on validation performance via an ablation study, and we set λ = 0.3 for all experiments, which achieves the best overall trade-off between predictive accuracy and attention alignment (see Supplementary S.3.8).

## Supplementary Material

1

## Figures and Tables

**Figure 4:**
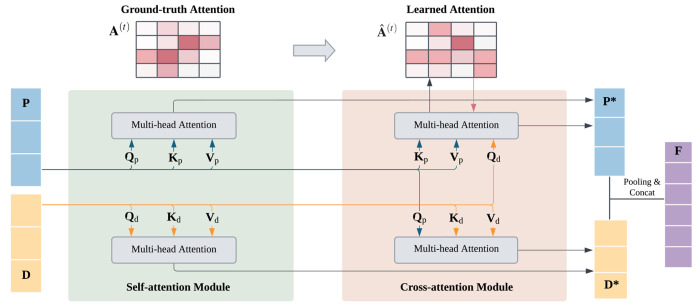
Interaction Module: Token-level embeddings of proteins (**P**) and ligands (**D**) are refined by self-attention and aligned by cross-attention. Multi-head attention yields per-head interaction maps $\left\{{\hat{A}}^{\left(t\right)}\right\}$, which can be supervised against ground-truth maps $\left\{A^{\left(t\right)}\right\}$ when available. Fused embeddings P* and D* are mean-pooled and concatenated into **F**.

**Table 1:** Performance (%) comparison with baselines under the in-distribution setting. Results are mean ± standard deviation over 5-fold cross-validation. BRHR@*K* measures whether at least one of the Top-*K* highlighted protein residues matches a ground-truth binding residue. The best results are shown in **bold**, and the second-best results are underlined.

Model	Binary Binding Prediction				Binding-site Localization				
	ACC	AUPRC	AUROC	F1	BRHR@1	BRHR@3	BRHR@5	Recall@5	Recall@10
MolTrans	88.6 ± 0.7	93.7 ± 1.2	95.1 ± 2.2	88.1 ± 0.5	10.9 ± 0.5	17.7 ± 0.2	22.4 ± 0.8	1.7 ± 0.4	3.5 ± 0.3

TransformerCPI	89.1 ± 0.6	90.8 ± 0.8	95.4 ± 0.3	88.5 ± 0.6	10.9 ± 0.2	16.7 ± 0.7	21.7 ± 0.4	1.7 ± 0.3	3.4 ± 0.4

HyperAttentionDTI	84.1 ± 0.9	92.8 ± 0.3	95.5 ± 0.6	88.5 ± 0.5	12.3 ± 0.4	17.7 ± 0.6	20.8 ± 0.3	1.8 ± 0.3	3.9 ± 0.2

PerceiverCPI	86.6 ± 0.8	92.6 ± 0.5	96.9 ± 0.2	82.2 ± 0.7	10.6 ± 0.1	13.9 ± 0.5	19.9 ± 0.9	1.6 ± 0.2	3.1 ± 0.2

CAT-DTI	88.1 ± 0.5	92.5 ± 0.6	96.8 ± 0.4	87.0 ± 0.3	12.2 ± 0.7	14.8 ± 0.2	22.1 ± 0.6	1.2 ± 0.2	2.8 ± 0.3

DrugBAN	89.3 ± 0.4	94.3 ± 0.4	97.6 ± 1.2	87.5 ± 1.4	15.7 ± 0.6	23.9 ± 0.1	31.4 ± 0.4	2.1 ± 0.2	3.7 ± 0.1

GraphBAN	88.9 ± 0.3	94.4 ± 0.3	97.8 ± 0.2	88.5 ± 0.4	16.1 ± 0.5	22.9 ± 0.2	30.4 ± 0.6	1.9 ± 0.2	4.1 ± 0.3

**ExplainBind**	**91.2** ± **0.4**	**95.4** ± **0.2**	**99.3** ± **0.3**	**90.5** ± **0.6**	**55.6** ± **0.7**	**69.5** ± **0.2**	**74.6** ± **0.6**	**7.3** ± **0.3**	**8.7** ± **0.2**

## Data Availability

The InteractBind database is derived from publicly available protein–ligand complex structures deposited in the Protein Data Bank (PDB). InteractBind has been uploaded to the manuscript submission system for peer review. The source code required to reproduce the results of this study is publicly available at **GitHub:** https://github.com/ZhaohanM/ExplainBind. A user-friendly web interface is accessible via the **Demo UI:** https://huggingface.co/spaces/Zhaohan-Meng/ExplainBind.
