# Supplementary material for: ExplainBind: Explainable Physicochemical Determinants of Protein–Ligand Binding via Non-Covalent Interactions
Source: bioRxiv. 2026 May 19:2026.03.03.707476. Originally published 2026 Mar 5. Preprint. [Version 2] doi: 10.64898/2026.03.03.707476 (PMC13001423; doi:10.64898/2026.03.03.707476)
Supplement: Supplementary file 1 [file NIHPP2026.03.03.707476V2-supplement-1.pdf]

bioRxiv preprint doi: https://doi.org/10.64898/2026.03.03.707476; this version posted May 19, 2026. The copyright holder for this preprint (which was not certified by peer review) is the author/funder, who has granted bioRxiv a license to display the preprint in perpetuity. It is made available under a CC-BY 4.0 International license.

Supplementary Material

- **Preliminary**
  - Covalent bonds and non-covalent interactions
  - Binding site and binding pocket
  - Biological background of ACE and L2HGDH
- **Experimental Setting**
  - Curation of InteractBind dataset
  - Dataset splitting details
  - Binding-site and interaction evaluation metrics: BRHR and IHR
  - Baselines
  - Hyperparameter
  - Curation of ACE dataset
  - L2HGDH bio-assay setup
  - Docking for L2HGDH binders
- **Additional Results**
  - Binding-site localization and non-covalent interaction prediction
  - Case study details
  - Prioritization of high-activity ligands with wet-lab validation on L2HGDH
  - Efficiency analysis
  - Time complexity analysis
  - Evaluation of different encoders
  - Performance on public datasets of protein-ligand interaction prediction
  - Ablation study
  - OOD performance comparison

bioRxiv preprint doi: https://doi.org/10.64898/2026.03.03.707476; this version posted May 19, 2026. The copyright holder for this preprint (which was not certified by peer review) is the author/funder, who has granted bioRxiv a license to display the preprint in perpetuity. It is made available under a CC-BY 4.0 International license.

S.1 Preliminary

S.1.1 Covalent bonds and non-covalent interactions.

Molecular systems involve two fundamental types of interactions: covalent bonds and non-covalent interactions [4]. Covalent bonds govern intra-molecular organisation and are typically treated as fixed in modeling. In contrast, non-covalent, generally reversible interactions, including hydrogen bonds [46], salt bridges [47], van der Waals contacts [48], hydrophobic contacts [49], π–π stacking [50], and cation–π interactions [51], do not involve bond formation but arise from physicochemical compatibility between molecules. These interactions are predominantly inter-molecular and collectively determine protein–ligand binding, including affinity, specificity, and binding-site prediction. However, such local mechanistic details are typically absent from existing datasets. InteractBind addresses this limitation by explicitly representing such local interaction patterns through sequence token-level interaction maps.

S.1.2 Binding site and binding pocket.

In structural biology, a binding pocket generally refers to a broader three-dimensional region on the protein surface that can accommodate a ligand, often characterized by geometric cavities or surface concavities [52]. In contrast, a binding site is more specific and denotes the subset of protein residues that directly participate in ligand recognition through non-covalent interactions [53]. While binding pockets describe a spatial region that may potentially host a ligand, binding sites capture the actual interacting components that determine molecular recognition and binding specificity. Although the two concepts are closely related and sometimes used interchangeably, they differ in granularity and interpretability. Binding pocket identification is often based on structural geometry and provides a coarse description of possible ligand-accessible regions, whereas binding site annotation reflects the underlying specific physicochemical interactions that stabilize binding [54]. In many sequence-based settings where high-resolution structural information is limited, binding pocket definitions can be ambiguous, making it difficult to evaluate whether a model has learned the true interaction determinants [19]. In this work, we therefore focus on binding sites at the residue level, defined by experimentally derived structural interaction annotations. This formulation provides a more precise and mechanistically grounded target for evaluation, enabling fine-grained assessment of whether models recover the actual residues involved in protein–ligand prediction tasks.

S.1.3 Biological background of ACE and L2HGDH

ACE is a central regulator of the renin–angiotensin system, catalyzing the conversion of angiotensin I to the vasoconstrictor angiotensin II and thereby controlling blood pressure, vascular tone, and cardiovascular homeostasis [55, 56]. Dysregulated ACE activity is a key driver of hypertension and cardiovascular disease, making ACE inhibition a cornerstone therapeutic strategy [36, 57]. As a result, extensive human ACE bioactivity data are available in public repositories such as PubChem [58] spanning a wide range of chemical scaffolds and potencies (Supplementary Fig. S3). This rich and well-characterized bioactivity landscape

bioRxiv preprint doi: https://doi.org/10.64898/2026.03.03.707476; this version posted May 19, 2026. The copyright holder for this preprint (which was not certified by peer review) is the author/funder, who has granted bioRxiv a license to display the preprint in perpetuity. It is made available under a CC-BY 4.0 International license.

provides an ideal testbed for evaluating whether predictive models can reliably prioritize highly potent ligands based on inferred binding probabilities.

L2HGDH is a mitochondrial membrane–associated metabolic enzyme that catalyzes the oxidation of L-2-hydroxyglutarate (L-2-HG) to 2-oxoglutarate (2-OG); pathogenic mutations in *L2HGDH* impair this clearance and cause the neurometabolic disorder L-2-hydroxyglutaric aciduria (L-2-HGA) [59–61]. Genetic deficiency of L2HGDH in mice results in pathological accumulation of L-2-HG accompanied by leukoencephalopathy, neuroinflammation, and progressive neurodegeneration, consistent with the severe neurological phenotypes observed in human patients and motivating strategies aimed at restoring or enhancing L2HGDH activity [62]. In contrast, under acute cardiac ischemia and oxidative stress, L2HGDH loss–induced elevation of L-2-HG has been shown to preserve myocardial function by reprogramming glucose metabolism toward the pentose phosphate pathway and improving redox buffering, suggesting that transient and partial inhibition of L2HGDH may be beneficial in specific pathophysiological contexts [37]. Together, these findings establish L2HGDH as a context-dependent metabolic target for which both activation and inhibition are of potential therapeutic interest. In addition, L2HGDH is absent in the InteractBind training set and has no closely related homologs within it, enabling a stringent OOD evaluation of ExplainBind on an unseen target. Thus, we selected L2HGDH as an experimental (wet-lab) validation target and prioritized model-ranked compounds for experimental testing using in vitro assays of enzyme activity, enabling direct assessment of whether predicted binders act as functional inhibitors or activators of L2HGDH.

S.2 Experimental setting

S.2.1 Curation of InteractBind dataset

Structure collection and sample construction. We construct **InteractBind**, a large-scale non-covalent interaction supervised protein–ligand binding database, by collecting protein–ligand complex structures from the Protein Data Bank (PDB) and processed them using PyMOL. Structures are filtered to retain only those that contain at least one organic ligand that binds to the protein. Each retained structure underwent a standardized preprocessing pipeline. All inorganic ligands and crystallographic water molecules are removed. Moreover, complexes containing metal ions in either the ligand or the protein are excluded. If a PDB entry contained multiple organic ligands, distance-based filtering is applied to extract individual ligand–protein complexes: for each ligand, only protein chains within $0.5\,\mathrm{nm}$ of the ligand are retained, while others are discarded. As a result, PDB files containing multiple ligands are split into multiple complexes, each consisting of a single ligand and its interacting protein chain(s).

For each complex, the input data were then separated into two files while preserving original atomic coordinates. Protein structures were converted to Structure-aware (SA)/FASTA format. Structure-aware (SA) protein representations were derived via *Foldseek* [39], providing an explicit local structural context [40]. Ligands were converted from PDB to SMILES and subsequently to SELFIES representations.

bioRxiv preprint doi: https://doi.org/10.64898/2026.03.03.707476; this version posted May 19, 2026. The copyright holder for this preprint (which was not certified by peer review) is the author/funder, who has granted bioRxiv a license to display the preprint in perpetuity. It is made available under a CC-BY 4.0 International license.

To obtain labeled binding data, a docking-based strategy was employed. Focused docking was performed for each ligand–protein pair, with the search box centred on the original ligand position and each axis set to the ligand size plus a $0.5\,\mathrm{nm}$ buffer. Pairs with predicted binding affinities lower (i.e., stronger) than $-7.0\,\mathrm{kcal/mol}$ were labeled as positive samples. Negative samples were generated by randomly selecting ligands for each positive protein and performing global docking with the search box centered at the protein centroid and sized as the protein dimensions plus a $1.0\,\mathrm{nm}$ buffer. Pairs with top-ranked docking affinities higher (i.e., weaker) than $-5.0\,\mathrm{kcal/mol}$ were labeled as negative samples. This procedure was repeated until each positive protein was associated with at least one valid negative ligand.

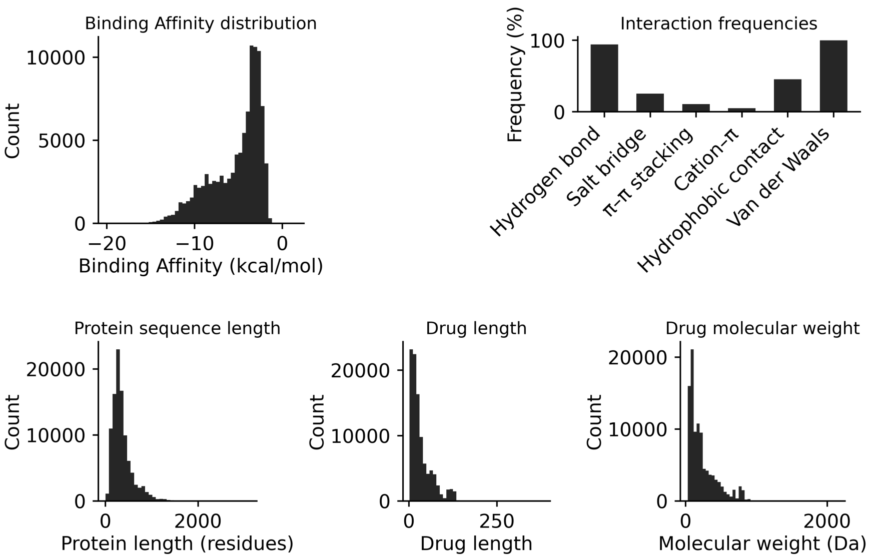

Figure S1: Distribution of features in **InteractBind**, including binding affinities, non-covalent interactions, protein sequence lengths, ligand lengths, and molecular weights.

Interaction annotation and strength modeling. Protein–ligand binding is governed by a diverse set of non-covalent interactions, including hydrogen bonds [46], salt bridges [47], van der Waals contacts [48], hydrophobic contacts [49], π–π stacking [50], and cation–π interactions [51]. These interactions jointly determine binding affinity, specificity, and structural complementarity, forming the physicochemical basis of molecular recognition [6]. Protein–ligand interactions for all positive samples in **InteractBind** are detected using rule-based criteria grounded in geometric and chemical constraints. Hydrogen bonds, salt bridges, π–π stacking, cation–π interactions, and hydrophobic contacts are identified using PLIP. van der Waals interactions, which are not captured by PLIP, are computed using GetContacts, and only attractive van der Waals interactions are retained. To quantify interaction strength, we adopt a distance-dependent piecewise linear decay function that maps interatomic or geometric distances to continuous values in the range $[10^{-6}, 1]$ within interaction-specific cutoff windows. Distance thresholds and geometric constraints for each non-covalent interaction are summarised in Table S1. Angular and geometric criteria, when applicable, are used as binary filters, while the distance-based decay controls the interaction strength.

bioRxiv preprint doi: https://doi.org/10.64898/2026.03.03.707476; this version posted May 19, 2026. The copyright holder for this preprint (which was not certified by peer review) is the author/funder, who has granted bioRxiv a license to display the preprint in perpetuity. It is made available under a CC-BY 4.0 International license.

Based on the detected interactions and their distance-dependent strengths, interaction-supervised attention maps are constructed at the token level. For each protein–ligand complex in **InteractBind**, an $m \times n$ matrix is generated, where m and n denote the numbers of ligand SELFIES tokens and protein FASTA tokens, respectively. Each matrix entry encodes the interaction strength between a ligand token and a protein residue. For each complex, six attention maps corresponding to individual non-covalent interactions are generated, together with an additional unified map obtained by summing all interaction-specific maps. All attention maps are stored using unique identifiers for efficient retrieval during training. After cleaning and preprocessing, **InteractBind** contains a total of **99,382 protein–ligand complexes**. These complexes form the basis for subsequent analyses and reflect substantial diversity in binding affinities, interaction patterns, and molecular properties, as shown in Fig. S1.

Table S1: Cutoffs and strength definitions for key non-covalent interactions in **InteractBind**.

Non-covalent Interaction	Parameter	Default Cutoff	Strength $s(d)$
Hydrogen Bond	Donor–Acceptor distance	$d \leq 4.1$ Å (active range: 2.2 Å $< d < 4.1$ Å)	$s(d) = \begin{cases} 1 - \frac{d - 2.2\,\text{Å}}{4.1\,\text{Å} - 2.2\,\text{Å}}, & 2.2\,\text{Å} < d < 4.1\,\text{Å} \\ 10^{-6}, & \text{otherwise} \end{cases}$
	Donor angle	$\geq 100°$	(angle used as filter; distance controls s)
Salt Bridge	Distance between charge centres	$d \leq 5.5$ Å (active range: 2.8 Å $< d < 5.5$ Å)	$s(d) = \begin{cases} 1 - \frac{d - 2.8\,\text{Å}}{5.5\,\text{Å} - 2.8\,\text{Å}}, & 2.8\,\text{Å} < d < 5.5\,\text{Å} \\ 10^{-6}, & \text{otherwise} \end{cases}$
π–π Stacking	Ring centroid distance	$d \leq 5.5$ Å (active range: 3.4 Å $< d < 5.5$ Å)	$s(d) = \begin{cases} 1 - \frac{d - 3.4\,\text{Å}}{5.5\,\text{Å} - 3.4\,\text{Å}}, & 3.4\,\text{Å} < d < 5.5\,\text{Å} \\ 10^{-6}, & \text{otherwise} \end{cases}$
	Angle deviation	$\leq 30°$	(geometry filter)
	Ring offset	≤ 2.0 Å	(geometry filter)
Cation–π Interaction	Cation–ring centroid distance	$d \leq 6.0$ Å (active range: 3.0 Å $< d < 6.0$ Å)	$s(d) = \begin{cases} 1 - \frac{d - 3.0\,\text{Å}}{6.0\,\text{Å} - 3.0\,\text{Å}}, & 3.0\,\text{Å} < d < 6.0\,\text{Å} \\ 10^{-6}, & \text{otherwise} \end{cases}$
Hydrophobic Contact	Distance between apolar atoms	$d \leq 4.0$ Å (active range: 3.0 Å $< d < 5.0$ Å)	$s(d) = \begin{cases} 1 - \frac{d - 3.0\,\text{Å}}{5.0\,\text{Å} - 3.0\,\text{Å}}, & 3.0\,\text{Å} < d < 5.0\,\text{Å} \\ 10^{-6}, & \text{otherwise} \end{cases}$
van der Waals Forces	Distance between non-H atoms	$d_0 \leq d < 4.5$ Å, where $d_0 = R_{\text{vdW}}(A) + R_{\text{vdW}}(B)$	$s(d) = \begin{cases} 1 - \frac{d - d_0}{4.5\,\text{Å} - d_0}, & d_0 < d < 4.5\,\text{Å} \\ 10^{-6}, & \text{otherwise} \end{cases}$

S.2.2 Dataset splitting

We evaluate model performance on nine datasets derived from the InteractBind database under two complementary evaluation settings: in-distribution (ID) and out-of-distribution (OOD). These datasets are designed to assess both standard predictive accuracy and generalization under controlled distribution shifts. For ID evaluation, we construct the **InteractBind (affinity)** dataset. Unlike conventional PLB benchmarks that treat all protein–ligand complexes as positive samples, this dataset explicitly accounts for binding strength by stratifying interactions according to binding affinity. Protein–ligand pairs with high affinity are regarded as positive samples, whereas weakly binding pairs are treated as negative samples, yielding a more challenging and discriminative ID evaluation setting.

To assess generalisability systematically, we further construct eight OOD datasets by explicitly controlling sequence similarity between the training and test sets. Each OOD dataset is divided into training, validation, and test splits with a ratio of 8:1:1. To examine the impact of protein sequence divergence, four protein-based OOD datasets are created: **InteractBind-P (25% OOD)**, **InteractBind-P (28% OOD)**, **InteractBind-**

bioRxiv preprint doi: https://doi.org/10.64898/2026.03.03.707476; this version posted May 19, 2026. The copyright holder for this preprint (which was not certified by peer review) is the author/funder, who has granted bioRxiv a license to display the preprint in perpetuity. It is made available under a CC-BY 4.0 International license.

P (31% OOD), and **InteractBind-P (33% OOD)**. In parallel, to evaluate ligand generalization, four ligand-based OOD datasets are constructed: **InteractBind-L (8% OOD)**, **InteractBind-L (35% OOD)**, **InteractBind-L (40% OOD)**, and **InteractBind-L (59% OOD)**. Compared with random in-distribution splits, these OOD settings provide a more realistic assessment of model generalization, reflecting practical drug discovery scenarios in which test proteins or molecules can differ substantially from those observed during training.

S.2.3 Binding-site and interaction evaluation metrics: BRHR and IHR

We evaluate fine-grained protein–ligand predictions from two complementary perspectives: binding-site localization and non-covalent interaction prediction. For a given protein–ligand pair, let $\mathbf{P} \in \mathbb{R}^{m\times n}$ denote the predicted interaction map, where m is the number of ligand tokens, n is the number of protein residue tokens, and P_{ij} is the predicted interaction score between ligand token d_i and protein residue token p_j. We first rank all entries in $\mathbf{P}$ and select the Top-K predicted token pairs:

$$E_K = \arg\mathrm{TopK}_{(i,j)}\, P_{ij}, \tag{1}$$

where E_K contains the index pairs of the K largest scores in $\mathbf{P}$.

Binding-site localization. Binding-site localization evaluates whether the model identifies the correct protein-side binding region. Since this task only concerns protein residues, we project the predicted token pairs in E_K onto the protein axis:

$$R_K = \{j \mid (i,j) \in E_K\}. \tag{2}$$

Let T_R denote the set of ground-truth binding residues. The sample-level <u>Binding Residue Hit Rate</u> (BRHR) is defined as

$$\mathrm{BRHR}(K) = \begin{cases} 1, & \text{if } R_K \cap T_R \neq \varnothing, \\ 0, & \text{otherwise.} \end{cases} \tag{3}$$

Thus, BRHR counts a prediction as correct if at least one predicted protein residue matches a ground-truth binding residue, regardless of the corresponding ligand token.

Non-covalent interaction prediction. Non-covalent interaction prediction evaluates whether the model recovers the annotated interaction at the token-pair level. Let T_E denote the set of ground-truth token pairs involved in non-covalent interactions. The sample-level <u>Interaction Hit Rate</u> (IHR) is defined as

$$\mathrm{IHR}(K) = \begin{cases} 1, & \text{if } E_K \cap T_E \neq \varnothing, \\ 0, & \text{otherwise.} \end{cases} \tag{4}$$

Unlike BRHR, IHR requires both sides of the predicted token pair to be correct, and therefore provides a stricter evaluation of non-covalent interaction prediction.

bioRxiv preprint doi: https://doi.org/10.64898/2026.03.03.707476; this version posted May 19, 2026. The copyright holder for this preprint (which was not certified by peer review) is the author/funder, who has granted bioRxiv a license to display the preprint in perpetuity. It is made available under a CC-BY 4.0 International license.

For both metrics, the dataset-level score is computed by averaging over all evaluated protein–ligand pairs:

$$\mathrm{HitRate}_{\mathrm{avg}}(K) = \frac{1}{N}\sum_{s=1}^{N}\mathrm{HitRate}_{s}(K), \tag{5}$$

where $\mathrm{HitRate}$ denotes either BRHR or IHR, and N is the number of evaluated samples. The same protocol can be applied either to a unified interaction map or separately to interaction-specific maps for different non-covalent interaction types.

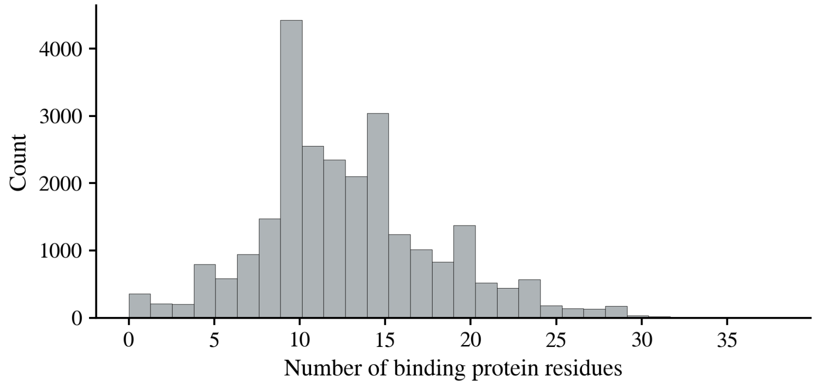

Figure S2: Distribution of ground-truth binding residues in the InteractBind dataset.

S.2.4 Baselines

We compare ExplainBind against the following seven representative baselines for the PLB task:

1. MolTrans [20], which tokenizes protein sequences and ligands into substructures, encodes them with transformer-based encoders, and applies an interaction module to model cross-modal binding patterns.
2. TransformerCPI [21], which represents proteins and compounds as sequences and employs transformer architectures to model their interactions for compound–protein interaction prediction.
3. HyperAttentionDTI [22], which introduces a hyper-attention mechanism to capture fine-grained interactions between protein residues and ligand atoms for drug–target interaction prediction.
4. PerceiverCPI [27], which adopts a Perceiver-based architecture to encode long protein and ligand sequences and model compound–protein interactions through latent cross-attention.
5. CAT-DTI [28], which uses cross-attention mechanisms to jointly model protein and ligand representations and improve interaction prediction.
6. DrugBAN [29], which encodes ligands with a graph neural network and proteins with a 1D CNN, and employs a bilinear attention network [63] to capture fine-grained residue–atom interactions before final prediction.
7. GraphBAN [30], which extends bilinear attention-based interaction modeling with graph neural representations for ligands and protein encoders for interaction prediction.

bioRxiv preprint doi: https://doi.org/10.64898/2026.03.03.707476; this version posted May 19, 2026. The copyright holder for this preprint (which was not certified by peer review) is the author/funder, who has granted bioRxiv a license to display the preprint in perpetuity. It is made available under a CC-BY 4.0 International license.

S.2.5 Hyperparameters

ExplainBind is implemented in Python 3.8 and the PyTorch framework (1.12.1)[1]. The computing device we use is the NVIDIA GeForce RTX A6000. Table S2 shows the parameters of the ExplainBind model and Table S3 lists the notations used in this paper with descriptions.

Table S2: Configuration parameters of ExplainBind.

Module	Hyperparameter	Value
Mini-batch	Batch size	64 (options: 64, 128)
Ligand Encoder	Foundation Model	HUBioDataLab/SELFormer
Protein Encoder	Foundation Model	westlake-repl/SaProt_650M_AF2
CAN	Attention heads	8 (corresponding to non-covalent interactions)
	Hidden dimension	512 (options: 32, 64, 128, 256, 512, 768)
	Integration strategies	Mean pooling (options: Mean pooling, CLS)
MLP	Hidden layer sizes	(1024, 512, 256)
	Activation	ReLU (options: Tanh, ReLU)
	Solver	AdamW (options: AdamW, Adam, RMSprop, Adadelta, LBFGS)
	Learning rate scheduler	CosineAnnealingLR (options: CosineAnnealingLR, StepLR)
	Initial learning rate	1×10^{-4} (options: 10^{-3} to 10^{-6})
	Maximum epoch	200

S.2.6 Curation of ACE dataset

ACE bioactivity data are curated from PubChem [58] in July 2024. Only human ACE bioassays reporting IC_{50} values are retained, while non-human assays and alternative bioactivity readouts are excluded. For compounds associated with multiple IC_{50} measurements, values with small relative variation are averaged, whereas compounds exhibiting large inter-assay discrepancies are removed to ensure data reliability and consistency. The corresponding human ACE amino acid sequence is retrieved from the UniProt database (P12821).

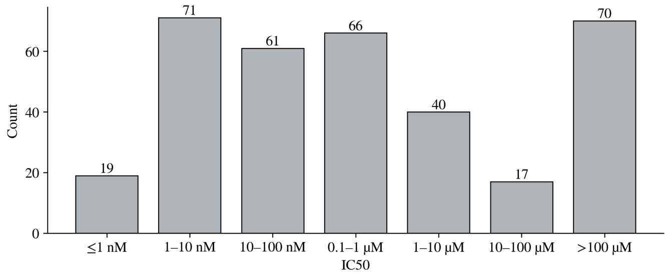

Figure S3: Distribution of ACE compound potency. Histogram of IC_{50} values for the curated human ACE dataset, grouped into logarithmic activity bins ranging from sub-nanomolar to >100 μM.

[1] https://pytorch.org/

bioRxiv preprint doi: https://doi.org/10.64898/2026.03.03.707476; this version posted May 19, 2026. The copyright holder for this preprint (which was not certified by peer review) is the author/funder, who has granted bioRxiv a license to display the preprint in perpetuity. It is made available under a CC-BY 4.0 International license.

Table S3: Notations and descriptions used in **ExplainBind**.

Notation	Description
$\mathbf{D} \in \mathbb{R}^{m \times h}$	Ligand token embeddings (length m, hidden size h)
$\mathbf{P} \in \mathbb{R}^{n \times h}$	Protein token embeddings (length n, hidden size h)
$\mathbf{Q}_d, \mathbf{K}_d, \mathbf{V}_d$	Query, key, and value matrices for ligand tokens
$\mathbf{Q}_p, \mathbf{K}_p, \mathbf{V}_p$	Query, key, and value matrices for protein tokens
$\mathbf{W}_q^d, \mathbf{W}_k^d, \mathbf{W}_v^d$	Projection weights for ligand Q/K/V
$\mathbf{W}_q^p, \mathbf{W}_k^p, \mathbf{W}_v^p$	Projection weights for protein Q/K/V
$\hat{\mathbf{A}}_{dp}^{(t)} \in \mathbb{R}^{m \times n}$	Predicted ligand→protein attention map for head t
$\hat{\mathbf{A}}_{pd}^{(t)} \in \mathbb{R}^{n \times m}$	Predicted protein→ligand attention map for head t
$\mathbf{A}^{(t)} \in \mathbb{R}^{m \times n}$	Ground-truth ligand→protein interaction map for head t
$\mathbf{A}_{\text{raw}}$	Unnormalised ground-truth interaction intensity used to construct $\{\mathbf{A}^{(t)}\}$
$\mathbf{P}_{\text{sa}}^{(t)}, \mathbf{D}_{\text{sa}}^{(t)}$	Self-attended protein/ligand features for head t
$\mathbf{P}^{*(t)}, \mathbf{D}^{*(t)}$	Head-specific fused representations (cross + self fusion)
$\mathbf{P}^*, \mathbf{D}^*$	Final fused sequences after concatenation across all heads
$\bar{\mathbf{D}}^*, \bar{\mathbf{P}}^*$	Mean-pooled ligand/protein fused embeddings
$\mathbf{F} = [\bar{\mathbf{D}}^*, \bar{\mathbf{P}}^*]$	Joint protein–ligand representation for classification
$p \in [0, 1]$	Predicted binding probability
$\mathcal{L}_{\text{cls}}$	Binary cross-entropy classification loss
$\mathcal{L}_{\text{att}}$	Masked KL-divergence attention alignment loss
$\mathcal{L}$	Total loss $(1 - \lambda)\mathcal{L}_{\text{cls}} + \lambda\mathcal{L}_{\text{att}}$
$\mathcal{T}$	Set of attention heads ($\lvert\mathcal{T}\rvert = 8$: six type-specific and two overall)
m, n	Sequence lengths of ligand and protein
h	Hidden dimension size
λ	Weight balancing classification and attention losses

The final curated dataset comprises 344 compounds with experimentally measured IC_{50} values spanning from ≤ 1 nM to > 100 μM, thereby covering more than five orders of magnitude in inhibitory potency. As shown in Fig. S3, the activity distribution is highly heterogeneous across potency regimes. Highly potent inhibitors ($IC_{50} \leq 1$ nM) are relatively rare, whereas the majority of compounds fall within the 1–10 nM, 10–100 nM, and sub-micromolar ranges. In contrast, a substantial fraction of weakly active or inactive compounds ($IC_{50} > 100$ μM) is also present.

This long-tailed and imbalanced potency distribution mirrors realistic medicinal chemistry screening data and provides a stringent testbed for evaluating the ability of ExplainBind to prioritise highly potent ligands from a large background of weaker binders.

S.2.7 L2HGDH bioassay setup

The expression construct for the *Drosophila melanogaster* orthologue of L2HGDH (dmL2HGDH) with N-terminal histidine and SUMO tags is provided by Jianping Ding [61]. The protein is expressed and purified as previously described [61] using B-PER Complete (Thermo) with HALT Protease Inhibitor

bioRxiv preprint doi: https://doi.org/10.64898/2026.03.03.707476; this version posted May 19, 2026. The copyright holder for this preprint (which was not certified by peer review) is the author/funder, who has granted bioRxiv a license to display the preprint in perpetuity. It is made available under a CC-BY 4.0 International license.

Cocktail (Thermo) for bacterial cell lysis. Purity is confirmed by UV-visible spectroscopy and mass spectrometry. For the activity assay, 10 μL of 40 nM dmL2HGDH in assay buffer (20 mM NaH_2PO_4, pH 7.4, 0.05% CHAPS, and 1% DMSO) is incubated with test compounds at 20 μM for 30 min at ambient temperature. The reaction is initiated by the addition of 10 μL substrate solution containing 120 μM L-2-hydroxyglutarate and 200 μM resazurin in assay buffer. Following 90 min incubation, fluorescence intensity is measured using a PHERAstar FSX plate reader (BMG LABTECH) with excitation at 540 nm and emission at 590 nm. With this assay, the top 200 predicted molecules are tested in duplicate. Compound activity is calculated as the percentage relative to the DMSO vehicle control.

Relative activity is defined as the percent change in enzyme activity in the presence of compound relative to the vehicle control:

$$\text{Relative Activity}(\%) = \frac{A_{\text{compound}} - A_{\text{control}}}{A_{\text{control}}} \times 100$$

where A_{compound} is the measured enzyme activity in the presence of compound, and A_{control} is the activity measured with vehicle control. Positive values indicate activation, whereas negative values indicate inhibition. Absolute relative activity ($|\text{Relative Activity}|$) is used to quantify the magnitude of functional modulation regardless of direction. The amino acid sequence used for model evaluation is derived from the crystal structure of dmL2HGDH (PDB ID: 8W75).

S.2.8 Docking for L2HGDH binders

Molecular docking is performed to evaluate the binding feasibility and plausible binding modes of candidate ligands to human L2HGDH using AutoDock Vina [7]. The enzyme conformation is derived from the L2HGDH crystal structure (PDB: 8W78, chain A) and prepared in PDBQT format with the flavin adenine dinucleotide (**FAD**) cofactor retained to preserve the native catalytic environment. Ligands are converted to PDBQT format and docked individually in a batch workflow using a blind-docking strategy. To ensure consistent docking across all ligands, a single docking search space is defined directly from the receptor structure. All `ATOM` and `HETATM` coordinates in the receptor PDBQT file are used to determine the axis-aligned bounding box of the receptor. The docking box center is set to $(8.43, -34.30, -18.29)$ Å, and the box dimensions are defined as $58.52 \times 69.63 \times 67.78$ Åalong the x, y, and z axes, respectively. These dimensions correspond to the full receptor coordinate span expanded by a fixed padding of 5 Å, ensuring coverage beyond a tight protein envelope and accommodating surface-accessible binding sites. For visualization, the protein and docking search space are rendered using `py3Dmol` [64]. The protein is displayed as a light-gray schematic representation; hetero atoms including the retained FAD cofactor are shown as sticks, and the docking box is overlaid as a gray wireframe cuboid. The view is automatically centered and zoomed to the structure to enable rapid inspection of docking box placement and coverage prior to large-scale docking.

bioRxiv preprint doi: https://doi.org/10.64898/2026.03.03.707476; this version posted May 19, 2026. The copyright holder for this preprint (which was not certified by peer review) is the author/funder, who has granted bioRxiv a license to display the preprint in perpetuity. It is made available under a CC-BY 4.0 International license.

S.3 Additional results

S.3.1 Binding-site localization and non-covalent interaction prediction

We report the quantitative results for the two fine-grained evaluation settings defined in Section S.2.3: binding-site localization on the unified interaction map and non-covalent interaction prediction on interaction-specific maps. For binding-site localization, we evaluate whether the Top-K predictions recover ground-truth protein-side binding residues after projecting predicted ligand–protein token pairs onto the protein axis. For non-covalent interaction prediction, we use a stricter token-pair criterion, requiring the predicted ligand–protein pair to match a ground-truth interaction pair for each annotated interaction type.

Table S4 reports the exact numerical values underlying the bar plots in the main text. The overall row corresponds to binding-site localization and is evaluated at the protein-residue level, whereas the six interaction-specific rows correspond to non-covalent interaction prediction and are evaluated at the ligand-token–protein-residue pair level. Overall binding-site localization improves from 55.6% at Top-1 to 74.6% at Top-5, indicating that true binding residues are increasingly recovered among the highest-ranked predictions. For non-covalent interaction prediction, π–π stacking achieves the highest Top-K hit rates across all thresholds, followed by salt bridges. Hydrogen bonding yields lower token-pair recovery, consistent with its greater sparsity and geometric specificity. These results show that the model captures both protein-side binding-site signals and fine-grained interaction-specific contact patterns.

Table S4: Top-K results for binding-site localization and non-covalent interaction prediction. The overall row reports protein-residue-level binding-site localization on the unified interaction map, whereas the six interaction-specific rows report pair-level non-covalent interaction prediction. The **highest** and <u>second-highest</u> values among the non-covalent interaction categories within each column are highlighted.

Top-K	1	2	3	4	5
**Binding-site localization**	55.6±0.7	64.6±0.4	69.5±0.2	72.2±0.4	74.6±0.6
**Non-covalent interaction prediction**					
van der Waals forces	31.5±2.0	32.9±1.7	33.9±1.9	34.5±1.6	35.0±1.4
Hydrogen bonding	27.2±1.6	28.5±1.9	29.3±1.4	29.8±1.8	30.2±1.2
Salt bridge	<u>43.1±1.7</u>	<u>44.2±1.5</u>	<u>45.1±2.2</u>	<u>45.7±1.6</u>	<u>46.2±1.3</u>
π–π stacking	**56.7±1.4**	**57.9±1.8**	**58.1±1.6**	**58.5±1.9**	**58.6±1.2**
Cation–π interaction	39.0±1.5	40.4±1.3	41.4±1.8	41.9±1.6	42.1±1.1
Hydrophobic contact	39.8±1.9	41.0±1.6	41.5±2.1	42.1±1.7	42.3±1.4

S.3.2 Case study details

A detailed analysis is presented below:

- **Homo sapiens CDK2–staurosporine** (Fig. 2d). The model correctly highlighted three non-covalent interactions—hydrogen bonds, hydrophobic interactions, and van der Waals interactions—that collectively anchor staurosporine within the CDK2 binding cleft. Key hydrogen bond donors or acceptors included GLU81, LEU83, ASP86, and GLN131, engaging the ligand's indolocarbazole backbone in close agreement with crystallographic data. Hydrophobic interactions are concentrated around ALA31, LYS33, PHE80, and ASP145, forming a compact apolar cage that stabilizes the

bioRxiv preprint doi: https://doi.org/10.64898/2026.03.03.707476; this version posted May 19, 2026. The copyright holder for this preprint (which was not certified by peer review) is the author/funder, who has granted bioRxiv a license to display the preprint in perpetuity. It is made available under a CC-BY 4.0 International license.

planar ring system. In addition, ILE10, GLY11, VAL64, PHE82, LEU83, HIS84, GLN85, and LEU134 contributed to van der Waals interactions along the aromatic scaffold, further reinforcing ligand binding within the pocket.

- **Sus scrofa elastase–4E4** (Fig. 2e). The ground-truth interaction map comprises four non-covalent interactions—hydrogen bonds, hydrophobic interactions, π–π stacking, and van der Waals interactions—all of which are accurately recovered by ExplainBind. The model identified hydrogen bonds between the inhibitor's carbonyl and amide groups and residues GLN185, SER188, and HIS45 within the catalytic triad region. Hydrophobic contacts clustered around VAL88, VAL209, and PHE208 provide the principal non-polar anchoring surface. Notably, HIS45 is also enriched in π–π stacking, highlighting dual aromatic–electrostatic stabilization within the substrate-recognition pocket. In addition, HIS45, CYS184, and GLN185 contributed to van der Waals interactions, further reinforcing local packing between the ligand and the catalytic groove.

- **Staphylococcus aureus DHFR–trimethoprim** (Fig. 2f). The model successfully reproduced the hydrogen bond network between the ligand's amino and methoxy groups and the enzyme's active-site residues LEU5, ASP27, and PHE92. Hydrophobic interactions are correctly localized to ILE50, LEU20, and PHE92, which surround the *p*-aminobenzyl ring of trimethoprim and stabilize its orientation within the pocket. Additionally, LEU5, VAL6, ALA7, LEU20, SER49, and PHE92 are predicted to contribute to van der Waals interactions, collectively forming a mixed polar–apolar microenvironment characteristic of the DHFR binding site.

S.3.3 Prioritization of high-activity ligands with wet-lab validation on L2HGDH

Table S5 presents a Top-K analysis of model predictions for ligand prioritization in the L2HGDH case study. Ligands are ranked by predicted binding probability, and we observe a clear enrichment of experimentally active molecules among the highest-ranked candidates. In particular, the mean |Relative Activity is substantially higher for small Top-K subsets and decreases monotonically as k increases, while the mean predicted probability remains close to unity for the top-ranked ligands. Consistently, molecules with $|\text{Activity}| \geq 25$ are predominantly concentrated within the high-confidence region, supporting the use of model confidence for guiding candidate selection in subsequent wet-lab validation.

Table S5: Top-K analysis of model predictions. We report the mean |Relative Activity, mean predicted probability, and the number of samples with $|\text{Activity}| \geq 25$ for each Top-K.

**Top-K**	**5**	**10**	**15**	**20**	**25**	**50**	**75**	**100**	**150**	**200**
**|Activity| Mean**	22.40	15.50	12.00	9.65	8.60	6.96	6.43	6.06	5.23	5.39
**Probability Mean**	1.0000	1.0000	1.0000	1.0000	0.9999	0.9981	0.9899	0.9674	0.8720	0.7513
$\|\text{Activity}\| \geq 25$	2	3	3	3	3	4	4	4	4	4

bioRxiv preprint doi: https://doi.org/10.64898/2026.03.03.707476; this version posted May 19, 2026. The copyright holder for this preprint (which was not certified by peer review) is the author/funder, who has granted bioRxiv a license to display the preprint in perpetuity. It is made available under a CC-BY 4.0 International license.

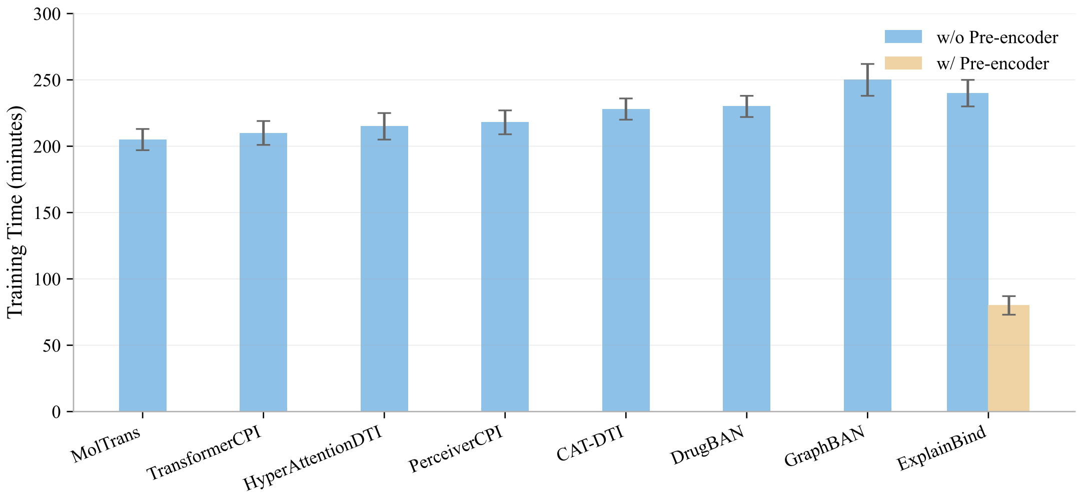

Figure S4: Comparison of training time on the InteractBind (affinity) dataset.

S.3.4 Efficiency analysis

Efficiency is a critical factor when scaling computational models to large drug discovery datasets, and it is strongly influenced by how molecular representations are managed. In ExplainBind, the ligand and protein encoders are frozen, so their embeddings remain unchanged during training. This enables a pre-encoding strategy, where all representations are computed once in advance and stored for reuse. In contrast, baselines such as DrugBAN and GraphBAN continuously update embeddings during training and therefore cannot benefit from this approach. As illustrated in Fig. S4, on the InteractBind (affinity) dataset the training time of ExplainBind without pre-encoding (about 220 minutes) is comparable to that of the bilinear baselines (DrugBAN $\sim$230 minutes; GraphBAN $\sim$250 minutes). With pre-encoding, however, the training time of ExplainBind is reduced to approximately 85 minutes. The bar chart clearly demonstrates this substantial gain in efficiency, which arises from avoiding repeated feature extraction. A limitation of pre-encoding is the additional memory required to store pre-computed embeddings, which may become a consideration when scaling to very large datasets.

S.3.5 Time complexity analysis

The feature dimensions of the representations generated by different foundation model encoders are fixed, but the dimensionality may differ across encoders. To enable fusion of protein and ligand representations, we use linear layers to project them into a common hidden dimension (h) of 512.

For the token-level interaction in the PLB task, the time complexity is dominated by the attention mechanism. With multi-head attention ($H = 8$), the computation of queries, keys, and values, as well as the softmax attention weights, requires

$$O(H \cdot m \cdot n \cdot h),$$

bioRxiv preprint doi: https://doi.org/10.64898/2026.03.03.707476; this version posted May 19, 2026. The copyright holder for this preprint (which was not certified by peer review) is the author/funder, who has granted bioRxiv a license to display the preprint in perpetuity. It is made available under a CC-BY 4.0 International license.

where m and n are the sequence lengths of ligand and protein tokens, respectively, and h is the hidden dimension. Since each head attends across all pairs of residue–level tokens, the $m \cdot n$ term (from the pairwise attention score computation) is the key factor. Thus, the overall time complexity of the supervised attention module is

$$O(m \cdot n \cdot h).$$

Table S6: Time complexity and parameter count of the supervised attention network (CAN).

Module	Complexity (O)	Parameters
CAN	$O(m \cdot n \cdot h)$	1.57M

In summary, the supervised attention network scales linearly with the hidden dimension and quadratically with the token lengths of protein and ligand sequences. While this introduces higher parameter counts compared to simpler bilinear modules, the explicit attention supervision enables mechanistically explainable residue–level interaction maps without prohibitive computational overhead.

S.3.6 Evaluation of different encoders

Table S7: Summary of foundation models used for encoding proteins and ligands. Protein encoders process either amino acid or structure-aware sequences, while ligand encoders accept SMILES or SELFIES representations.

Foundation Models	Model Name	HuggingFace Link	Input Type
Protein	ProteinBERT	Rostlab/prot_bert	Amino acid sequence
	ESM-2	facebook/esm2_t33_650M_UR50D	Amino acid sequence
	SaProt	westlake-repl/SaProt_650M_AF2	Structure-aware sequence
Ligand	ChemBERTa-2	seyonec/ChemBERTa-zinc-base-v1	SMILES
	MoLFormer	ibm-research/MoLFormer-XL-both-10pct	SMILES
	SELFormer	HUBioDataLab/SELFormer	SELFIES

Descriptions:

- **ProteinBERT** [65]: Pre-trained on large-scale amino acid sequences to capture evolutionary and contextual dependencies within protein families.
- **ESM-2** [41]: An evolutionary-scale protein language model trained on UniRef50, offering deep contextual representations from protein sequence space.
- **SaProt** [40]: Incorporates 3D structural priors by transforming protein structures into structure-aware sequences via `FoldSeek`.
- **ChemBERTa-2** [45]: A transformer-based molecular encoder trained on large SMILES corpora, providing chemically informed embeddings.
- **MoLFormer** [44]: A multi-task transformer model pre-trained on both molecular graphs and SMILES strings, enabling enhanced molecular generalization.

bioRxiv preprint doi: https://doi.org/10.64898/2026.03.03.707476; this version posted May 19, 2026. The copyright holder for this preprint (which was not certified by peer review) is the author/funder, who has granted bioRxiv a license to display the preprint in perpetuity. It is made available under a CC-BY 4.0 International license.

- **SELFormer** [42]: Utilises SELFIES as a chemically valid representation, improving robustness and representation stability in molecular encoding.

Our framework supports flexible substitution of these encoders with emerging state-of-the-art foundation models, ensuring continual adaptability and performance improvement as the field evolves.

Table S8: Performance comparison of different protein and ligand encoder combinations on the PDB (33% OOD) dataset. Ligand inputs are SMILES or SELFIES, while protein inputs are amino acid sequences or structure-aware representations. We highlight the **best** and <u>second best</u> results.

Ligand Input	Protein Input	Ligand Encoder	Protein Encoder	Accuracy	AUROC	AUPRC
SMILES	Amino acid sequence	ChemBERTa-2	ProteinBERT	0.796±0.007	0.902±0.004	0.894±0.005
SMILES	Amino acid sequence	ChemBERTa-2	ESM-2	0.794±0.006	0.900±0.003	0.896±0.004
SMILES	Structure-aware sequence	ChemBERTa-2	SaProt	0.807±0.005	0.927±0.003	0.899±0.003
SMILES	Amino acid sequence	MoLFormer	ProteinBERT	0.787±0.006	0.914±0.005	0.902±0.003
SMILES	Amino acid sequence	MoLFormer	ESM-2	0.792±0.005	0.917±0.004	0.901±0.006
SMILES	Structure-aware sequence	MoLFormer	SaProt	<u>0.811±0.005</u>	<u>0.945±0.003</u>	<u>0.927±0.008</u>
SELFIES	Amino acid sequence	SELFormer	ProteinBERT	0.798±0.003	0.930±0.004	0.912±0.006
SELFIES	Amino acid sequence	SELFormer	ESM-2	0.805±0.006	0.940±0.004	0.926±0.005
SELFIES	Structure-aware sequence	SELFormer	SaProt	**0.813±0.004**	**0.947±0.003**	**0.929±0.002**

Accurate token-level fusion is critical for the representational quality of both protein and ligand encoders. As summarized in Table S8, the best overall performance is achieved by combining SELFIES ligands encoded by SELFormer with SA protein sequences encoded by SaProt. This combination achieves the highest scores across all metrics, reflecting the advantages of self-consistent molecular representations on both sides. It is worth noting that SA sequences require extracting structure-aware features from protein structures using FoldSeek [39]. For proteins without experimentally determined structures, we also provide a model trained on the SELFIES & amino-acid sequence combination, which allows users to perform inference using only sequence-level input. This ensures compatibility with sequence-only datasets while maintaining strong performance. In practice, users can choose the appropriate model according to the available molecular inputs. For ligands, both **SMILES** and **SELFIES** are supported, while for proteins, either **structure-aware** or **amino acid** sequences can be used. As indicated in Table S8, the performance degradation from structure-aware to sequence-only setting is limited, confirming the robustness and generalisability of ExplainBind's foundation encoders across different molecular input formats.

S.3.7 Performance on public datasets of protein-ligand binding prediction

To assess further the robustness of ExplainBind, we additionally conducted experiments on three widely used public datasets: **BindingDB** [66], **BioSNAP** [67], and **Human** [21, 68]. In the ID setting, the model is trained solely with binary interaction labels, without the guidance of residue–level attention maps. As shown in Fig. S5, ExplainBind consistently outperforms a wide range of 8 baselines, indicating that it maintains strong predictive capability even without explicit structural supervision.

bioRxiv preprint doi: https://doi.org/10.64898/2026.03.03.707476; this version posted May 19, 2026. The copyright holder for this preprint (which was not certified by peer review) is the author/funder, who has granted bioRxiv a license to display the preprint in perpetuity. It is made available under a CC-BY 4.0 International license.

We further evaluated ExplainBind under OOD scenarios, where training and test sets differ substantially in protein and ligand distributions. The results demonstrate that ExplainBind achieves the best performance in two OOD settings and ranks second in the remaining case, presented in Fig. S6. These findings highlight that ExplainBind generalises well across heterogeneous benchmarks and remains competitive under challenging distribution shifts.

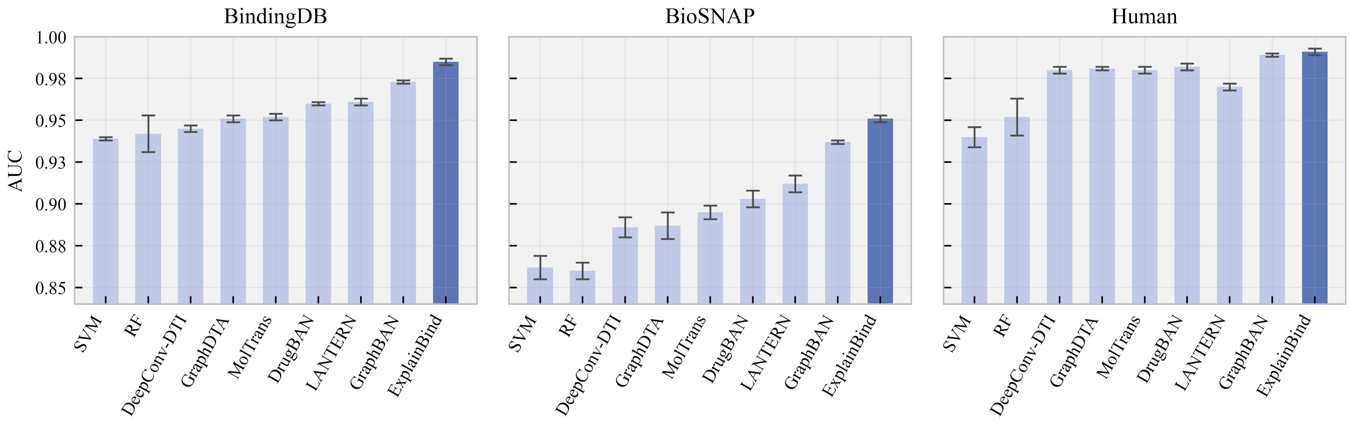

Figure S5: In-distribution performance comparison on three publicly available datasets without attention map supervision.

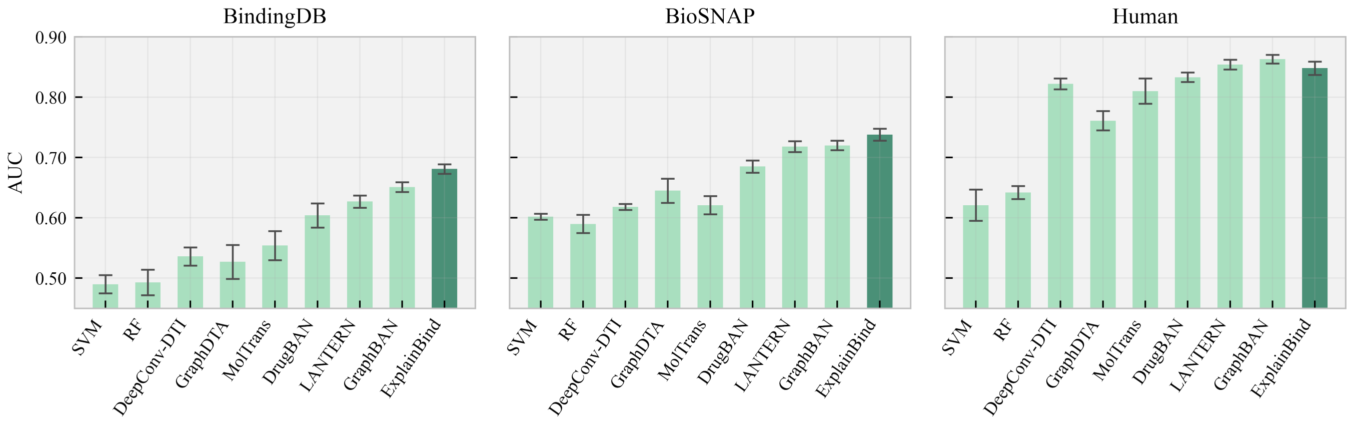

Figure S6: Out-of-distribution performance comparison on three publicly available datasets without attention map supervision.

S.3.8 Ablation study

To evaluate the contribution of individual components in ExplainBind, we conducted a comprehensive ablation analysis as summarized in Table S9. All variants share the same configuration as the final ExplainBind model. Replacing the pooling-based fusion with a [CLS] aggregation strategy results in a notable drop in performance (AUROC 0.940 $\rightarrow$ 0.927; Accuracy 0.805 $\rightarrow$ 0.784), confirming that mean pooling offers a more stable integration of token-level evidence. When replacing FASTA sequences with SA protein representations, the model achieves the best overall results (AUROC 0.947; Accuracy 0.817), suggesting that structural context can further strengthen the learned protein–ligand interactions. However, the SA variant requires structural features extracted through *Foldseek*, which depend on the availability

bioRxiv preprint doi: https://doi.org/10.64898/2026.03.03.707476; this version posted May 19, 2026. The copyright holder for this preprint (which was not certified by peer review) is the author/funder, who has granted bioRxiv a license to display the preprint in perpetuity. It is made available under a CC-BY 4.0 International license.

Table S9: Ablation results on the **PDB (33% OOD)** dataset. All results are based on the same ExplainBind configuration; each row removes or replaces one component for comparison.

Model / Setting	AUROC	AUPRC	Accuracy	Description
CLS Aggregation	0.927	0.913	0.784	using [CLS] aggregation (↓ vs ExplainBind)
Pooling Aggregation	0.940	0.926	0.805	using mean pooling for fusion
ExplainBind (SA)	0.947	0.933	0.817	variant using structure-aware protein representation (↑ vs ExplainBind)
ExplainBind	0.940	0.926	0.805	using raw FASTA sequence input
KL ratio = 0.1	0.937	0.920	0.794	
KL ratio = 0.2	0.936	0.923	0.797	
KL ratio = 0.3	**0.940**	**0.926**	**0.805**	varying KL loss ratio for attention alignment
KL ratio = 0.4	0.939	0.923	0.781	
KL ratio = 0.5	0.935	0.921	0.779	

of experimentally resolved or high-confidence predicted protein structures. In practice, many proteins are represented only by their amino acid sequences, making the FASTA-based ExplainBind a more universally applicable model. Overall, these findings demonstrate that pooling aggregation contributes to ExplainBind's robust and explainable performance. In Supplementary S.3.6, we further provide detailed comparisons of alternative protein–ligand encoder configurations.

S.3.9 OOD performance comparison

Table S10 reports the exact numerical results underlying the OOD performance line plots presented in the main text. Specifically, the table summarises the OOD performance of ExplainBind and baseline methods on the InteractBind-P datasets under varying OOD ratios (25–33%), evaluated using multiple metrics: AUROC, AUPRC, Accuracy, F1 score, Sensitivity, Specificity, and MCC [69].

bioRxiv preprint doi: https://doi.org/10.64898/2026.03.03.707476; this version posted May 19, 2026. The copyright holder for this preprint (which was not certified by peer review) is the author/funder, who has granted bioRxiv a license to display the preprint in perpetuity. It is made available under a CC-BY 4.0 International license.

Table S10: OOD performance comparison of ExplainBind and baselines. The **best** and second best results are highlighted. InteractBind-P (25–33% OOD) denotes the out-of-distribution test sets.

Model	AUROC	AUPRC	Accuracy	F1	Sensitivity	Specificity	MCC
**InteractBind-P (33% OOD)** — Out-of-distribution datasets (decreasing dissimilarity)							
MolTrans	0.795±0.015	0.765±0.016	0.678±0.012	0.668±0.013	0.660±0.015	0.682±0.012	0.230±0.013
TransformerCPI	0.822±0.014	0.784±0.015	0.692±0.012	0.681±0.013	0.675±0.014	0.696±0.012	0.241±0.012
HyperAttentionDTI	0.823±0.013	0.798±0.014	0.704±0.011	0.694±0.012	0.688±0.013	0.708±0.011	0.251±0.011
PerceiverCPI	0.848±0.011	0.826±0.012	0.724±0.010	0.714±0.011	0.708±0.012	0.728±0.010	0.268±0.010
DrugBAN	0.852±0.010	0.830±0.011	0.718±0.011	0.708±0.012	0.702±0.013	0.720±0.011	0.260±0.011
CAT-DTI	0.895±0.008	0.889±0.009	0.784±0.009	0.774±0.010	0.778±0.010	0.758±0.009	0.286±0.009
GraphBAN	0.920±0.005	0.904±0.006	0.790±0.007	0.782±0.008	0.772±0.008	0.798±0.007	0.313±0.007
ExplainBind	**0.940±0.004**	**0.926±0.005**	**0.805±0.006**	**0.793±0.007**	**0.788±0.007**	**0.810±0.006**	**0.330±0.007**
**InteractBind-P (31% OOD)**							
MolTrans	0.785±0.015	0.755±0.017	0.670±0.012	0.660±0.013	0.653±0.015	0.675±0.011	0.228±0.013
TransformerCPI	0.806±0.014	0.774±0.015	0.684±0.012	0.674±0.013	0.668±0.014	0.688±0.012	0.236±0.012
HyperAttentionDTI	0.805±0.013	0.789±0.014	0.696±0.011	0.686±0.012	0.680±0.013	0.700±0.011	0.246±0.011
PerceiverCPI	0.832±0.011	0.816±0.012	0.716±0.010	0.706±0.011	0.700±0.012	0.720±0.010	0.262±0.010
DrugBAN	0.842±0.010	0.820±0.012	0.710±0.011	0.700±0.012	0.695±0.013	0.712±0.011	0.255±0.011
CAT-DTI	0.879±0.008	0.874±0.009	0.744±0.009	0.734±0.010	0.728±0.010	0.748±0.009	0.279±0.009
GraphBAN	0.910±0.006	0.894±0.007	0.780±0.008	0.768±0.009	0.764±0.009	0.784±0.008	0.306±0.008
ExplainBind	**0.930±0.005**	**0.915±0.006**	**0.795±0.007**	**0.783±0.007**	**0.778±0.008**	**0.800±0.007**	**0.322±0.007**
**InteractBind-P (28% OOD)**							
MolTrans	0.768±0.016	0.740±0.017	0.658±0.013	0.648±0.014	0.640±0.016	0.660±0.012	0.225±0.013
TransformerCPI	0.781±0.014	0.756±0.015	0.672±0.012	0.662±0.013	0.655±0.014	0.676±0.012	0.229±0.012
HyperAttentionDTI	0.788±0.013	0.772±0.014	0.684±0.011	0.674±0.012	0.668±0.013	0.688±0.011	0.239±0.011
PerceiverCPI	0.814±0.011	0.801±0.012	0.706±0.010	0.696±0.011	0.690±0.012	0.710±0.010	0.255±0.010
DrugBAN	0.825±0.010	0.805±0.011	0.700±0.011	0.688±0.012	0.682±0.013	0.705±0.011	0.250±0.011
CAT-DTI	0.859±0.008	0.868±0.009	0.732±0.009	0.722±0.010	0.716±0.010	0.736±0.009	0.272±0.009
GraphBAN	0.900±0.006	0.882±0.007	0.768±0.008	0.758±0.009	0.750±0.009	0.774±0.008	0.296±0.008
ExplainBind	**0.910±0.005**	**0.895±0.006**	**0.782±0.007**	**0.770±0.007**	**0.765±0.008**	**0.788±0.007**	**0.310±0.007**
**InteractBind-P (25% OOD)**							
MolTrans	0.715±0.020	0.675±0.022	0.622±0.015	0.612±0.016	0.608±0.018	0.624±0.014	0.210±0.015
TransformerCPI	0.718±0.017	0.696±0.019	0.634±0.014	0.624±0.015	0.618±0.016	0.638±0.014	0.220±0.014
HyperAttentionDTI	0.752±0.016	0.710±0.018	0.646±0.013	0.636±0.014	0.630±0.015	0.650±0.013	0.228±0.014
PerceiverCPI	0.779±0.014	0.738±0.016	0.668±0.012	0.658±0.013	0.652±0.014	0.672±0.012	0.242±0.013
DrugBAN	0.781±0.011	0.775±0.014	0.689±0.013	0.678±0.012	0.670±0.015	0.715±0.012	0.235±0.013
CAT-DTI	0.801±0.011	0.800±0.013	0.714±0.011	0.704±0.012	0.708±0.013	0.706±0.011	0.252±0.012
GraphBAN	0.832±0.010	0.803±0.003	0.742±0.003	0.738±0.013	0.722±0.014	0.754±0.012	0.265±0.012
ExplainBind	**0.850±0.006**	**0.836±0.008**	**0.765±0.008**	**0.754±0.009**	**0.748±0.010**	**0.770±0.009**	**0.280±0.009**
