# Supplementary material for: Explainable Physicochemical Determinants of Protein–Ligand Binding via Non-Covalent Interactions
Source: bioRxiv. 2026 Mar 5:2026.03.03.707476. Preprint. [Version 1] doi: 10.64898/2026.03.03.707476 (PMC13001423; doi:10.64898/2026.03.03.707476)
Supplement: Supplementary file 1 [file NIHPP2026.03.03.707476v1-supplement-1.pdf]

bioRxiv preprint doi: https://doi.org/10.64898/2026.03.03.707476; this version posted March 5, 2026. The copyright holder for this preprint (which was not certified by peer review) is the author/funder, who has granted bioRxiv a license to display the preprint in perpetuity. It is made available under aCC-BY 4.0 International license.

Supplementary Material

- **Experimental Setting**
  - Curation of InteractBind Dataset
  - Evaluation settings of PLB
  - Evaluation settings of binding pocket hit rate
  - Baselines
  - Hyperparameter
  - Biological background of ACE and L2HGDH
  - Curation of ACE dataset
  - L2HGDH bio-Assay setup
  - Docking for L2HGDH binders
- **Additional Results**
  - Binding pocket hit rate
  - Prioritization of high-activity ligands with wet-lab validation on L2HGDH
  - Efficiency Analysis
  - Time Complexity Analysis
  - Evaluation of Different Encoders
  - Performance on Public Datasets
  - Ablation study
  - OOD Performance Comparison

bioRxiv preprint doi: https://doi.org/10.64898/2026.03.03.707476; this version posted March 5, 2026. The copyright holder for this preprint (which was not certified by peer review) is the author/funder, who has granted bioRxiv a license to display the preprint in perpetuity. It is made available under aCC-BY 4.0 International license.

S.1 Experimental Setting

S.1.1 Curation of InteractBind dataset

Structure collection and sample construction. We construct **InteractBind**, a large-scale non-covalent interaction supervised protein–ligand binding database, by collecting protein–ligand complex structures from the Protein Data Bank (PDB) format (version 3.2) using PyMOL. Structures were filtered to retain only those that contain at least one organic ligand that bind to the protein. Each retained structure underwent a standardized preprocessing pipeline. All inorganic ligands and crystallographic water molecules were removed. Moreover, complexes containing metal ions in either the ligand or the protein were excluded. If a PDB entry contained multiple organic ligands, distance-based filtering was applied to extract individual ligand–protein complexes: for each ligand, only protein chains within $0.5\,\mathrm{nm}$ of the ligand were retained, while others were discarded. As a result, PDB files containing multiple ligands were split into multiple complexes, each consisting of a single ligand and its interacting protein chain(s).

For each complex, the input data were then separated into two files while preserving original atomic coordinates. Protein structures were converted to SAPROT/FASTA format. Ligands were converted from PDB to SMILES and subsequently to SELFIES representations. To obtain labelled binding data, a docking-based strategy was employed. Focused docking was performed for each ligand–protein pair, with the search box centred on the original ligand position and each axis set to the ligand size plus a $0.5\,\mathrm{nm}$ buffer. Pairs with predicted binding affinities lower (i.e., stronger) than $-7.0\,\mathrm{kcal/mol}$ were labelled as positive samples. Negative samples were generated by randomly selecting ligands for each positive protein and performing global docking with the search box centred at the protein centroid and sized as the protein dimensions plus a $1.0\,\mathrm{nm}$ buffer. Pairs with top-ranked docking affinities higher (i.e., weaker) than $-5.0\,\mathrm{kcal/mol}$ were labelled as negative samples. This procedure was repeated until each positive protein was associated with at least one valid negative ligand.

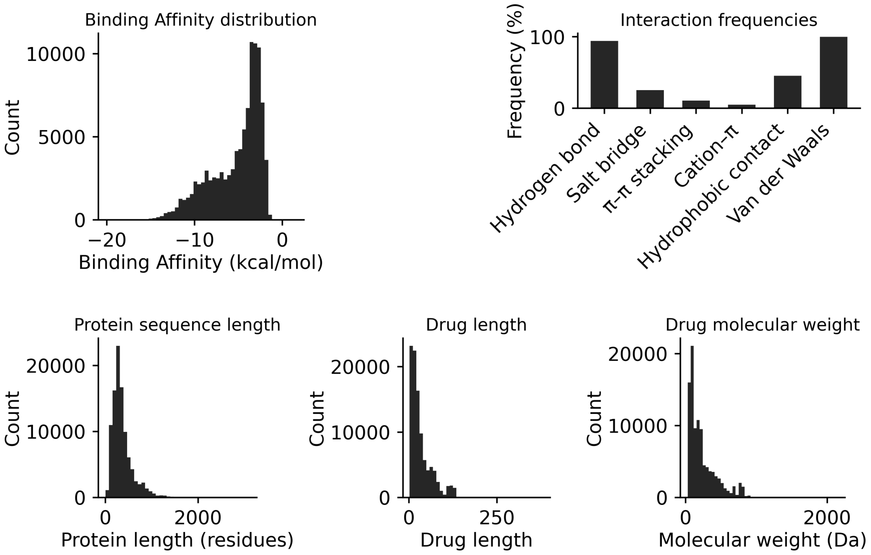

Figure S1: Distribution of features in **InteractBind**, including binding affinities, interaction types, protein sequence lengths, ligand lengths, and molecular weights.

Interaction annotation and strength modeling. Protein–ligand binding is governed by a diverse set of noncovalent interactions, including hydrogen bonds [4], salt bridges [5], van der Waals contacts [6], hydrophobic contacts [7], π–π stacking [8], and cation–π interactions [9]. These interactions jointly determine binding affinity, specificity, and structural complementarity, forming the physicochemical basis of molecular recognition [12]. Protein–ligand interactions for all positive samples in **InteractBind** were detected using rule-based criteria grounded in geometric and chemical constraints. Hydrogen bonds, salt bridges, π–π stacking, cation–π interactions, and hydrophobic contacts were identified using PLIP. Van der Waals interactions, which are not

bioRxiv preprint doi: https://doi.org/10.64898/2026.03.03.707476; this version posted March 5, 2026. The copyright holder for this preprint (which was not certified by peer review) is the author/funder, who has granted bioRxiv a license to display the preprint in perpetuity. It is made available under aCC-BY 4.0 International license.

captured by PLIP, were computed using GetContacts, and only attractive Van der Waals interactions were retained. To quantify interaction strength, we adopt a distance-dependent piecewise linear decay function that maps interatomic or geometric distances to continuous values in the range $[10^{-6}, 1]$ within interaction-specific cutoff windows. Distance thresholds and geometric constraints for each interaction type are summarised in Table S1. Angular and geometric criteria, when applicable, are used as binary filters, while the distance-based decay controls the interaction strength.

Based on the detected interactions and their distance-dependent strengths, interaction-supervised attention maps were constructed at the token level. For each protein–ligand complex in **InteractBind**, an $m \times n$ matrix was generated, where m and n denote the numbers of ligand SELFIES tokens and protein FASTA tokens, respectively. Each matrix entry encodes the interaction strength between a ligand token and a protein residue. For each complex, six attention maps corresponding to individual interaction types were generated, together with an additional unified map obtained by summing all interaction-specific maps. All attention maps were stored using unique identifiers for efficient retrieval during training. After cleaning and preprocessing, **InteractBind** contains a total of **99,382 protein–ligand complexes**. These complexes form the basis for subsequent analyses and reflect substantial diversity in binding affinities, interaction patterns, and molecular properties, as shown in Fig. S1.

Table S1: Cutoffs and strength definitions for key interaction types in **InteractBind**.

Interaction Type	Parameter	Default Cutoff	Strength $s(d)$
Hydrogen Bond	Donor–Acceptor distance	$d \leq 4.1\,\text{Å}$ (active range: $2.2\,\text{Å} < d < 4.1\,\text{Å}$)	$s(d) = \begin{cases} 1 - \dfrac{d - 2.2\,\text{Å}}{4.1\,\text{Å} - 2.2\,\text{Å}}, & 2.2\,\text{Å} < d < 4.1\,\text{Å} \\ 10^{-6}, & \text{otherwise} \end{cases}$
	Donor angle	$\geq 100°$	(angle used as filter; distance controls s)
Salt Bridge	Distance between charge centres	$d \leq 5.5\,\text{Å}$ (active range: $2.8\,\text{Å} < d < 5.5\,\text{Å}$)	$s(d) = \begin{cases} 1 - \dfrac{d - 2.8\,\text{Å}}{5.5\,\text{Å} - 2.8\,\text{Å}}, & 2.8\,\text{Å} < d < 5.5\,\text{Å} \\ 10^{-6}, & \text{otherwise} \end{cases}$
π–π Stacking	Ring centroid distance	$d \leq 5.5\,\text{Å}$ (active range: $3.4\,\text{Å} < d < 5.5\,\text{Å}$)	$s(d) = \begin{cases} 1 - \dfrac{d - 3.4\,\text{Å}}{5.5\,\text{Å} - 3.4\,\text{Å}}, & 3.4\,\text{Å} < d < 5.5\,\text{Å} \\ 10^{-6}, & \text{otherwise} \end{cases}$
	Angle deviation	$\leq 30°$	(geometry filter)
	Ring offset	$\leq 2.0\,\text{Å}$	(geometry filter)
Cation–π Interaction	Cation–ring centroid distance	$d \leq 6.0\,\text{Å}$ (active range: $3.0\,\text{Å} < d < 6.0\,\text{Å}$)	$s(d) = \begin{cases} 1 - \dfrac{d - 3.0\,\text{Å}}{6.0\,\text{Å} - 3.0\,\text{Å}}, & 3.0\,\text{Å} < d < 6.0\,\text{Å} \\ 10^{-6}, & \text{otherwise} \end{cases}$
Hydrophobic Contact	Distance between apolar atoms	$d \leq 4.0\,\text{Å}$ (active range: $3.0\,\text{Å} < d < 5.0\,\text{Å}$)	$s(d) = \begin{cases} 1 - \dfrac{d - 3.0\,\text{Å}}{5.0\,\text{Å} - 3.0\,\text{Å}}, & 3.0\,\text{Å} < d < 5.0\,\text{Å} \\ 10^{-6}, & \text{otherwise} \end{cases}$
Van der Waals Forces	Distance between non-H atoms	$d_0 \leq d < 4.5\,\text{Å}$, where $d_0 = R_{\text{vdW}}(A) + R_{\text{vdW}}(B)$	$s(d) = \begin{cases} 1 - \dfrac{d - d_0}{4.5\,\text{Å} - d_0}, & d_0 < d < 4.5\,\text{Å} \\ 10^{-6}, & \text{otherwise} \end{cases}$

S.1.2 Evaluation settings of PLB

We evaluate model performance on nine datasets derived from the InteractBind database under two complementary evaluation settings: in-distribution (ID) and out-of-distribution (OOD). These datasets are designed to assess both standard predictive accuracy and generalisation under controlled distribution shifts. For ID evaluation, we construct the **InteractBind (affinity)** dataset. Unlike conventional PLB benchmarks that treat all protein–ligand complexes as positive samples, this dataset explicitly accounts for binding strength by stratifying interactions according to binding affinity. Protein–ligand pairs with high affinity are regarded as positive samples, whereas weakly binding pairs are treated as negative samples, yielding a more challenging and discriminative ID evaluation setting.

To assess generalisability systematically, we further construct eight OOD datasets by explicitly controlling sequence similarity between the training and test sets. Each OOD dataset is divided into training, validation, and test splits with a ratio of 8:1:1. To examine the impact of protein sequence divergence, four protein-based OOD datasets are created: **InteractBind-P (25% OOD)**, **InteractBind-P (28% OOD)**, **InteractBind-P (31% OOD)**, and **InteractBind-P (33% OOD)**. In parallel, to evaluate ligand generalisation, four ligand-based OOD datasets are constructed: **InteractBind-L (8% OOD)**, **InteractBind-L (35% OOD)**, **InteractBind-L (40% OOD)**, and **InteractBind-L (59% OOD)**. Compared with random in-distribution splits, these OOD

bioRxiv preprint doi: https://doi.org/10.64898/2026.03.03.707476; this version posted March 5, 2026. The copyright holder for this preprint (which was not certified by peer review) is the author/funder, who has granted bioRxiv a license to display the preprint in perpetuity. It is made available under aCC-BY 4.0 International license.

settings provide a more realistic assessment of model generalisation, reflecting practical drug discovery scenarios in which test proteins or molecules can differ substantially from those observed during training.

S.1.3 Evaluation settings of binding residue hit rate

Accurate localisation of interacting residues is essential for interpreting protein–ligand binding mechanisms. ExplainBind is supervised using ground-truth residue-level interaction maps covering six distinct interaction types, and produces token-level attention scores over ligand–protein token pairs. For each interaction head, the predicted attention scores form a joint distribution over ligand tokens and protein residues.

We evaluate residue localisation using a Top-K hit-based metric, termed the *Binding Residue Hit Rate* (BRHR). Let $\mathcal{R}$ denote the set of all protein residues indexed along the sequence, and let $\mathcal{G} \subset \mathcal{R}$ be the set of ground-truth interacting residues derived from the residue-level interaction annotations. For a given protein–ligand pair, the model produces a head-specific attention map $P(l, r)$ over ligand tokens l and protein residues r, which is globally normalised such that $\sum_{l,r} P(l, r) = 1$. Residue predictions are obtained by first selecting the Top-K highest-scoring ligand–protein token pairs according to $P(l, r)$, and then projecting these pairs onto the protein axis. The resulting prediction set $\mathcal{R}_K \subset \mathcal{R}$ consists of the unique protein residues that appear in at least one of the Top-K selected token pairs.

We adopt an existence-based evaluation criterion: a prediction is considered correct if any residue in $\mathcal{R}_K$ falls within a predefined window around a ground-truth interacting residue. To account for different localisation tolerances, we consider two evaluation settings. Under a strict setting (window $= 0$), a predicted residue is counted as correct only if it exactly matches a ground-truth interacting residue. Under a relaxed setting (window $= 1$), a prediction is considered correct if it lies within a ± 1 residue window of any ground-truth interacting residue, reflecting local sequence-level uncertainty and conformational flexibility.

Formally, the Binding Residue Hit Rate for a given protein–ligand pair is defined as

$$\mathrm{BRHR}(K) = \mathbb{I}\Big(\exists\, r \in \mathcal{R}_K,\ \exists\, g \in \mathcal{G} \text{ s.t. } |r - g| \leq \delta\Big), \tag{1}$$

where $\mathbb{I}(\cdot)$ denotes the indicator function, r denotes a predicted protein residue index, g denotes a ground-truth interacting residue index, and $\delta \in \{0, 1\}$ specifies the allowable residue-index deviation.

The reported Top-K Binding Residue Hit Rate is obtained by averaging this indicator over all N protein–ligand pairs in the evaluation set,

$$\mathrm{BRHR}_{\mathrm{avg}}(K) = \frac{1}{N} \sum_{i=1}^{N} \mathrm{BRHR}_i(K). \tag{2}$$

The bar plot shown in Fig. S2 summarises the distribution of ground-truth interacting residues used in the BRHR evaluation.

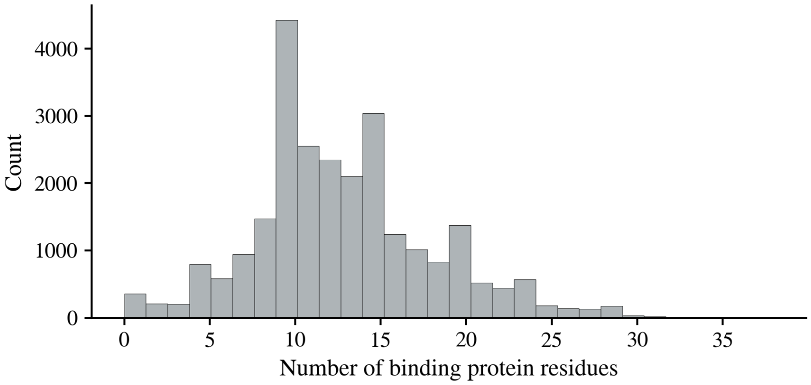

Figure S2: Distribution of ground-truth binding residues in the InteractBind dataset.

bioRxiv preprint doi: https://doi.org/10.64898/2026.03.03.707476; this version posted March 5, 2026. The copyright holder for this preprint (which was not certified by peer review) is the author/funder, who has granted bioRxiv a license to display the preprint in perpetuity. It is made available under aCC-BY 4.0 International license.

S.1.4 Baselines

We compare the performance of ExplainBind with the following eight models on the PLB task:

1. Support Vector Machine [56] trained on concatenated ECFP4 [53] ligand fingerprints and PSC [83] protein descriptors.
2. Random Forest [57] trained on the same concatenated ECFP4 and PSC features.
3. DeepConv-DTI [58] encodes ECFP4 ligand fingerprints with a fully connected network and protein sequences with a CNN plus max pooling. The outputs are concatenated and passed to a multilayer perceptron for prediction.
4. GraphDTA [59] represents ligands as graphs processed by GNNs and proteins as sequences encoded by a CNN. The resulting embeddings are concatenated for interaction prediction.
5. MolTrans [60] applies transformers to encode ligands and proteins and then uses a CNN-based interaction module to capture interaction patterns.
6. LANTERN [62] combines pre-trained language models with transformer-based fusion to predict multiple molecular interactions including PLB, DDI, and PPI without explicit structural input.
7. DrugBAN [61] encodes ligands with a graph convolutional network and proteins with a 1D CNN. A bilinear attention network [84] captures residue–level interactions, and the joint embedding is decoded by a fully connected network.
8. GraphBAN [63] encodes ligands with graph neural networks and proteins with a CNN. A bilinear attention network learns local pairwise interactions, and a multilayer perceptron produces the final prediction.

S.1.5 Hyperparameter

ExplainBind is implemented in Python 3.8 and the PyTorch framework (1.12.1)[1]. The computing device we use is the NVIDIA GeForce RTX A6000. Table S2 shows the parameters of the ExplainBind model and Table S3 lists the notations used in this paper with descriptions.

Table S2: Configuration parameters of ExplainBind.

Module	Hyperparameter	Value
Mini-batch	Batch size	64 (options: 64, 128)
Ligand Encoder	Foundation Model	HUBioDataLab/SELFormer
Protein Encoder	Foundation Model	westlake-repl/SaProt_650M_AF2
CAN	Attention heads	8 (corresponding to interaction types)
	Hidden dimension	512 (options: 32, 64, 128, 256, 512, 768)
	Integration strategies	Mean pooling (options: Mean pooling, CLS)
MLP	Hidden layer sizes	(1024, 512, 256)
	Activation	ReLU (options: Tanh, ReLU)
	Solver	AdamW (options: AdamW, Adam, RMSprop, Adadelta, LBFGS)
	Learning rate scheduler	CosineAnnealingLR (options: CosineAnnealingLR, StepLR)
	Initial learning rate	1×10^{-4} (options: 10^{-3} to 10^{-6})
	Maximum epoch	200

[1] https://pytorch.org/

bioRxiv preprint doi: https://doi.org/10.64898/2026.03.03.707476; this version posted March 5, 2026. The copyright holder for this preprint (which was not certified by peer review) is the author/funder, who has granted bioRxiv a license to display the preprint in perpetuity. It is made available under aCC-BY 4.0 International license.

Table S3: Notations and descriptions used in **ExplainBind**.

Notation	Description
$\mathbf{D} \in \mathbb{R}^{m\times h}$	Ligand token embeddings (length m, hidden size h)
$\mathbf{P} \in \mathbb{R}^{n\times h}$	Protein token embeddings (length n, hidden size h)
$\mathbf{Q}_d, \mathbf{K}_d, \mathbf{V}_d$	Query, key, and value matrices for ligand tokens
$\mathbf{Q}_p, \mathbf{K}_p, \mathbf{V}_p$	Query, key, and value matrices for protein tokens
$\mathbf{W}_q^d, \mathbf{W}_k^d, \mathbf{W}_v^d$	Projection weights for ligand Q/K/V
$\mathbf{W}_q^p, \mathbf{W}_k^p, \mathbf{W}_v^p$	Projection weights for protein Q/K/V
$\hat{\mathbf{A}}_{dp}^{(t)} \in \mathbb{R}^{m\times n}$	Predicted ligand→protein attention map for head t
$\hat{\mathbf{A}}_{pd}^{(t)} \in \mathbb{R}^{n\times m}$	Predicted protein→ligand attention map for head t
$\mathbf{A}^{(t)} \in \mathbb{R}^{m\times n}$	Ground-truth ligand→protein interaction map for head t
$\mathbf{A}_{\text{raw}}$	Unnormalised ground-truth interaction intensity used to construct $\{\mathbf{A}^{(t)}\}$
$\mathbf{P}_{\text{sa}}^{(t)}, \mathbf{D}_{\text{sa}}^{(t)}$	Self-attended protein/ligand features for head t
$\mathbf{P}^{*(t)}, \mathbf{D}^{*(t)}$	Head-specific fused representations (cross + self fusion)
$\mathbf{P}^*, \mathbf{D}^*$	Final fused sequences after concatenation across all heads
$\bar{\mathbf{D}}^*, \bar{\mathbf{P}}^*$	Mean-pooled ligand/protein fused embeddings
$\mathbf{F} = [\bar{\mathbf{D}}^*, \bar{\mathbf{P}}^*]$	Joint protein–ligand representation for classification
$p \in [0, 1]$	Predicted binding probability
$\mathcal{L}_{\text{cls}}$	Binary cross-entropy classification loss
$\mathcal{L}_{\text{att}}$	Masked KL-divergence attention alignment loss
$\mathcal{L}$	Total loss $(1-\lambda)\mathcal{L}_{\text{cls}} + \lambda\mathcal{L}_{\text{att}}$
$\mathcal{T}$	Set of attention heads ($\lvert\mathcal{T}\rvert = 8$: six type-specific and two overall)
m, n	Sequence lengths of ligand and protein
h	Hidden dimension size
λ	Weight balancing classification and attention losses

S.1.6 Biological background of ACE and L2HGDH

ACE is a central regulator of the renin–angiotensin system, catalyzing the conversion of angiotensin I to the vasoconstrictor angiotensin II and thereby controlling blood pressure, vascular tone, and cardiovascular homeostasis [85, 86]. Dysregulated ACE activity is a key driver of hypertension and cardiovascular disease, making ACE inhibition a cornerstone therapeutic strategy [68, 69]. As a result, extensive human ACE bioactivity data are available in public repositories such as PubChem [87] spanning a wide range of chemical scaffolds and potencies (Supplementary Fig. S3). This rich and well-characterized bioactivity landscape provides an ideal testbed for evaluating whether predictive models can reliably prioritize highly potent ligands based on inferred binding probabilities.

L2HGDH is a mitochondrial membrane–associated metabolic enzyme that catalyzes the oxidation of L-2-hydroxyglutarate (L-2-HG) to 2-oxoglutarate (2-OG); pathogenic mutations in *L2HGDH* impair this clearance and cause the neurometabolic disorder L-2-hydroxyglutaric aciduria (L-2-HGA) [70–72]. Genetic deficiency of L2HGDH in mice results in pathological accumulation of L-2-HG accompanied by leukoencephalopathy, neuroinflammation, and progressive neurodegeneration, consistent with the severe neurological phenotypes observed in human patients and motivating strategies aimed at restoring or enhancing L2HGDH activity [73]. In contrast, under acute cardiac ischemia and oxidative stress, L2HGDH loss–induced elevation of L-2-HG has been shown to preserve myocardial function by reprogramming glucose metabolism toward the pentose phosphate pathway and improving redox buffering, suggesting that transient and partial inhibition of L2HGDH may be beneficial in specific pathophysiological contexts [74]. Together, these findings establish L2HGDH as a context-dependent metabolic target for

bioRxiv preprint doi: https://doi.org/10.64898/2026.03.03.707476; this version posted March 5, 2026. The copyright holder for this preprint (which was not certified by peer review) is the author/funder, who has granted bioRxiv a license to display the preprint in perpetuity. It is made available under aCC-BY 4.0 International license.

which both activation and inhibition are of potential therapeutic interest. In addition, L2HGDH was absent in the InteractBind training set and has no closely related homologs within it, enabling a stringent OOD evaluation of ExplainBind on an unseen target. Thus, we selected L2HGDH as an experimental (wet-lab) validation target and prioritized model-ranked compounds for experimental testing using in vitro assays of enzyme activity, enabling direct assessment of whether predicted binders act as functional inhibitors or activators of L2HGDH.

S.1.7 Curation of ACE dataset

ACE bioactivity data were curated from PubChem [87] in July 2024. Only human ACE bioassays reporting IC_{50} values were retained, while non-human assays and alternative bioactivity readouts were excluded. For compounds associated with multiple IC_{50} measurements, values with small relative variation were averaged, whereas compounds exhibiting large inter-assay discrepancies were removed to ensure data reliability and consistency. The corresponding human ACE amino acid sequence was retrieved from the UniProt database (P12821).

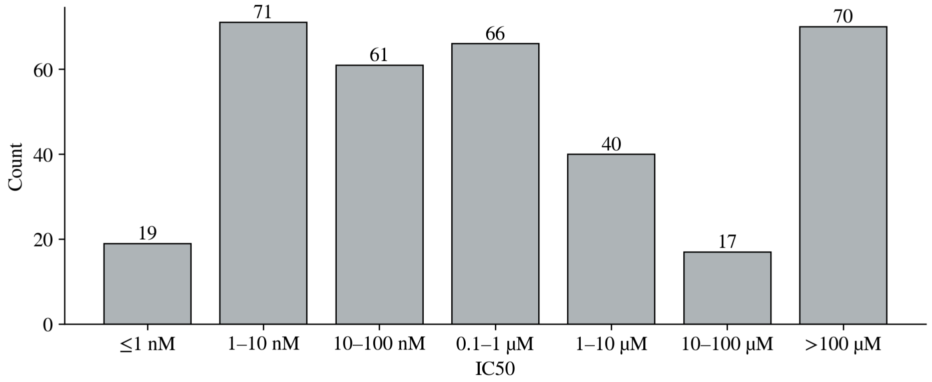

Figure S3: Distribution of ACE compound potency. Histogram of IC_{50} values for the curated human ACE dataset, grouped into logarithmic activity bins ranging from sub-nanomolar to >100 μM.

The final curated dataset comprises 344 compounds with experimentally measured IC_{50} values spanning from $\leq$ 1 nM to $>$ 100 μM, thereby covering more than five orders of magnitude in inhibitory potency. As shown in Fig. S3, the activity distribution is highly heterogeneous across potency regimes. Highly potent inhibitors ($IC_{50} \leq 1$ nM) are relatively rare, whereas the majority of compounds fall within the 1–10 nM, 10–100 nM, and sub-micromolar ranges. In contrast, a substantial fraction of weakly active or inactive compounds ($IC_{50} > 100$ nM) is also present.

This long-tailed and imbalanced potency distribution mirrors realistic medicinal chemistry screening data and provides a stringent testbed for evaluating the ability of ExplainBind to prioritise highly potent ligands from a large background of weaker binders.

S.1.8 L2HGDH Bioassay Setup

The expression construct for the *Drosophila melanogaster* orthologue of L2HGDH (dmL2HGDH) with N-terminal histidine and SUMO tags was provided by Jianping Ding [72]. The protein was expressed and purified as previously described [72] using B-PER Complete (Thermo) with HALT Protease Inhibitor Cocktail (Thermo) for bacterial cell lysis. Purity was confirmed by UV-visible spectroscopy and mass spectrometry. For the activity assay, 10 μL of 40 nM dmL2HGDH in assay buffer (20 mM NaH_2PO_4, pH 7.4, 0.05% CHAPS, and 1% DMSO) was incubated with test compounds at 20 μM for 30 min at ambient temperature. The reaction was initiated by the addition of 10 μL substrate solution containing 120 μM L-2-hydroxyglutarate and 200 μM resazurin in assay buffer. Following 90 min incubation, fluorescence intensity was measured using a PHERAstar FSX plate reader (BMG LABTECH) with excitation at 540 nm and emission at 590 nm. With this assay, 200 molecules in top prediction were tested in duplicate. Compound activity was calculated as the percentage relative to the DMSO vehicle control.

Relative activity was defined as the percent change in enzyme activity in the presence of compound relative to the vehicle control:

bioRxiv preprint doi: https://doi.org/10.64898/2026.03.03.707476; this version posted March 5, 2026. The copyright holder for this preprint (which was not certified by peer review) is the author/funder, who has granted bioRxiv a license to display the preprint in perpetuity. It is made available under aCC-BY 4.0 International license.

$$\text{Relative Activity}(\%) = \frac{A_{\text{compound}} - A_{\text{control}}}{A_{\text{control}}} \times 100$$

where A_{compound} is the measured enzyme activity in the presence of compound, and A_{control} is the activity measured with vehicle control. Positive values indicate activation, whereas negative values indicate inhibition. Absolute relative activity (|Relative Activity|) was used to quantify the magnitude of functional modulation regardless of direction. The amino acid sequence used for model evaluation was derived from the crystal structure of dmL2HGDH (PDB ID: 8W75).

S.1.9 Docking for L2HGDH binders

Molecular docking was performed to evaluate the binding feasibility and plausible binding modes of candidate ligands to human L2HGDH using AutoDock Vina [14]. The enzyme conformation was derived from the L2HGDH crystal structure (PDB: 8W78, chain A) and prepared in PDBQT format with the flavin adenine dinucleotide (**FAD**) cofactor retained to preserve the native catalytic environment. Ligands were converted to PDBQT format and docked individually in a batch workflow using a blind-docking strategy. To ensure consistent docking across all ligands, a single docking search space was defined directly from the receptor structure. All `ATOM` and `HETATM` coordinates in the receptor PDBQT file were used to determine the axis-aligned bounding box of the receptor. The docking box center was set to (8.43, −34.30, −18.29) Å, and the box dimensions were defined as 58.52 × 69.63 × 67.78 Åalong the x, y, and z axes, respectively. These dimensions correspond to the full receptor coordinate span expanded by a fixed padding of 5 Å, ensuring coverage beyond a tight protein envelope and accommodating surface-accessible binding sites. For visualization, the protein and docking search space were rendered using `py3Dmol` [88]. The protein was displayed as a light-gray schematic representation; hetero atoms including the retained FAD cofactor were shown as sticks, and the docking box was overlaid as a gray wireframe cuboid. The view was automatically centered and zoomed to the structure to enable rapid inspection of docking box placement and coverage prior to large-scale docking.

bioRxiv preprint doi: https://doi.org/10.64898/2026.03.03.707476; this version posted March 5, 2026. The copyright holder for this preprint (which was not certified by peer review) is the author/funder, who has granted bioRxiv a license to display the preprint in perpetuity. It is made available under aCC-BY 4.0 International license.

S.2 Additional Results

S.2.1 Binding residue hit rate

We evaluate the binding residue localisation capability of the model using the binding residue hit rate (BRHR), which measures whether ground-truth interacting residues are recovered within the top-k ranked protein tokens. Tables S4 and S5 report the exact numerical values underlying the bar plots shown in the main text, with Top-k results presented for $k = 1$ to 5. As shown in Table S4, the model exhibits a consistent improvement in BRHR as k increases under the strict matching criterion (window = 0), indicating that true binding residues are increasingly captured among the highest-ranked predictions. Performance trends are consistent across different interaction types, with van der Waals forces and salt bridge generally achieving higher localization accuracy than more sparse interaction categories such as cation–π interactions. When allowing a relaxed spatial tolerance (window = 1), Table S5 shows a systematic increase in BRHR across all values of k and interaction types. This improvement reflects the robustness of the predicted attention patterns, demonstrating that the model is able to localize binding residues accurately even under slight positional shifts. Overall, these results confirm that the proposed method captures meaningful and interpretable binding residue signals at a fine-grained, residue-level resolution.

Table S4: Top-k binding residue hit rate accuracy for the overall PLB task and for each specific interaction type (window = 0). The **highest** and <u>second-highest</u> values within each column are highlighted.

**Top-k**	**1**	**2**	**3**	**4**	**5**
**Binding Residue Hit Rate**	55.6±0.018	64.6±0.021	69.5±0.015	72.2±0.019	74.6±0.013
**Different Interaction Types**					
Van der Waals forces	<u>46.7±0.020</u>	**55.5±0.017**	**60.3±0.019**	**64.3±0.016**	**67.1±0.014**
Hydrogen bonding	39.1±0.016	47.4±0.019	52.2±0.014	55.2±0.018	58.1±0.012
Salt bridge	**47.4±0.017**	<u>53.9±0.015</u>	<u>57.5±0.022</u>	<u>60.0±0.016</u>	<u>61.4±0.013</u>
π–π stacking	45.0±0.014	49.1±0.018	51.0±0.016	53.1±0.019	55.4±0.012
Cation–π interaction	34.3±0.015	37.8±0.013	40.2±0.018	42.2±0.016	44.6±0.011
Hydrophobic contact	40.0±0.019	47.9±0.016	51.8±0.021	54.5±0.017	56.3±0.014

Table S5: Top-k binding residue hit rate accuracy for the overall PLB task and for each specific interaction type (window = 1). The **highest** and <u>second-highest</u> values within each column are highlighted.

**Top-k**	**1**	**2**	**3**	**4**	**5**
**Binding Residue Hit Rate**	62.6±0.019	71.1±0.016	76.0±0.018	78.6±0.014	80.5±0.012
**Different Interaction Types**					
Van der Waals forces	**55.1±0.017**	**63.7±0.020**	**68.4±0.015**	**72.1±0.018**	**74.4±0.013**
Hydrogen bonding	46.9±0.016	55.5±0.019	<u>60.0±0.014</u>	<u>62.7±0.017</u>	<u>65.2±0.012</u>
Salt bridge	<u>49.3±0.018</u>	<u>55.9±0.016</u>	59.4±0.021	62.1±0.015	63.6±0.013
π–π stacking	45.4±0.015	50.0±0.017	52.4±0.014	54.5±0.016	56.6±0.012
Cation–π interaction	34.6±0.014	38.4±0.013	40.8±0.018	42.8±0.016	44.6±0.011
Hydrophobic contact	44.0±0.019	51.9±0.016	55.9±0.020	58.7±0.017	60.3±0.014

bioRxiv preprint doi: https://doi.org/10.64898/2026.03.03.707476; this version posted March 5, 2026. The copyright holder for this preprint (which was not certified by peer review) is the author/funder, who has granted bioRxiv a license to display the preprint in perpetuity. It is made available under aCC-BY 4.0 International license.

S.2.2 Prioritization of high-activity ligands with wet-lab validation on L2HGDH

Table S6 presents a Top-k analysis of model predictions for ligand prioritisation in the L2HGDH case study. Ligands are ranked by predicted binding probability, and we observe a clear enrichment of experimentally active molecules among the highest-ranked candidates. In particular, the mean |Relative Activity is substantially higher for small Top-k subsets and decreases monotonically as k increases, while the mean predicted probability remains close to unity for the top-ranked ligands. Consistently, molecules with $|\text{Activity}| \geq 25$ are predominantly concentrated within the high-confidence region, supporting the use of model confidence for guiding candidate selection in subsequent wet-lab validation.

Table S6: Top-k analysis of model predictions. We report the mean |Relative Activity, mean predicted probability, and the number of samples with $|\text{Activity}| \geq 25$ for each Top-k.

**Top-k**	**5**	**10**	**15**	**20**	**25**	**50**	**75**	**100**	**150**	**200**
**|Activity| Mean**	22.40	15.50	12.00	9.65	8.60	6.96	6.43	6.06	5.23	5.39
**Probability Mean**	1.0000	1.0000	1.0000	1.0000	0.9999	0.9981	0.9899	0.9674	0.8720	0.7513
**Count**$_{\|\text{Activity}\|\geq 25}$	2	3	3	3	3	4	4	4	4	4

S.2.3 Efficiency analysis

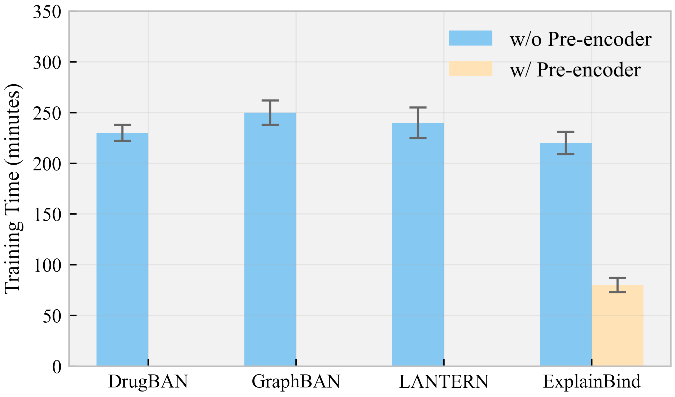

Figure S4: Comparison of training time on the InteractBind (affinity) dataset.

Efficiency is a critical factor when scaling computational models to large drug discovery datasets, and it is strongly influenced by how molecular representations are managed. In ExplainBind, the ligand and protein encoders are frozen, so their embeddings remain unchanged during training. This enables a pre-encoding strategy, where all representations are computed once in advance and stored for reuse. In contrast, baselines such as DrugBAN and GraphBAN continuously update embeddings during training and therefore cannot benefit from this approach. As illustrated in Fig. S4, on the InteractBind (affinity) dataset the training time of ExplainBind without pre-encoding (about 220 minutes) is comparable to that of the bilinear baselines (DrugBAN $\sim$230 minutes; GraphBAN $\sim$250 minutes). With pre-encoding, however, the training time of ExplainBind is reduced to approximately 85 minutes. The bar chart clearly demonstrates this substantial gain in efficiency, which arises from avoiding repeated feature extraction. A limitation of pre-encoding is the additional memory required to store pre-computed embeddings, which may become a consideration when scaling to very large datasets.

S.2.4 Time complexity analysis

The feature dimensions of the representations generated by different foundation model encoders are fixed, but the dimensionality may differ across encoders. To enable fusion of protein and ligand representations, we use linear layers to project them into a common hidden dimension (h) of 512.

bioRxiv preprint doi: https://doi.org/10.64898/2026.03.03.707476; this version posted March 5, 2026. The copyright holder for this preprint (which was not certified by peer review) is the author/funder, who has granted bioRxiv a license to display the preprint in perpetuity. It is made available under aCC-BY 4.0 International license.

For the token-level interaction in the PLB task, the time complexity is dominated by the attention mechanism. With multi-head attention ($H = 8$), the computation of queries, keys, and values, as well as the softmax attention weights, requires

$$O(H \cdot m \cdot n \cdot h),$$

where m and n are the sequence lengths of ligand and protein tokens, respectively, and h is the hidden dimension. Since each head attends across all pairs of residue–level tokens, the $m \cdot n$ term (from the pairwise attention score computation) is the key factor. Thus, the overall time complexity of the supervised attention module is

$$O(m \cdot n \cdot h).$$

Table S7: Time complexity and parameter count of the supervised attention network (CAN).

Module	Complexity (O)	Parameters
CAN	$O(m \cdot n \cdot h)$	1.57M

In summary, the supervised attention network scales linearly with the hidden dimension and quadratically with the token lengths of protein and ligand sequences. While this introduces higher parameter counts compared to simpler bilinear modules, the explicit attention supervision enables mechanistically explainable residue–level interaction maps without prohibitive computational overhead.

S.2.5 Evaluation of different encoders

Table S8: Summary of foundation models used for encoding proteins and ligands. Protein encoders process either amino acid or structure-aware sequences, while ligand encoders accept SMILES or SELFIES representations.

Foundation Models	Model Name	HuggingFace Link	Input Type
Protein	ProteinBERT	Rostlab/prot_bert	Amino acid sequence
	ESM-2	facebook/esm2_t33_650M_UR50D	Amino acid sequence
	Saprot	westlake-repl/SaProt_650M_AF2	Structure-aware sequence
Ligand	ChemBERTa-2	seyonec/ChemBERTa-zinc-base-v1	SMILES
	MoLFormer	ibm-research/MoLFormer-XL-both-10pct	SMILES
	SELFormer	HUBioDataLab/SELFormer	SELFIES

Descriptions:

- **ProteinBERT** [89]: Pre-trained on large-scale amino acid sequences to capture evolutionary and contextual dependencies within protein families.
- **ESM-2** [78]: An evolutionary-scale protein language model trained on UniRef50, offering deep contextual representations from protein sequence space.
- **Saprot** [77]: Incorporates 3D structural priors by transforming protein structures into structure-aware sequences via `FoldSeek`.
- **ChemBERTa-2** [82]: A transformer-based molecular encoder trained on large SMILES corpora, providing chemically informed embeddings.
- **MoLFormer** [81]: A multi-task transformer model pre-trained on both molecular graphs and SMILES strings, enabling enhanced molecular generalisation.

bioRxiv preprint doi: https://doi.org/10.64898/2026.03.03.707476; this version posted March 5, 2026. The copyright holder for this preprint (which was not certified by peer review) is the author/funder, who has granted bioRxiv a license to display the preprint in perpetuity. It is made available under aCC-BY 4.0 International license.

- **SELFormer** [79]: Utilises SELFIES as a chemically valid representation, improving robustness and representation stability in molecular encoding.

Our framework supports flexible substitution of these encoders with emerging state-of-the-art foundation models, ensuring continual adaptability and performance improvement as the field evolves.

Table S9: Performance comparison of different protein and ligand encoder combinations on the InteractBind (affinity) dataset. Ligand inputs are SMILES or SELFIES, while protein inputs are amino acid sequences or structure-aware representations. We highlight the **best** and second best results.

Ligand Input	Protein Input	Ligand Encoder	Protein Encoder	Accuracy	AUC	AUPR
SMILES	Amino acid sequence	ChemBERTa-2	ProteinBERT	0.884±0.005	0.947±0.004	0.931±0.005
SMILES	Amino acid sequence	ChemBERTa-2	ESM-2	0.891±0.006	0.952±0.003	0.936±0.004
SMILES	Structure-aware sequence	ChemBERTa-2	Saprot	0.907±0.005	0.963±0.003	0.945±0.003
SMILES	Amino acid sequence	MoLFormer	ProteinBERT	0.889±0.006	0.950±0.004	0.934±0.005
SMILES	Amino acid sequence	MoLFormer	ESM-2	0.902±0.005	0.957±0.004	0.939±0.004
SMILES	Structure-aware sequence	MoLFormer	Saprot	0.914±0.004	0.965±0.003	0.948±0.003
SELFIES	Amino acid sequence	SELFormer	ProteinBERT	0.890±0.006	0.949±0.005	0.933±0.004
SELFIES	Amino acid sequence	SELFormer	ESM-2	0.904±0.004	0.958±0.004	0.941±0.004
SELFIES	Structure-aware sequence	SELFormer	Saprot	**0.923±0.004**	**0.971±0.002**	**0.952±0.002**

Accurate token-level fusion is critical for the representational quality of both protein and ligand encoders. As summarized in Table S9, the best overall performance is achieved by combining SELFIES ligands encoded by SELFormer with SA protein sequences encoded by Saprot. This combination achieves the highest scores across all metrics, reflecting the advantages of self-consistent molecular representations on both sides. It is worth noting that SA sequences require extracting structure-aware features from protein structures using FoldSeek [76]. For proteins without experimentally determined structures, we also provide a model trained on the SELFIES & amino-acid sequence combination, which allows users to perform inference using only sequence-level input. This ensures compatibility with sequence-only datasets while maintaining strong performance. In practice, users can choose the appropriate model according to the available molecular inputs. For ligands, both **SMILES** and **SELFIES** are supported, while for proteins, either **structure-aware** or **amino acid** sequences can be used. As indicated in Table S9, the performance degradation from structure-aware to sequence-only setting is limited, confirming the robustness and generalisability of ExplainBind's foundation encoders across different molecular input formats.

S.2.6 Performance on public datasets

To assess further the robustness of ExplainBind, we additionally conducted experiments on three widely used public datasets: **BindingDB** [90], **BioSNAP** [91], and **Human** [92, 93]. In the ID setting, the model is trained solely with binary interaction labels, without the guidance of residue–level attention maps. As shown in Fig. S5, ExplainBind consistently outperforms a wide range of 8 baselines, indicating that it maintains strong predictive capability even without explicit structural supervision.

We further evaluated ExplainBind under OOD scenarios, where training and test sets differ substantially in protein and ligand distributions. The results demonstrate that ExplainBind achieves the best performance in two OOD settings and ranks second in the remaining case, presented in Fig. S6. These findings highlight that ExplainBind generalises well across heterogeneous benchmarks and remains competitive under challenging distribution shifts.

S.2.7 Ablation study

To evaluate the contribution of individual components in ExplainBind, we conducted a comprehensive ablation analysis as summarized in Table S10. All variants share the same configuration as the final ExplainBind model. Replacing the pooling-based fusion with a [CLS] aggregation strategy results in a notable drop in performance (AUC 0.940 $\rightarrow$ 0.927; Accuracy 0.805 $\rightarrow$ 0.784), confirming that mean pooling offers a more stable integration of token-level evidence. When replacing FASTA

bioRxiv preprint doi: https://doi.org/10.64898/2026.03.03.707476; this version posted March 5, 2026. The copyright holder for this preprint (which was not certified by peer review) is the author/funder, who has granted bioRxiv a license to display the preprint in perpetuity. It is made available under aCC-BY 4.0 International license.

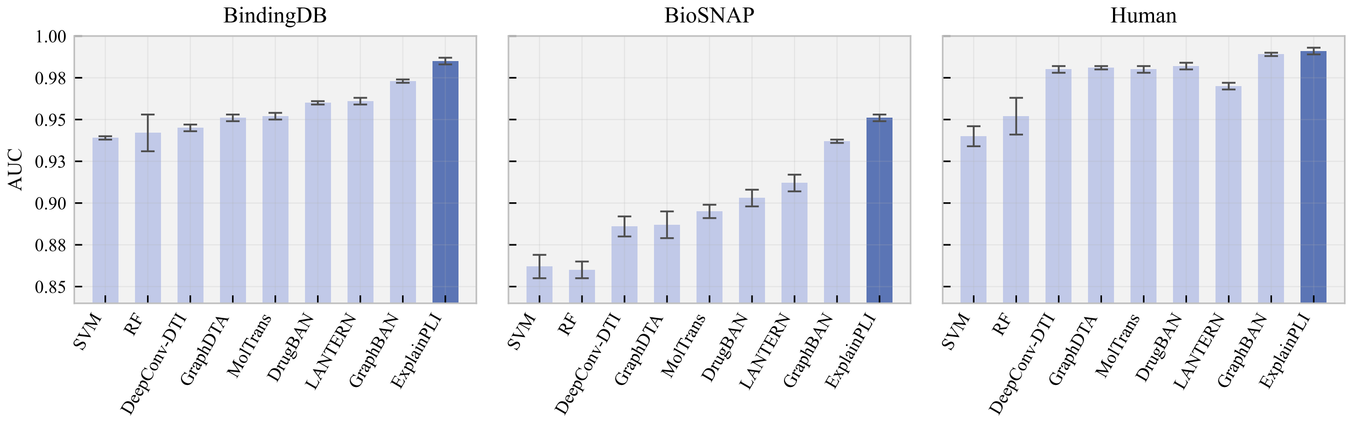

Figure S5: In-distribution performance comparison on three publicly available datasets without attention map supervision.

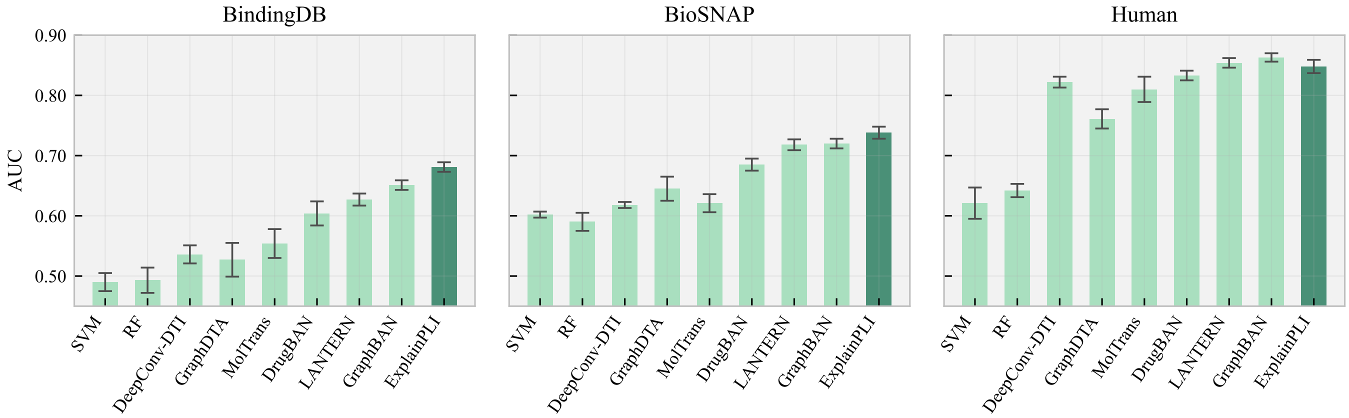

Figure S6: Out-of-distribution performance comparison on three publicly available datasets without attention map supervision.

sequences with SA protein representations, the model achieves the best overall results (AUC 0.947; Accuracy 0.817), suggesting that structural context can further strengthen the learned protein–ligand interactions. However, the SA variant requires structural features extracted through *Foldseek*, which depend on the availability of experimentally resolved or high-confidence predicted protein structures. In practice, many proteins are represented only by their amino acid sequences, making the FASTA-based ExplainBind a more universally applicable model. Overall, these findings demonstrate that pooling aggregation contributes

Table S10: Ablation results on the **PDB (33% OOD)** dataset. All results are based on the same ExplainBind configuration; each row removes or replaces one component for comparison.

**Model / Setting**	**AUC**	**AUPR**	**Accuracy**	**Description**
CLS Aggregation	0.927	0.913	0.784	using [CLS] aggregation (↓ vs ExplainBind)
Pooling Aggregation	0.940	0.926	0.805	using mean pooling for fusion
ExplainBind_SA	0.947	0.933	0.817	variant using structure-aware protein representation (↑ vs ExplainBind)
ExplainBind	0.940	0.926	0.805	using raw FASTA sequence input
KL ratio = 0.1	0.944	0.930	0.930	
KL ratio = 0.2	0.946	0.932	0.927	
KL ratio = 0.3	**0.948**	**0.934**	**0.933**	varying KL loss ratio for attention alignment
KL ratio = 0.4	0.942	0.928	0.921	
KL ratio = 0.5	0.935	0.922	0.901	

bioRxiv preprint doi: https://doi.org/10.64898/2026.03.03.707476; this version posted March 5, 2026. The copyright holder for this preprint (which was not certified by peer review) is the author/funder, who has granted bioRxiv a license to display the preprint in perpetuity. It is made available under aCC-BY 4.0 International license.

to ExplainBind's robust and explainable performance. In Supplementary S.2.5, we further provide detailed comparisons of alternative protein–ligand encoder configurations.

S.2.8 OOD performance comparison

Table S11 reports the exact numerical results underlying the OOD performance line plots presented in the main text. Specifically, the table summarises the OOD performance of ExplainBind and baseline methods on the InteractBind-P datasets under varying OOD ratios (25–33%), evaluated using multiple metrics: AUC, AUPR, Accuracy, F1 score, Sensitivity, Specificity, and MCC [44].

bioRxiv preprint doi: https://doi.org/10.64898/2026.03.03.707476; this version posted March 5, 2026. The copyright holder for this preprint (which was not certified by peer review) is the author/funder, who has granted bioRxiv a license to display the preprint in perpetuity. It is made available under aCC-BY 4.0 International license.

Table S11: OOD performance comparison of ExplainBind and baselines. The **best** and second best results are highlighted. InteractBind-P (25–33% OOD) denote datasets.

Model	AUC	AUPR	Accuracy	F1	Sensitivity	Specificity	MCC
**InteractBind-P (33% OOD)** — Out-of-distribution datasets (decreasing dissimilarity)							
SVM	0.670±0.012	0.620±0.013	0.588±0.011	0.580±0.012	0.572±0.013	0.590±0.011	0.190±0.012
RF	0.698±0.014	0.655±0.015	0.600±0.012	0.590±0.013	0.582±0.014	0.605±0.012	0.198±0.013
DeepConv-DTI	0.768±0.014	0.735±0.016	0.658±0.012	0.645±0.013	0.638±0.015	0.666±0.012	0.215±0.013
GraphDTA	0.782±0.014	0.750±0.016	0.670±0.012	0.660±0.013	0.653±0.015	0.673±0.012	0.225±0.013
MolTrans	0.795±0.015	0.765±0.016	0.678±0.012	0.668±0.013	0.660±0.015	0.682±0.012	0.230±0.013
DrugBAN	0.852±0.010	0.830±0.011	0.718±0.011	0.708±0.012	0.702±0.013	0.720±0.011	0.260±0.011
LANTERN	0.922±0.005	0.900±0.006	0.792±0.007	0.778±0.008	0.774±0.008	0.794±0.007	0.315±0.007
GraphBAN	0.920±0.005	0.904±0.006	0.790±0.007	0.782±0.008	0.772±0.008	0.798±0.007	0.313±0.007
ExplainBind	**0.940±0.004**	**0.926±0.005**	**0.805±0.006**	**0.793±0.007**	**0.788±0.007**	**0.810±0.006**	**0.330±0.007**
**InteractBind-P (31% OOD)**							
SVM	0.660±0.012	0.615±0.014	0.580±0.011	0.572±0.012	0.565±0.014	0.585±0.012	0.185±0.012
RF	0.690±0.015	0.648±0.016	0.595±0.012	0.585±0.013	0.578±0.015	0.600±0.012	0.192±0.013
DeepConv-DTI	0.755±0.014	0.725±0.017	0.646±0.013	0.632±0.014	0.628±0.015	0.655±0.012	0.212±0.013
GraphDTA	0.770±0.014	0.740±0.016	0.660±0.013	0.650±0.014	0.643±0.016	0.663±0.012	0.220±0.014
MolTrans	0.785±0.015	0.755±0.017	0.670±0.012	0.660±0.013	0.653±0.015	0.675±0.011	0.228±0.013
DrugBAN	0.842±0.010	0.820±0.012	0.710±0.011	0.700±0.012	0.695±0.013	0.712±0.011	0.255±0.011
LANTERN	0.912±0.006	0.890±0.007	0.778±0.008	0.768±0.009	0.762±0.009	0.784±0.008	0.304±0.008
GraphBAN	0.910±0.006	0.894±0.007	0.780±0.008	0.766±0.009	0.764±0.009	0.782±0.008	0.306±0.008
ExplainBind	**0.930±0.005**	**0.915±0.006**	**0.795±0.007**	**0.783±0.007**	**0.778±0.008**	**0.800±0.007**	**0.322±0.007**
**InteractBind-P (28% OOD)**							
SVM	0.640±0.012	0.600±0.013	0.570±0.010	0.562±0.012	0.555±0.014	0.575±0.011	0.178±0.012
RF	0.665±0.014	0.625±0.016	0.585±0.012	0.575±0.013	0.570±0.015	0.590±0.012	0.190±0.013
DeepConv-DTI	0.742±0.014	0.710±0.017	0.632±0.013	0.620±0.014	0.615±0.016	0.642±0.012	0.210±0.014
GraphDTA	0.755±0.015	0.725±0.018	0.645±0.014	0.635±0.015	0.628±0.017	0.650±0.013	0.218±0.015
MolTrans	0.768±0.016	0.740±0.017	0.658±0.013	0.648±0.014	0.640±0.016	0.660±0.012	0.225±0.013
DrugBAN	0.825±0.010	0.805±0.011	0.700±0.011	0.688±0.012	0.682±0.013	0.705±0.011	0.250±0.011
LANTERN	0.902±0.006	0.878±0.007	0.770±0.008	0.756±0.009	0.752±0.009	0.772±0.008	0.298±0.008
GraphBAN	0.900±0.006	0.882±0.007	0.768±0.008	0.758±0.009	0.750±0.009	0.774±0.008	0.296±0.008
ExplainBind	**0.910±0.005**	**0.895±0.006**	**0.782±0.007**	**0.770±0.007**	**0.765±0.008**	**0.788±0.007**	**0.310±0.007**
**InteractBind-P (25% OOD)**							
SVM	0.610±0.010	0.580±0.012	0.552±0.009	0.545±0.011	0.540±0.013	0.555±0.010	0.160±0.012
RF	0.628±0.015	0.595±0.016	0.566±0.012	0.558±0.013	0.550±0.015	0.570±0.012	0.172±0.011
DeepConv-DTI	0.680±0.017	0.640±0.018	0.600±0.013	0.592±0.014	0.588±0.016	0.612±0.013	0.190±0.014
GraphDTA	0.702±0.018	0.668±0.020	0.615±0.014	0.605±0.015	0.600±0.016	0.620±0.013	0.200±0.014
MolTrans	0.715±0.020	0.675±0.022	0.622±0.015	0.612±0.016	0.608±0.018	0.624±0.014	0.210±0.015
DrugBAN	0.781±0.011	0.775±0.014	0.689±0.013	0.678±0.012	0.670±0.015	0.715±0.012	0.235±0.013
LANTERN	0.826±0.006	0.793±0.012	0.712±0.005	0.685±0.004	0.684±0.004	0.733±0.012	0.255±0.012
GraphBAN	0.832±0.010	0.803±0.003	0.742±0.003	0.738±0.013	0.722±0.014	0.754±0.012	0.265±0.012
ExplainBind	**0.850±0.006**	**0.836±0.008**	**0.765±0.008**	**0.754±0.009**	**0.748±0.010**	**0.770±0.009**	**0.280±0.009**
